# Global, regional, and national burden of HIV-negative tuberculosis, 1990–2021: findings from the Global Burden of Disease Study 2021

**DOI:** 10.1186/s40249-024-01227-y

**Published:** 2024-08-19

**Authors:** Shun-Xian Zhang, Feng-Yu Miao, Jian Yang, Wen-Ting Zhou, Shan Lv, Fan-Na Wei, Yu Wang, Xiao-Jie Hu, Ping Yin, Pei-Yong Zheng, Ming Yang, Mei-Ti Wang, Xin-Yu Feng, Lei Duan, Guo-Bing Yang, Ji-Chun Wang, Zhen-Hui Lu

**Affiliations:** 1 Longhua Hospital, Shanghai University of Traditional Chinese Medicine, Shanghai, 200032 China; 2 National Institute of Parasitic Diseases at Chinese Center for Disease Control and Prevention (Chinese Center for Tropical Diseases Research), NHC Key Laboratory of Parasite and Vector Biology, WHO Collaborating Centre for Tropical Diseases; National Center for International Research On Tropical Diseases; National Key Laboratory of Intelligent Tracking and Forecasting for Infectious Diseases, Shanghai, 200025 China; 3 Beijing Municipal Health Big Data and Policy Research Center, Beijing Institute of Hospital Management, Beijing, 101100 China; 4 Department of Science and Technology, Chinese Center for Disease Control and Prevention;, National Key Laboratory of Intelligent Tracking and Forecasting for Infectious Diseases, Beijing, 102206 China; 5 National Health Commission (NHC) Key Laboratory of Biosafety, National Institute for Viral Disease Control and Prevention, Chinese Center for Disease Control and Prevention; National Key Laboratory of Intelligent Tracking and Forecasting for Infectious Diseases, Beijing, 102206 China; 6 School of Global Health, Chinese Center for Tropical Diseases Research-Shanghai Jiao Tong University School of Medicine, Shanghai, 200025 China; 7 Shanghai Mental Health Center, Shanghai Jiao Tong University School of Medicine, Shanghai, 200032 China; 8 Gansu Provincial Center for Disease Control and Prevention, Lanzhou, 730000 China

**Keywords:** Tuberculosis, Epidemiology, Global burden of disease, Sociodemographic Index

## Abstract

**Background:**

Tuberculosis (TB) is a major infectious disease with significant public health implications. Its widespread transmission, prolonged treatment duration, notable side effects, and high mortality rate pose severe challenges. This study examines the epidemiological characteristics of TB globally and across major regions, providing a scientific basis for enhancing TB prevention and control measures worldwide.

**Methods:**

The ecological study used data from the Global Burden of Disease (GBD) Study 2021. It assessed new incidence cases, deaths, disability-adjusted life years (DALYs), and trends in age-standardized incidence rates (ASIRs), mortality rates (ASMRs), and DALY rates for drug-susceptible tuberculosis (DS-TB), multidrug-resistant tuberculosis (MDR-TB), and extensively drug-resistant tuberculosis (XDR-TB) from 1990 to 2021. A Bayesian age-period-cohort model was applied to project ASIR and ASMR.

**Results:**

In 2021, the global ASIR for all HIV-negative TB was 103.00 per 100,000 population [95% uncertainty interval (UI): 92.21, 114.91 per 100,000 population], declining by 0.40% (95% UI: − 0.43, − 0.38%) compared to 1990. The global ASMR was 13.96 per 100,000 population (95% UI: 12.61, 15.72 per 100,000 population), with a decline of 0.44% (95% UI: − 0.61, − 0.23%) since 1990. The global age-standardized DALY rate for HIV-negative TB was 580.26 per 100,000 population (95% UI: 522.37, 649.82 per 100,000 population), showing a decrease of 0.65% (95% UI: − 0.69, − 0.57 per 100,000 population) from 1990. The global ASIR of MDR-TB has not decreased since 2015, instead, it has shown a slow upward trend in recent years. The ASIR of XDR-TB has exhibited significant increase in the past 30 years. The projections indicate MDR-TB and XDR-TB are expected to see significant increases in both ASIR and ASMR from 2022 to 2035, highlighting the growing challenge of drug-resistant TB.

**Conclusions:**

This study found that the ASIR of MDR-TB and XDR-TB has shown an upward trend in recent years. To reduce the TB burden, it is essential to enhance health infrastructure and increase funding in low-SDI regions. Developing highly efficient, accurate, and convenient diagnostic reagents, along with more effective therapeutic drugs, and improving public health education and community engagement, are crucial for curbing TB transmission.

**Graphical Abstract:**

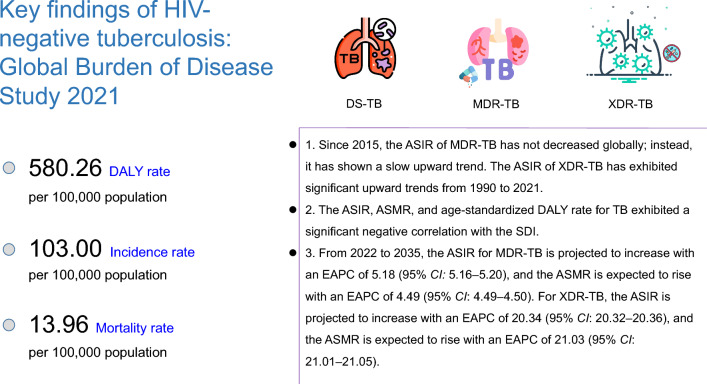

**Supplementary Information:**

The online version contains supplementary material available at 10.1186/s40249-024-01227-y.

## Background

Tuberculosis (TB) is an infectious disease caused by *Mycobacterium tuberculosis* (*Mtb*), primarily affecting the lungs and potentially leading to a chronic, systemic wasting disease. It spreads via airborne droplets through the respiratory tract and remains a significant global public health issue, causing considerable morbidity and mortality [1–3]. According to the World Health Organization (WHO)'s Global Tuberculosis Report 2023, an estimated 10.6 million people [95% uncertainty intervals (UIs): 9.9, 11.4 million] developed TB in 2022, with an incidence rate of 133.0 per 100,000 population. TB caused 1.30 million deaths worldwide in 2022, including 1.13 million deaths among human immunodeficiency virus (HIV)-negative individuals (95% UI: 1.02, 1.26 million) and 0.17 million deaths among people living with HIV (PLWH) (95% UI: 0.14, 0.19 million) [2].

TB can cause long-term damage to the lungs and other organs, leading to various sequelae, complications, and comorbidities [4, 5]. TB can result in structural lung diseases such as obstructive lung disease, bronchiectasis, lung destruction, and atelectasis, as well as permanent lung function impairment, including restrictive, obstructive, and mixed pulmonary dysfunction [4, 5]. Lung function impairment may occur even in asymptomatic patients, increasing all-cause mortality and reducing life expectancy. Additionally, TB has significant physical and psychological impacts on patients [5].

TB remains a significant contributor to global morbidity and mortality despite being preventable and curable. It is the leading cause of death from a single infectious agent [1, 3]. Effective anti-TB drugs, such as rifampicin and isoniazid, have reduced TB incidence globally by 1.9% annually [6]. However, the rise of HIV and drug-resistant TB strains, along with socioeconomic challenges like poverty, conflict, and natural disasters, have hindered progress, falling short of the WHO’s target reduction rate of 4–5% [2, 7, 8].

In low- and middle-income countries (LMICs), TB diagnosis primarily relies on sputum smear microscopy, detecting only 50–60% of cases [9]. Although more sensitive diagnostic methods and drug resistance tests, such as next-generation sequencing, are available, their high costs limit widespread application in high-burden countries [10]. Delays in diagnosis and treatment initiation significantly contribute to TB transmission [11].

TB treatment regimens, requiring multiple drugs over several months, pose challenges for patients and healthcare systems, particularly in LMICs. The rising incidence of drug-resistant TB necessitates longer, more expensive, and less tolerable treatment courses, exacerbating transmission [12]. Additionally, the spread of HIV/acquired immune deficiency syndrome (AIDS) complicates TB control, with 6.3% of new TB cases being HIV-positive in 2022, and co-infection rates reaching 50.0% in some sub-Saharan African regions [2]. If current trends continue, achieving the 2030 Sustainable Development Goals (SDGs) and the WHO End TB Strategy—aiming for a 90% reduction in TB mortality and an 80% reduction in incidence from 2015 levels, while eliminating catastrophic costs for TB-affected households—will be challenging [13].

A comprehensive understanding of the burden and epidemiological trends of TB is crucial for assessing progress toward TB elimination and guiding the formulation of control policies and strategies. The Global Burden of Disease (GBD) Study 2021, one of the most comprehensive observational epidemiologic studies globally, provides essential data to explore and understand the epidemiological characteristics of HIV-negative TB [1, 14, 15]. The study aims to detail the epidemiologic features of TB, drug-susceptible TB (DS-TB), multidrug-resistant TB (MDR-TB), and extensively drug-resistant TB (XDR-TB) on a global scale and across major geographic regions. The findings underscore the urgency of TB control within the global health framework and provide a scientific basis for developing more effective public health strategies and programs to curb TB transmission.

## Methods

### Date source

The GBD 2021 comprehensively evaluated the burden of diseases, injuries, and risk factors across different age and gender groups globally. It provided data on 371 diseases or injuries and 88 risk factors from 204 countries and territories spanning 1990 to 2021 [15]. The GBD 2021 project estimated the rates, numbers and percentages change of incidences, prevalences, deaths and disability adjusted life years (DALYs) for diseases, details of these estimated indices were presented in the appendix of the GBD 2021 capstone paper [1].

The GBD 2021 estimates for the global TB burden have been updated with improved data sources, estimation models, and statistical analysis methods, adhering to the Guidelines for Accurate and Transparent Health Estimates Reporting [15, 16]. The Disease-Model Bayesian Meta-Regression (DisMoD-MR) tool (version 2.1), based on the Bayesian Priors, Regularisation, and Trimming (MR-BRT) model, utilizes all available morbidity and mortality data, epidemiological relationships, and spatial relationships to provide consistent disease burden estimates. Detailed information on the design, data collection, and estimation methods for TB and its subtypes in the GBD study 2021 is available elsewhere [1, 15, 16].

For HIV-negative TB, DS-TB, MDR-TB, and XDR-TB, data on annual incident cases, incidence rates, number of deaths, mortality rates, DALY numbers, and DALY rates from 1990 to 2021 were obtained from the Global Health Data Exchange tool, categorized by year, age, gender, region, and country (https://ghdx.healthdata.org). Notably, data on the age-standardized mortality rates (ASMRs) and death cases for XDR-TB were available from 1993 [1]. Therefore, data from 2010 to 2021 were used to analyze the annual changes in age-standardized incidence rates (ASIRs), ASMRs, and age-standardized DALY rates for XDR-TB. For TB, DS-TB, and MDR-TB, data from 1990 to 2021 were used to assess annual average rate changes [1].

The Socio-demographic Index (SDI) represents the combined level of health-related social and economic conditions in each region. SDI is a composite measure derived from the total fertility rate in women under 25 years, the mean education level in individuals aged 15 years and older, and per capita income. SDI values range from 0.00 to 1.00 and are multiplied by 100. Countries and territories in the GBD 2021 are grouped into five development levels: low (< 0.46), low-middle (0.46–0.60), middle (0.61–0.69), high-middle (0.70–0.81), and high (> 0.81) [15, 16].

The GBD 2021 estimated mortality and DALYs for various risk factors and their combinations across different geographical units, providing a standardized and comprehensive assessment of risk exposure and attributable burden [15, 16]. Data on ASMR and age-standardized DALY rates due to risk factors were categorized under level 2, including air pollution, alcohol use, child and maternal malnutrition, childhood sexual abuse and bullying, dietary risks, drug use, high body-mass index, high fasting plasma glucose, high low density lipoprotein cholesterol, high systolic blood pressure, intimate partner violence, kidney dysfunction, low bone mineral density, low physical activity, non-optimal temperature, occupational risks, other environmental risks, tobacco use, unsafe sex, unsafe water, sanitation and hand washing [15, 16].

### Case definition

The classification of TB follows the International Statistical Classification of Diseases and Related Health Problems (ICD). This includes all forms of TB, both pulmonary and extrapulmonary, whether bacteriologically confirmed or clinically diagnosed. The relevant ICD-10 codes for TB are A10–A14, A15–A19.9, B90–B90.9, K67.3, K93.0, M49.0, N74.1, P37.0, and U84.3. The corresponding ICD-9 codes are 010–019.9, 137–137.9, 138.0, 138.9, 320.4, and 730.4–730.6. For HIV-associated TB, the ICD-10 code is B20.0 [1, 15].

DS-TB is defined as TB that is susceptible to isoniazid and rifampicin. MDR-TB without extensive drug resistance is defined as TB that is resistant to isoniazid and rifampicin but not resistant to any fluoroquinolone or second-line injectable drug. XDR-TB is defined as TB that is resistant to isoniazid and rifampicin, any fluoroquinolone, and at least one second-line injectable drug [1, 2, 16].

### Statistical analysis

The disease burden of TB, DS-TB, MDR-TB, and XDR-TB among HIV-negative individuals was quantified by ASIRs, ASMRs, and age-standardized DALYs, along with the numbers of incidence, death, and DALY. Age-standardized rates (ASRs), specific rates for various age groups, and corresponding numbers were extracted from the GBD 2021 database, represented as estimated values with 95% UIs [17]. The formula for calculating the ASR is:

$$ASR = \frac{{\sum\nolimits_{i = 1}^{N} {a_{i} w_{i} } }}{{\sum\nolimits_{i = 1}^{N} {wi} }}$$

where $a_{i}$ the age-specific rate in the $i$th age group and $w_{{\text{i}}}$ represents the number of persons (or the weight) in the same age group among the GBD 2021 standard population. $N$ is the number of age groups. 95% UIs were defined as the 2.5th and 97.5th percentile values of the ordered 1000 draws.

The percentage change in incidence, death, and DALY numbers and rates from 1990 to 2021 was calculated using the equation [18]:

Percentage changes = (value _behind_−value _before_)/value _before_ × 100%. The GBD 2021 database used UIs instead of precise statistical values. Consequently, when comparing two numerical values (numbers, rates, or percentages), statistical significance could not be directly calculated. If the UIs overlapped, it indicated no significant difference (*P* > 0.05). Conversely, if the UIs did not overlap, a statistical difference existed (*P* < 0.05).

Smoothing spline models were used to evaluate the relationship between the ASRs (ASIRs, ASMRs, and age-standardized DALY rates) of HIV-negative TB, DS-TB, MDR-TB, and XDR-TB and the SDI across 21 geographical regions and 204 countries and territories. Locally Weighted Scatterplot Smoothing was applied to fit the splines, automatically determining the degree, number, and location of knots based on the data and the span parameter. Spearman's rank correlation coefficient was used to verify the correlations between ASRs and SDI. A *P*-value of less than 0.05 was considered statistically significant.

For the ASIRs, ASMRs, and age-standardized DALY rates from 1990 to 2021, the estimated annual percentage changes (EAPCs) were calculated to depict trends in HIV-negative TB, DS-TB, MDR-TB, and XDR-TB using a linear regression model $${\text{ln}}(ASR) = \alpha + \beta x + \varepsilon$$$$x$$ signifies the calendar year, and $$\varepsilon$$ denotes an independent, normally distributed error term [19]. Then, the EAPC is equal to $$100 \times (e^{\beta } - 1)$$, the EAPCs and their 95% confidence intervals (*CI*s) are utilized to describe trends over specified time intervals [τ_*j—1*_, τ_*j*_]. If the upper limit of the EAPC (95% *CI*) is less than zero, the rate exhibits a statistically significant decline over the observed period. Conversely, if the lower limit of the EAPC (95% *CI*) is greater than zero, the rate shows a statistically significant increase. When the 95% *CI* of EAPC includes zero, the change of the ASRs is considered statistically non-significant, indicating no meaningful trend. Two-tailed tests were used for all statistical assessments, and the significance level was set at *P* < 0.05.

The Bayesian age-period-cohort model (BAPC, with the default parameters) examined the multiplicative effects of age, period, and cohort [20, 21]:

$n_{ij} = \mu + \alpha_{i} + \beta_{j} + \gamma_{k}$ In the model, $n_{ij}$ stand for the ASR, $\mu$ denotes the intercept, and $\alpha_{i}$ and $\gamma_{k}$ were age, period, and cohort effects, respectively. BAPC model was implemented with the integrated nested Laplace approximation (INLA) and BAPC packages in R software [20, 21].

All statistical analyses were conducted using R software (version 4.4.1. R Foundation for Statistical Computing, Vienna, Austria, https://cran.r-project.org).

## Results

### Incidence and temporal trend

In 2021, the global ASIR for all HIV-negative TB was 103.00 per 100,000 population (95% UI: 92.21, 114.91 per 100,000 population), reflecting a percentage change of − 0.40% (95% UI: − 0.43, − 0.38%) compared to 1990. The ASIR for DS-TB was 97.29 per 100,000 population (95% UI: 85.79, 110.48 per 100,000 population), with a decline of − 0.43% (95% UI: − 0.47, − 0.41%) compared to 1990. For MDR-TB, the ASIR was 5.42 per 100,000 population (95% UI: 3.17, 9.34 per 100,000 population), showing an increase of 4.09% (95% UI: 0.99, 12.15%) compared to 1990. XDR-TB had an ASIR of 0.29 per 100,000 population (95% UI: 0.21, 0.42 per 100,000 population), with a percentage change of 0.03% (95% UI: − 0.25, 0.47%) compared to 1990 (Table 1). In addition, the EAPC for ASIR from 1990 to 2021 were − 1.91 (95% *CI:* − 2.01, − 1.82) for TB, − 2.40 (95% *CI: *− 2.21, − 1.97) for DS-TB, and 2.05 (95% *CI:* 0.58, 3.54) for MDR-TB, respectively, from 1990 to 2021.

**Table 1:** ASIR of TB, DS-TB, MDR-TB, and XDR-TB in HIV-negative individuals in 2021, and percentage change of ASIR were analyzed across GBD regions

Regions	HIV-negative TB		DS-TB		MDR-TB		XDR-TB	
	ASIR (per 100,000 population) (95% UI) 2021	Percentage change of ASIR (95% UI) 1990–2021	ASIR (per 100,000 population) (95% UI) 2021	Percentage change of ASIR (95% UI) 1990–2021	ASIR (per 100,000 population) (95% UI) 2021	Percentage change of ASIR (95% UI) 1990–2021	ASIR (per 100,000 population) (95% UI) 2021	Percentage change of ASIR (95% UI) 2010–2021
Global	103.00 (92.21, 114.91)	− 0.40 (− 0.43, − 0.38)	97.29 (85.79, 110.48)	− 0.43 (− 0.47, − 0.41)	5.42 (3.17, 9.34)	4.09 (0.99, 12.15)	0.29 (0.21, 0.42)	0.03 (− 0.25, 0.47)
Male	115.34 (103.71, 128.58)	− 0.39 (− 0.42, − 0.36)	108.77 (96.34, 123.53)	− 0.42 (− 0.42, − 0.39)	6.20 (3.68, 10.59)	3.81 (3.81, 11.37)	0.37 (0.27, 0.54)	0.01 (− 0.27, 0.40)
Female	91.96 (81.49, 102.62)	− 0.42 (− 0.45, − 0.40)	87.04 (76.09, 98.73)	− 0.45 (− 0.45, − 0.43)	4.70 (2.69, 8.35)	4.42 (4.42, 13.15)	0.21 (0.15, 0.31)	0.08 (− 0.23, 0.57)
East Asia	40.28 (36.25, 44.72)	− 0.64 (− 0.66, − 0.62)	38.46 (33.09, 43.35)	− 0.65 (− 0.68, − 0.62)	1.67 (0.43, 4.73)	− 0.50 (− 0.90, 1.27)	0.15 (0.04, 0.42)	− 0.16 (− 0.75, 1.14)
Southeast Asia	181.41 (164.65, 198.8)	− 0.42 (− 0.44, − 0.40)	177.5 (161.10, 194.22)	− 0.43 (− 0.45, − 0.42)	3.55 (1.87, 6.11)	3.35 (0.42, 10.54)	0.37 (0.20, 0.64)	− 0.04 (− 0.52, 0.75)
Oceania	122.03 (111.69, 133.54)	− 0.24 (− 0.28, − 0.20)	116.92 (106.56, 128.33)	− 0.27 (− 0.32, − 0.23)	4.47 (1.33, 10.23)	44.57 (6.85, 246.15)	0.64 (0.19, 1.49)	2.40 (− 0.23, 10.27)
Central Asia	53.28 (46.65, 60.59)	− 0.45 (− 0.49, − 0.40)	39.87 (33.51, 47.29)	− 0.59 (− 0.64, − 0.53)	11.07 (7.45, 15.82)	65.97 (18.74, 205.01)	2.35 (1.59, 3.35)	− 0.12 (− 0.42, 0.24)
Central Europe	13.64 (11.86, 15.67)	− 0.61 (− 0.64, − 0.58)	13.33 (11.59, 15.36)	− 0.62 (− 0.65, − 0.58)	0.26 (0.12, 0.50)	0.33 (− 0.54–2.81)	0.06 (0.03, 0.11)	− 0.18 (− 0.71, 1.13)
Eastern Europe	57.89 (48.95, 70.52)	− 0.42 (− 0.46, − 0.34)	37.64 (29.22, 48.03)	− 0.62 (− 0.69, − 0.54)	16.73 (10.38, 24.72)	14.12 (4.44–38.75)	3.52 (2.18, 5.20)	0.06 (− 0.36, 0.67)
High-income Asia Pacific	15.44 (13.33, 17.83)	− 0.66 (− 0.69, − 0.63)	15.25 (13.14, 17.66)	− 0.66 (− 0.70, − 0.63)	0.17 (0.05, 0.55)	− 0.34 (− 0.87, 2.06)	0.02 (0.01, 0.07)	− 0.07 (− 0.71, 1.79)
Australasia	5.13 (4.37, 6.01)	− 0.40 (− 0.44, − 0.35)	4.96 (4.22, 5.84)	− 0.42 (− 0.46, − 0.36)	0.15 (0.06, 0.32)	2.48 (− 0.07, 15.23)	0.02 (0.01, 0.04)	1.54 (− 0.30, 7.57)
Western Europe	5.69 (4.79, 6.76)	− 0.56 (− 0.59, − 0.54)	5.55 (4.67, 6.59)	− 0.57 (− 0.60, − 0.55)	0.13 (0.08, 0.21)	0.29 (− 0.31, 1.49)	0.02 (0.01, 0.03)	0.26 (− 0.17, 1.06)
Southern Latin America	13.63 (11.79, 15.97)	− 0.51 (− 0.55, − 0.47)	13.43 (11.58, 15.76)	− 0.51 (− 0.55, − 0.47)	0.17 (0.05, 0.54)	0.85 (− 0.66, 7.55)	0.02 (0.01, 0.08)	0.13 (− 0.66, 1.92)
High-income North America	2.31 (1.99, 2.73)	− 0.44 (− 0.47, − 0.41)	2.27 (1.95, 2.68)	− 0.44 (− 0.47, − 0.40)	0.03 (0.01, 0.08)	− 0.72 (− 0.90, − 0.17)	0.00 (0.00, 0.01)	0.68 (− 0.32, 3.07)
Caribbean	34.13 (30.04, 38.28)	− 0.32 (− 0.36, − 0.27)	33.96 (29.90, 38.12)	− 0.32 (− 0.36, − 0.27)	0.16 (0.06, 0.38)	− 0.23 (− 0.80, 1.63)	0.01 (0.00, 0.04)	0.85 (− 0.40, 4.09)
Andean Latin America	61.55 (53.60, 71.69)	− 0.69 (− 0.71, − 0.66)	57.00 (49.38, 67.20)	− 0.71 (− 0.74, − 0.68)	0.92 (0.21, 2.64)	1.56 (–0.16, 7.29)	0.35 (0.18, 0.69)	0.27 (− 0.34, 1.37)
Central Latin America	19.25 (16.92, 21.93)	− 0.55 (− 0.58, − 0.52)	18.64 (16.34, 21.36)	− 0.56 (− 0.60, − 0.53)	0.57 (0.24, 1.13)	7.84 (1.90, 25.03)	0.05 (0.02, 0.09)	0.45 (− 0.29, 1.71)
Tropical Latin America	29.53 (25.53, 34.40)	− 0.46 (− 0.50, − 0.42)	28.54 (24.31, 33.39)	− 0.48 (− 0.53, − 0.44)	4.20 (2.07, 8.29)	34.16 (3.63, 281.56)	0.07 (0.02, 0.21)	0.87 (− 0.53, 4.22)
North Africa and Middle East	28.94 (25.47, 32.99)	− 0.60 (− 0.63, − 0.57)	28.06 (24.54, 32.01)	− 0.61 (− 0.64, − 0.59)	0.84 (0.47, 1.55)	3.44 (0.78, 11.04)	0.04 (0.02, 0.08)	− 0.15 (− 0.60, 0.75)
South Asia	204.05 (180.62, 231.66)	− 0.50 (− 0.54, − 0.46)	189.19 (161.61, 220.04)	− 0.54 (− 0.59, − 0.49)	14.46 (4.55, 32.75)	35.35 (4.84, 179.68)	0.40 (0.13, 0.91)	0.40 (− 0.49, 2.16)
Central sub-Saharan Africa	392.31 (352.37, 437.48)	− 0.28 (− 0.31, − 0.25)	382.38 (339.78, 425.41)	− 0.30 (− 0.33, − 0.26)	9.83 (2.91, 25.87)	4.34 (0.09, 27.42)	0.10 (0.03, 0.26)	0.23 (− 0.61, 2.94)
Eastern sub-Saharan Africa	282.94 (250.78, 314.99)	− 0.51 (− 0.53, − 0.48)	271.21 (240.14, 302.70)	− 0.53 (− 0.55, − 0.50)	11.61 (6.94, 19.19)	18.98 (5.94, 50.59)	0.12 (0.07, 0.19)	0.40 (− 0.17, 1.29)
Southern sub-Saharan Africa	417.09 (370.35, 470.4)	− 0.23 (− 0.29, − 0.17)	401.31 (352.70, 454.55)	− 0.26 (− 0.32, − 0.20)	15.65 (7.14, 32.63)	6.06 (0.98, 28.89)	0.13 (0.06, 0.29)	0.15 (− 0.54, 1.73)
Western sub-Saharan Africa	177.75 (155.88, 200.88)	− 0.51 (− 0.54, − 0.47)	171.17 (149.53, 193.21)	− 0.53 (− 0.56, − 0.49)	6.52 (3.06, 13.88)	4.91 (1.28, 14.69)	0.06 (0.03, 0.13)	− 0.05 (− 0.50, 0.90)
High-middle SDI	35.45 (31.53, 40.33)	− 0.56 (− 0.58, − 0.54)	31.02 (26.95, 35.55)	− 0.61 (− 0.63, − 0.58)	3.77 (2.33, 5.85)	1.57 (− 0.04, 6.61)	0.66 (0.41, 0.95)	− 0.07 (− 0.41, 0.37)
High SDI	9.19 (8.04, 10.61)	− 0.57 (− 0.59, − 0.55)	8.96 (7.80, 10.38)	− 0.58 (− 0.60, − 0.55)	0.21 (0.12, 0.38)	− 0.18 (− 0.60, 0.69)	0.02 (0.01, 0.04)	0.02 (− 0.36, 0.72)
Low-middle SDI	184.63 (164.33, 208.36)	− 0.50 (− 0.53, − 0.47)	173.93 (152.62, 199.25)	− 0.53 (− 0.57, − 0.5)	10.35 (4.21, 21.99)	24.56 (6.35, 79.12)	0.36 (0.18, 0.70)	0.25 (− 0.35, 1.38)
Low SDI	240.81 (214.04, 269.42)	− 0.46 (− 0.48, − 0.44)	229.44 (203.43, 257.18)	− 0.48 (− 0.51, − 0.46)	11.13 (6.51, 19.05)	10.68 (4.20, 25.42)	0.23 (0.12, 0.47)	0.29 (− 0.41, 1.87)
Middle SDI	97.92 (88.07, 107.77)	− 0.42 (− 0.45, − 0.40)	93.04 (83.33, 103.82)	− 0.44 (− 0.48, − 0.41)	4.63 (2.22, 8.39)	1.46 (− 0.22, 7.57)	0.25 (0.15, 0.40)	0.09 (− 0.34, 0.81)

Globally, the World Health Organization began to recommend the XDR-TB surveillance in 1991. Consequently, the ASIR of XDR-TB has been tracked and reported since 1991. However, the GBD 2021 database provides total percentage change data for the periods 1990–2000, 2000–2021, 1990–2021, 2010–2021, and 2019–2021. Therefore, percentage change of ASIR for XDR-TB spanning 2010–2021 were used in the study

*ASIR* Age-standardized incidence rate, *DS-TB* drug-susceptible tuberculosis, *GBD* Global Burden of Disease, *HIV* human immunodeficiency virus, *MDR-TB* multidrug-resistant tuberculosis without extensive drug resistance, *SDI* Sociodemographic Index, *TB* Tuberculosis, *UI* Uncertainty interval, *XDR-TB* extensively drug-resistant tuberculosis

In 2021, the global incidence of TB was 8.41 million cases (95% UI: 7.52, 9.39 million), with DS-TB accounting for 7.94 million cases (95% UI: 7.01, 9.02 million). MDR-TB accounted for 0.44 million cases (95% UI: 0.26, 0.77 million), and XDR-TB accounted for 24,036 cases (95% UI: 17,144, 34,587 persons. Additional file 1: Table S1).

In 2021, the ASIR for all HIV-negative TB was 115.34 per 100,000 population (95% UI: 103.71, 128.58 per 100,000 population) in males, declining by − 0.39% (95% UI: − 0.42, − 0.36%) compared to 1990. For females, the ASIR was 91.96 per 100,000 population (95% UI: 81.49, 102.62 per 100,000 population) in female, declining by − 0.42% (95% UI: − 0.45, − 0.40%) compared to 1990 (Table 1). The ASIR for TB, DS-TB, MDR-TB, and XDR-TB showed no significant differences between males and females (*P* > 0.05). Compared to 1990, the ASIR of TB and DS-TB declined in both genders by 2021. However, the ASIR of MDR-TB increased in both genders in 2021. For XDR-TB, the ASIR did not show a significant trend in either direction in males or females in 2021 compared to 2010 (all *P* > 0.05. Table 1).

In 2021, the ASIR of TB, DS-TB, and MDR-TB were highest in low SDI regions and lowest in high SDI regions. Conversely, the ASIR of XDR-TB was highest in high-middle SDI countries and lowest in high SDI regions (Table 1). Over the past 30 years, the ASIR of TB and DS-TB declined across all SDI categories (Additional file 1: Table S2). For MDR-TB, the ASIR declined in high SDI regions (EAPC = − 3.33, 95% *CI:* − 4.30, − 2.26). Conversely, ASIR increased in low-middle SDI (EAPC = 6.02, 95% *CI: *3.61, 6.49) and low SDI (EAPC = 4.39, 95% *CI:* 2.42, 6.40) regions. XDR-TB demonstrated an increase in ASIR across all SDI categories, regions with lower SDI values exhibited greater increases in ASIR. For example, low SDI regions had the highest increase in ASIR (EAPC = 15.30, 95% *CI: *10.90, 19.67. Additional file 1: Table S2).

In 2021, the ASIR of TB and DS-TB was highest in Southern sub-Saharan Africa and lowest in high-income North America. The highest ASIR of MDR-TB was observed in Eastern Europe, while the lowest was in high-income North America. Similarly, the ASIR of XDR-TB was highest in Eastern Europe (Table 1). From 1990 to 2021. The ASIR of TB and DS-TB showed a global decline across all 21 regions, with the most significant reductions observed in Andean Latin America (*P* < 0.05). However, the ASIR of MDR-TB increased in several regions, including Southeast Asia, Oceania, Central Asia, Eastern Europe, Central Latin America, Tropical Latin America, North Africa and the Middle East, South Asia, and various sub-Saharan African regions (all *P* < 0.05). The most substantial increase was in Central Asia (*P* < 0.05), while high-income North America experienced the largest decrease in ASIR of MDR-TB (*P* < 0.05. Table 1).

In 2021, Somalia had the highest ASIR of TB, DS-TB, and MDR-TB, and the Republic of Moldova had the highest ASIR of XDR-TB (Additional file 1: Table S3). Compared to 1990, the ASIR of TB and DS-TB decreased in all countries and regions except for the Philippines (all *P* < 0.05. Additional file 1: Table S3). Significant increases in ASIR of MDR-TB were observed in Kyrgyzstan. In addition, the most substantial increase in ASIR of XDR-TB was recorded in Papua New Guinea in 2021 compared to 2010 (*P* < 0.05. Additional file 1: Table S3).

### Death and temporal trend

In 2021, the global ASMR of TB was 13.96 per 100,000 population (95% UI: 12.61, 15.72 per 100,000 population), reflecting a decline of – 0.44% (95% UI: − 0.61, − 0.23%) compared to 1990. The ASMR for DS-TB, MDR-TB, and XDR-TB in 2021 were 12.58 per 100,000 population (95% UI: 10.91, 14.42 per 100,000 population), 1.28 per 100,000 population (95% UI: 0.50, 2.53 per 100,000 population), and 0.09 per 100,000 population (95% UI: 0.04, 0.18 per 100,000 population), respectively. DS-TB showed a decline in ASMR of 0.49% (95% UI: − 0.63, − 0.28%) compared to 1990, while the ASMR of MDR-TB increased substantially by 36.08% (95% UI: 5.11, 208.98%) compared to 1990, and the ASMR of XDR-TB exhibited a percentage change of 2.24% (95% UI: − 0.26, 9.60%) compared to 2010 (Table 2). The EAPC for ASMR from 1990 to 2021 were − 3.53 (95% *CI:* − 3.71, – 3.36%) for TB, − 3.79% (95% *CI: *− 3.92, − 3.67) for DS-TB, and 1.18 (95% *CI:* − 0.43, 2.82) for MDR-TB, respectively.

**Table 2:** ASMR of TB, DS-TB, MDR-TB, and XDR-TB in HIV-negative individuals in 2021, and percentage change of ASMR were analyzed across GBD regions

Regions	HIV-negative TB		DS-TB		MDR-TB		XDR-TB	
	ASMR (per 100,000 population) (95% UI) 2021	Percentage change of ASMR (95% UI) 1990–2021	ASMR (per 100,000 population) (95% UI) 2021	Percentage change of ASMR (95% UI) 1990–2021	ASMR (per 100,000 population) (95% U*I*) 2021	Percentage change of ASMR (95% UI) 1990–2021	ASMR (per 100,000 population) (95% UI) 2021	Percentage change of ASMR (95% UI) 2010–2021
Global	13.96 (12.61, 15.72)	− 0.65 (− 0.69, − 0.55)	12.58 (10.91, 14.42)	− 0.68 (− 0.73, − 0.59)	1.28 (0.50, 2.53)	2.45 (0.37, 7.39)	0.09 (0.04, 0.18)	− 0.21 (− 0.45, 0.10)
Male	18.19 (16.16, 21.80)	− 0.65 (− 0.72, − 0.47)	16.41 (13.92, 19.67)	− 0.68 (− 0.75, − 0.52)	1.65 (0.66, 3.26)	2.36 (0.34, 7.87)	0.13 (0.06, 0.25)	− 0.25 (− 0.47, 0.07)
Female	10.22 (9.28, 11.33)	− 0.66 (− 0.71, − 0.62)	9.21 (8.01, 10.41)	− 0.70 (− 0.74, − 0.65)	0.95 (0.37, 1.88)	2.49 (0.43, 7.73)	0.06 (0.02, 0.12)	− 0.13 (− 0.42, 0.30)
East Asia	2.43 (1.99, 3.04)	− 0.88 (− 0.91, − 0.83)	2.20 (1.68, 2.76)	− 0.89 (− 0.92, − 0.84)	0.19 (0.05, 0.48)	− 0.83 (− 0.96, -0.33)	0.04 (0.01, 0.10)	− 0.37 (− 0.76, 0.45)
Southeast Asia	27.25 (23.53, 31.56)	− 0.69 (− 0.74, − 0.55)	26.12 (22.56, 30.36)	− 0.70 (− 0.75, − 0.57)	0.95 (0.35, 2.03)	1.06 (− 0.28, 4.93)	0.19 (0.06, 0.43)	− 0.28 (− 0.61, 0.28)
Oceania	36.51 (29.41, 45.06)	− 0.44 (− 0.61, − 0.23)	33.69 (26.45, 41.69)	− 0.49 (− 0.63, − 0.28)	2.36 (0.52, 6.26)	36.08 (5.11, 208.98)	0.46 (0.10, 1.26)	2.24 (− 0.26, 9.60)
Central Asia	4.67 (4.12, 5.27)	− 0.62 (− 0.67, − 0.57)	2.90 (1.94, 3.78)	− 0.76 (-0.84, − 0.69)	1.21 (0.67, 1.86)	26.92 (9.34, 81.81)	0.56 (0.31, 0.90)	− 0.42 (− 0.59, − 0.22)
Central Europe	0.99 (0.91, 1.07)	− 0.79 (− 0.80, − 0.77)	0.94 (0.86, 1.02)	− 0.80 (− 0.81, − 0.78)	0.03 (0.01, 0.07)	− 0.35 (− 0.77, 0.85)	0.02 (0.01, 0.04)	− 0.40 (− 0.79, 0.57)
Eastern Europe	2.95 (2.67, 3.31)	− 0.47 (− 0.52, − 0.41)	1.54 (0.91, 2.20)	− 0.72 (− 0.83, − 0.60)	0.97 (0.55, 1.39)	5.60 (1.62, 16.99)	0.45 (0.25, 0.70)	− 0.49 (− 0.65, -0.25)
High-income Asia Pacific	1.10 (0.93, 1.23)	− 0.84 (− 0.87, − 0.82)	1.07 (0.89, 1.20)	− 0.85 (− 0.87, − 0.83)	0.02 (0.00, 0.07)	− 0.75 (− 0.95, 0.04)	0.01 (0.00, 0.02)	− 0.19 (− 0.75, 1.43)
Australasia	1.49 (1.39, 1.60)	− 0.76 (− 0.78, − 0.74)	1.45 (1.33, 1.57)	− 0.77 (− 0.80, − 0.75)	0.04 (0.01, 0.11)	0.26 (− 0.68, 4.32)	0.00 (0.00, 0.01)	0.69 (− 0.53, 4.59)
Western Europe	0.14 (0.13, 0.15)	− 0.82 (− 0.84, − 0.82)	0.13 (0.11, 0.15)	− 0.83 (− 0.84, − 0.82)	0.01 (0.00, 0.02)	− 0.51 (− 0.73, − 0.04)	0.00 (0.00, 0.01)	− 0.13 (− 0.42, 0.36)
Southern Latin America	0.27 (0.24, 0.29)	− 0.75 (− 0.77, − 0.73)	0.26 (0.23, 0.28)	− 0.76 (− 0.78, − 0.73)	0.01 (0.01, 0.02)	− 0.11 (− 0.82, 2.81)	0.01 (0.00, 0.03)	− 0.05 (− 0.71, 1.34)
High-income North America	0.15 (0.14, 0.16)	− 0.79 (− 0.80, − 0.79)	0.14 (0.13, 0.15)	− 0.79 (− 0.80, − 0.77)	0.00 (0.00, 0.01)	− 0.90 (− 0.97, − 0.70)	0.00 (0.00, 0.03)	0.46 (− 0.40, 2.66)
Caribbean	7.05 (5.66, 8.92)	− 0.60 (− 0.71, − 0.44)	6.00 (4.37, 7.78)	− 0.60 (− 0.71, − 0.44)	0.89 (0.32, 1.97)	− 0.48 (− 0.90, 1.01)	0.01 (0.00, 0.03)	0.69 (− 0.47, 3.58)
Andean Latin America	2.41 (2.13, 2.75)	− 0.86 (− 0.89, − 0.82)	2.25 (1.95, 2.60)	− 0.88 (− 0.92, − 0.84)	0.14 (0.05, 0.30)	0.09 (− 0.62, 2.66)	0.15 (0.05, 0.35)	− 0.01 (− 0.49, 0.88)
Central Latin America	5.07 (3.37, 12.21)	− 0.82 (− 0.84, − 0.80)	5.01 (3.34, 12.07)	− 0.84 (− 0.86, − 0.81)	0.05 (0.01, 0.15)	2.96 (0.40, 9.60)	0.02 (0.01, 0.05)	0.16 (− 0.41, 1.12)
Tropical Latin America	2.29 (2.18, 2.38)	− 0.71 (− 0.72, − 0.70)	2.13 (1.78, 2.30)	− 0.73 (− 0.77, − 0.71)	0.13 (0.03, 0.39)	14.68 (1.29, 117.25)	0.02 (0.00, 0.07)	0.38 (− 0.61, 2.63)
North Africa and Middle East	4.37 (3.44, 6.33)	− 0.76 (− 0.81, − 0.65)	4.07 (3.10, 5.96)	− 0.77 (− 0.82, − 0.67)	0.28 (0.09, 0.70)	2.47 (0.26, 8.38)	0.02 (0.01, 0.06)	− 0.26 (− 0.64, 0.66)
South Asia	81.60 (67.28, 98.09)	− 0.71 (− 0.75, − 0.61)	75.31 (61.01, 91.46)	− 0.75 (− 0.82, − 0.65)	6.21 (2.40, 12.52)	17.22 (2.42, 86.95)	0.22 (0.05, 0.53)	0.17 (− 0.55, 1.46)
Central sub-Saharan Africa	102.62 (73.09, 148.92)	− 0.46 (− 0.59, − 0.27)	97.45 (68.51, 142.04)	− 0.49 (− 0.61, − 0.30)	5.10 (1.24, 17.43)	3.31 (− 0.14, 21.53)	0.07 (0.02, 0.24)	0.19 (− 0.59, 2.63)
Eastern sub-Saharan Africa	33.11 (29.19, 39.07)	− 0.63 (− 0.70, − 0.53)	28.66 (22.23, 35.03)	− 0.66 (− 0.73, − 0.56)	4.22 (1.04, 9.67)	15.05 (4.44, 39.10)	0.09 (0.03, 0.18)	0.34 (− 0.17, 1.09)
Southern sub-Saharan Africa	60.44 (53.28, 69.99)	− 0.26 (− 0.44, − 0.10)	55.85 (46.97, 65.85)	− 0.31 (− 0.48, − 0.15)	4.53 (1.65, 10.15)	5.40 (0.86, 26.35)	0.06 (0.02, 0.15)	− 0.09 (− 0.59, 1.05)
Western sub-Saharan Africa	45.01 (36.28, 54.17)	− 0.58 (− 0.68, − 0.42)	41.90 (33.04, 51.36)	− 0.60 (− 0.69, − 0.46)	3.07 (1.07, 6.62)	4.09 (1.15, 12.49)	0.04 (0.01, 0.09)	− 0.05 (− 0.48, 0.76)
High-middle SDI	2.07 (1.85, 2.37)	− 0.79 (− 0.83, − 0.72)	1.71 (1.43, 2.04)	− 0.82 (− 0.86, − 0.76)	0.27 (0.15, 0.46)	− 0.23 (− 0.70, 1.43)	0.09 (0.05, 0.15)	− 0.54 (− 0.67, − 0.37)
High SDI	0.60 (0.54, 0.68)	− 0.80 (− 0.82, − 0.77)	0.58 (0.51, 0.64)	− 0.81 (− 0.83, − 0.78)	0.02 (0.01, 0.05)	− 0.65 (− 0.82, − 0.33)	0.01 (0.00, 0.01)	− 0.23 (− 0.48, 0.26)
Low-middle SDI	33.81 (29.83, 38.66)	− 0.67 (− 0.73, − 0.56)	30.15 (24.74, 34.81)	− 0.71 (− 0.77, − 0.61)	3.45 (1.07, 7.70)	13.92 (3.62, 43.57)	0.21 (0.07, 0.46)	0.09 (− 0.43, 0.96)
Low SDI	60.16 (52.67, 70.84)	− 0.61 (− 0.67, − 0.50)	54.91 (47.18, 64.86)	− 0.65 (− 0.71, − 0.55)	5.09 (2.05, 10.44)	7.74 (3.27, 17.00)	0.16 (0.05, 0.36)	0.18 (− 0.40, 1.52)
Middle SDI	9.85 (8.81, 11.63)	− 0.74 (− 0.78, − 0.64)	8.98 (7.79, 10.68)	− 0.76 (− 0.80, − 0.67)	0.78 (0.28, 1.58)	0.06 (− 0.64, 2.40)	0.08 (0.03, 0.16)	− 0.24 (− 0.53, 0.13)

Globally, the World Health Organization began to recommend the XDR-TB surveillance in 1991. Consequently, the ASMR of XDR-TB has been tracked and reported since 1993. However, the GBD 2021 database provides total percentage change data for the periods 1990–2000, 2000–2021, 1990–2021, 2010–2021, and 2019–2021. Therefore, percentage change of ASMR for XDR-TB spanning 2010–2021 were used in the study

*ASMR* Age-standardized mortality rates, *DS-TB* drug-susceptible tuberculosis, *GBD* Global Burden of Disease, *HIV* human immunodeficiency virus, *MDR-TB* multidrug-resistant tuberculosis without extensive drug resistance, *SDI* Sociodemographic Index, *TB* Tuberculosis, *UI* Uncertainty interval, *XDR-TB* extensively drug-resistant tuberculosis

In 2021, TB resulted in 1.16 million deaths (95% UI: 1.05, 1.31 million), DS-TB accounted for 1.05 million deaths (95% UI: 0.91, 1.20 million), MDR-TB caused 0.11 million deaths (95% UI: 0.04, 0.21 million), and XDR-TB led to 7946 deaths (95% UI: 3326, 14,859 persons. Additional file 1: Table S4).

In 2021, the ASMR for TB in males was 18.19 per 100,000 population (95% UI: 16.16, 21.80 per 100,000 population), reflecting a decline of − 0.65% (95% UI: − 0.72, − 0.47%) compared to 2021. In females, the ASMR was 10.22 per 100,000 population (95% UI: 9.28, 11.33 per 100,000 population), with a decline of – 0.66% (95% UI: − 0.71, − 0.62%) compared to 2021. ASMR for TB, DS-TB were higher in males than in females (all *P* < 0.05), whereas the ASMR for MDR-TB and XDR-TB showed no significant differences between genders (all *P* > 0.05). Compared to 1990, the ASMR for TB and DS-TB generally declined in both males and females by 2021. However, the ASMR for MDR-TB increased in both genders in 2021 (Table 2).

In 2021, the ASMR for TB, DS-TB, and MDR-TB were highest in low SDI regions, with TB and DS-TB rates significantly exceeding those in other regions. The ASMR for XDR-TB was highest in low-middle SDI countries. Over the past 30 years, the ASMR for TB and DS-TB declined across varying SDI categories (Additional file 1: Table S2). For MDR-TB, declines in ASMR were also noted in high SDI regions (EAPC = − 6.13, 95% *CI: *− 6.71, − 5.08), high-middle SDI regions (EAPC = − 3.67, 95% *CI:* − 5.42, − 1.88), and middle SDI regions (EAPC = − 2.51, 95% *CI: *− 3.70, − 1.31). In contrast, increases in ASMR were observed in low-middle SDI regions (EAPC = 4.80, 95% *CI: *2.19, 7.48) and low SDI regions (EAPC = 3.61, 95% *CI:* 1.49, 5.77). For XDR-TB, the ASMR exhibited significant increase in high-middle SDI regions (EAPC = 4.53, 95% *CI: *1.36, 7.60), middle SDI regions (EAPC = 3.50, 95% *CI:* 1.37, 5.68), low-middle SDI regions (EAPC = 9.78, 95% *CI:* 6.57, 13.09), and low SDI regions (EAPC = 10.88, 95% *CI:* 7.65, 14.19. Additional file 1: Table S2).

In 2021, the overall ASMR due to TB was highest in Central sub-Saharan Africa and lowest in high-income North America. The ASMR for DS-TB was also highest in Central sub-Saharan Africa and lowest in Western Europe. The ASMR for MDR-TB peaked in South Asia and was lowest in high-income North America, while the highest ASMR for XDR-TB was observed in Central Asia.

Comparing 2021 to 1990, the ASMR of TB and DS-TB decreased across all 21 global geographical regions, with the most rapid declines observed in East Asia and the slowest in Southern sub-Saharan Africa. However, the ASMR MDR-TB increased in Oceania, Central Asia, South Asia, Eastern Europe, Tropical Latin America, Eastern sub-Saharan Africa, Southern sub-Saharan Africa, and Western sub-Saharan Africa (all *P* < 0.05), while it decreased in Western Europe and high-income North America (all *P* < 0.05) (Table 2).

In 2021, the highest ASMR for TB was recorded in the Central African Republic, which also had the highest ASMR for DS-TB. The highest ASMR for MDR-TB was observed in Somalia, while Mongolia had the highest ASMR for XDR-TB (Additional file 1: Table S3). Compared to 1990, the ASMR for TB in 2021 did not significantly increase in most countries and regions, with many showing a declining trend (all *P* < 0.05. Additional file 1: Table S3). However, the decline was slowest in Lesotho. The ASMR for DS-TB decreased in many countries but increased in several countries and territories, with notable rises in Zimbabwe. The highest increases in the ASMR for MDR-TB were observed in Somalia (*P* < 0.05. Additional file 1: Table S3).

### DALY and temporal trend

In 2021, the global age-standardized DALY rate for all TB was estimated at 580.26 per 100,000 population (95% UI: 522.37, 649.82 per 100,000 population), with a declining by − 0.65% (95% UI: − 0.69, − 0.57%) compared to 1990. For DS-TB, the age-standardized DALY rate was 526.03 per 100,000 population (95% UI: 457.25, 596.46 per 100,000 population), declining by – 0.68% (95% UI: − 0.72, − 0.60%) compared to 1990. MDR-TB had an age-standardized DALY rate of 50.76 per 100,000 population (95% UI: 21.28, 99.37 per 100,000 population), with an increase of 2.68% (95% UI: 0.53, 7.70%) compared to 1990. XDR-TB showed an age-standardized DALY rate of 0.58 per 100,000 population (95% UI: 0.19, 1.39 per 100,000 population), with a percentage change of − 0.25% (95% UI: − 0.47, 0.05%) compared to 1990. The EAPC of the age-standardized DALY rate of TB, DS-TB, and MDR-TB were -3.50 (95% *CI:* − 3.67, − 3.32), − 3.75 (95% *CI: *− 3.87, − 3.62), and 1.36 (95% *CI: *− 0.30, 3.04) from 1990 to 2021, respectively.

In 2021, TB caused a total of 46.98 million DALYs (95% UI: 42.48, 52.46 million), with DS-TB contributing 42.56 million DALYs (95% UI: 36.84, 48.24 million), MDR-TB contributing 4.13 million DALYs (95% UI: 1.71, 8.06 million), and XDR-TB contributing 0.29 million DALYs (95% UI: 0.12, 0.53 million. Additional file 1: Table S5).

In 2021, the age-standardized DALY rate of all HIV-negative TB in males was 705.23 per 100,000 population (95% UI: 623.40, 834.06 per 100,000 population), with a decline of -0.63% (95% UI: − 0.69, − 0.47%) compared to 1990 (Table 3). For females, the age-standardized DALY rate for all HIV-negative TB was 463.18 per 100,000 population (95% UI: 417.49, 512.9 per 100,000 population), with a decline of − 0.67% (95% UI*:* − 0.71, − 0.63%) compared to 1990 (Table 3). In 2021, the age-standardized DALY rate caused by TB was higher in males compared to females (*P* < 0.05). Specifically, the age-standardized DALY rate for DS-TB was higher in males than that of in females (*P* < 0.05), while the rates for MDR-TB and XDR-TB showed no significant differences between genders ( all *P* > 0.05). Compared to 1990, the age-standardized DALY rate for TB and DS-TB in both males and females showed a declining trend in 2021 (all *P *< 0.05), with no difference in the rate of decline between genders (*P* > 0.05). However, the age-standardized DALY rates for MDR-TB increased in both males and females (all *P *< 0.05), with no significant difference in the rate of increase between genders (*P* > 0.05. Table 3).

**Table 3:** Age-standardized DALY rate of TB, DS-TB, MDR-TB, and XDR-TB in HIV-negative individuals in 2021, and percentage change of age-standardized DALY rate were analyzed across GBD regions

Regions	HIV-negative TB		DS-TB		MDR-TB		XDR-TB	
	Age-standardized DALY rate (per 100,000 population) (95% UI) 2021	Percentage change of age-standardized DALY rate (95% UI) 1990–2021	Age-standardized DALY rate (per 100,000 population) (95% UI) 2021	Percentage change of age-standardized DALY rate (95% UI) 1990–2021	Age-standardized DALY rate (per 100,000 population) (95% UI) 2021	Percentage change of age-standardized DALY rate (95% UI) 1990–2021	Age-standardized DALY rate (per 100,000 population) (95% UI) 2021	Percentage change of age-standardized DALY rate (95% UI) 2010–2021
Global	580.26 (522.37, 649.82)	− 0.65 (− 0.69, − 0.57)	526.03 (457.25, 596.46)	− 0.68 (− 0.72, − 0.60)	50.76 (21.28, 99.37)	2.68 (0.53, 7.70)	0.58 (0.19, 1.39)	− 0.25 (0.47, 0.05)
Male	705.23 (623.40, 834.06)	− 0.63 (− 0.69, − 0.47)	638.98 (541.8, 752.62)	− 0.66 (− 0.72, − 0.52)	61.64 (25.73, 120.57)	2.66 (0.48, 8.29)	4.61 (2.10, 8.53)	− 0.28 (− 0.49, 0.01)
Female	463.18 (417.49, 512.9)	− 0.67 (− 0.71, − 0.63)	420.27 (367.8, 475.67)	− 0.70 (− 0.74, − 0.65)	40.52 (16.92, 78.63)	2.66 (0.54, 7.56)	2.39 (0.97, 4.50)	− 0.17 (− 0.44, 0.22)
East Asia	94.10 (79.17, 111.79)	− 0.87 (− 0.90, − 0.83)	86.21 (68.45, 105.40)	− 0.88 (− 0.91, − 0.83)	6.68 (1.81, 16.83)	− 0.83 (− 0.96, − 0.30)	2.29 (0.55, 7.85)	− 0.37 (− 0.76, 0.45)
Southeast Asia	895.71 (793.04, 1020.93)	− 0.70 (− 0.74, − 0.60)	861.88 (753.85, 987.19)	− 0.71 (− 0.75, − 0.61)	28.46 (11.07, 60.35)	1.05 (− 0.27, 4.92)	24.86 (14.07, 39.55)	− 0.28 (− 0.62, 0.28)
Oceania	1260.62 (1040.39, 1534.8)	− 0.43 (− 0.59, − 0.22)	1169.03 (928.60, 1425.23)	− 0.47 (− 0.62, − 0.27)	77.00 (17.39, 198.67)	36.35 (5.28, 215.45)	18.96 (10.51, 29.19)	2.19 (− 0.25, 9.52)
Central Asia	221.53 (196.00, 251.98)	− 0.64 (− 0.68, − 0.59)	141.35 (100.47, 179.50)	− 0.77 (− 0.83, − 0.70)	55.32 (31.87, 85.01)	26.76 (9.54, 80.51)	3.47 (1.51, 6.43)	− 0.45 (− 0.62, − 0.26)
Central Europe	38.15 (34.91, 41.41)	− 0.78 (− 0.79, − 0.76)	36.26 (32.74, 39.90)	− 0.79 (-0.81, − 0.77)	1.31 (0.44, 2.88)	− 0.35 (− 0.79, 0.95)	0.36 (0.07, 1.22)	− 0.40 (− 0.80, 0.62)
Eastern Europe	133.78 (121.20, 147.77)	− 0.46 (− 0.51, − 0.41)	72.33 (46.72, 101.88)	− 0.70 (− 0.81, − 0.59)	42.49 (24.80, 59.65)	5.92 (1.77, 17.53)	0.03 (0.01, 0.09)	− 0.52 (− 0.67, − 0.29)
High-income Asia Pacific	23.17 (20.77, 25.66)	− 0.89 (− 0.90, − 0.87)	22.61 (20.18, 25.10)	− 0.89 (− 0.90, − 0.87)	0.45 (0.10, 1.45)	− 0.82 (− 0.97, -0.20)	0.75 (0.21, 2.16)	− 0.31 (− 0.78, 1.08)
Australasia	4.25 (3.75, 4.81)	− 0.74 (− 0.76, − 0.71)	3.98 (3.43, 4.53)	− 0.75 (− 0.78, − 0.72)	0.22 (0.07, 0.55)	0.21 (− 0.69, 4.15)	5.20 (1.84, 11.92)	0.65 (− 0.52, 4.28)
Western Europe	7.35 (6.71, 8.02)	− 0.82 (− 0.83, − 0.81)	6.99 (6.33, 7.66)	− 0.83 (− 0.84, − 0.82)	0.29 (0.14, 0.55)	− 0.54 (− 0.74, − 0.09)	14.59 (3.29, 39.97)	− 0.12 (− 0.41, 0.39)
Southern Latin America	52.94 (49.39, 56.77)	− 0.76 (− 0.78, − 0.74)	51.39 (47.02, 55.65)	− 0.77 (− 0.79, − 0.75)	1.23 (0.26, 3.76)	− 0.17 (− 0.83, 2.62)	1.21 (0.30, 3.13)	− 0.04 (− 0.72, 1.37)
High-income North America	4.99 (4.62, 5.39)	− 0.77 (− 0.78, − 0.76)	4.83 (4.35, 5.22)	− 0.77 (− 0.78, − 0.75)	0.13 (0.04, 0.37)	− 0.90 (− 0.96, − 0.67)	5.36 (1.92, 12.43)	0.53 (− 0.38, 2.91)
Caribbean	247.18 (168.62, 534.96)	− 0.60 (− 0.71, − 0.45)	244.67 (167.68, 529.26)	− 0.61 (− 0.71, − 0.45)	2.15 (0.40, 6.66)	− 0.49 (− 0.90, 1.09)	2.6 (1.01, 5.36)	0.74 (− 0.49, 0.98)
Andean Latin America	262.27 (213.74, 326.15)	− 0.88 (− 0.90, − 0.84)	225.48 (171.63, 284.34)	− 0.89 (− 0.92, − 0.86)	31.59 (11.9, 68.16)	− 0.04 (− 0.66, 2.22)	1.23 (0.39, 2.75)	− 0.04 (− 0.50, 0.82)
Central Latin America	88.38 (78.7, 100.08)	− 0.81 (− 0.83, − 0.78)	82.86 (72.36, 94.81)	− 0.82 (− 0.84, − 0.80)	4.74 (1.64, 10.35)	3.06 (0.47, 10.01)	0.05 (0.01, 0.13)	0.20 (− 0.38, 1.28)
Tropical Latin America	91.92 (87.08, 97.23)	− 0.72 (− 0.73, − 0.71)	85.98 (73.30, 93.89)	− 0.74 (− 0.78, − 0.72)	5.10 (1.02, 14.91)	14.01 (1.20, 114.97)	0.07 (0.03, 0.15)	0.42 (− 0.60, 2.71)
North Africa and Middle East	154.48 (124.84, 212.96)	− 0.75 (− 0.80, − 0.66)	143.76 (110.69, 199.33)	− 0.77 (− 0.82, − 0.69)	9.97 (3.13, 25.00)	2.64 (0.24, 10.11)	0.78 (0.27, 1.80)	− 0.28 (− 0.70, 0.76)
South Asia	1133.21 (1015.74, 1304.29)	− 0.72 (− 0.76, − 0.64)	986.71 (766.48, 1170.40)	− 0.75 (− 0.81, − 0.68)	139.31 (35.46, 313.45)	16.45 (2.36, 84.88)	7.19 (1.79, 16.99)	0.12 (− 0.57, 1.35)
Central sub-Saharan Africa	3530.2 (2569.58, 4965.79)	− 0.52 (− 0.63, − 0.36)	3361.35 (2444.44, 4756.21)	− 0.54 (− 0.65, − 0.39)	166.56 (42.73, 566.43)	2.80 (− 0.23, 19.09)	0.32 (0.06, 1.07)	0.11 (− 0.62, 2.31)
Eastern sub-Saharan Africa	2538.08 (2096.61, 3066.18)	− 0.67 (− 0.72, − 0.59)	2345.73 (1904.02, 2864.53)	− 0.69 (− 0.75, − 0.61)	189.74 (75.5, 390.11)	13.44 (3.95, 34.66)	2.35 (0.80, 5.57)	0.28 (− 0.22, 0.99)
Southern sub-Saharan Africa	2369.82 (2080.46, 2768.09)	− 0.33 (− 0.48, − 0.19)	2195.17 (1851.87, 2601.57)	− 0.37 (− 0.52, − 0.23)	172.30 (63.88, 382.47)	4.83 (0.67, 23.78)	0.84 (0.14, 2.58)	− 0.08 (− 0.57, 1.07)
Western sub-Saharan Africa	1401.6 (1096.23, 1729.1)	− 0.62 (− 0.71, − 0.49)	1310.34 (1022.21, 1628.52)	− 0.64 (− 0.72, − 0.52)	90.03 (31.43, 197.30)	3.41 (0.84, 10.92)	0.11 (0.02, 0.35)	− 0.10 (− 0.52, 0.70)
High-middle SDI	87.95 (79.10, 100.05)	− 0.78 (− 0.81, − 0.72)	73.19 (61.90, 85.61)	− 0.81 (− 0.84, − 0.75)	11.04 (6.33, 18.03)	− 0.14 (− 0.67, 1.72)	3.72 (2.06, 5.94)	− 0.57 (− 0.68, -0.41)
High SDI	17.78 (15.97, 20.38)	− 0.82 (− 0.84, − 0.79)	16.88 (14.94, 19.28)	− 0.82 (− 0.84, − 0.79)	0.74 (0.31, 1.52)	− 0.65 (− 0.83, − 0.29)	0.15 (0.07, 0.30)	− 0.32 (− 0.53, 0.01)
Low-middle SDI	1154.27 (1020.37, 1300.64)	− 0.69 (− 0.73, − 0.60)	1033.52 (854.57, 1189.41)	− 0.72 (− 0.78, − 0.64)	113.99 (37.12, 248.83)	12.94 (3.29, 40.89)	6.76 (2.26, 14.63)	0.05 (− 0.44, 0.90)
Low SDI	1976.06 (1708.98, 2309.04)	− 0.65 (− 0.70, − 0.56)	1813.84 (1550.67, 2114.12)	− 0.68 (− 0.73, − 0.59)	157.61 (66.11, 316.55)	6.79 (2.74, 14.88)	4.60 (1.59, 10.13)	0.07 (− 0.44, 1.18)
Middle SDI	368.03 (332.81, 427.21)	− 0.73 (− 0.76, − 0.65)	337.28 (296.19, 391.47)	− 0.74 (− 0.78, − 0.67)	27.94 (10.72, 55.83)	0.11 (− 0.62, 2.65)	2.82 (1.18, 5.16)	− 0.23 (− 0.52, 0.14)

Globally, the World Health Organization began to recommend the XDR-TB surveillance in 1991. Consequently, the age-standardized DALY rate of XDR-TB has been tracked and reported since 1991. However, the GBD 2021 database provides total percentage change data for the periods 1990–2000, 2000–2021, 1990–2021, 2010–2021, and 2019–2021. Therefore, percentage change of age-standardized DALY rates for XDR-TB spanning 2010–2021 were used in the study

*DALYs* disability-adjusted life years, *DS-TB* drug-susceptible tuberculosis, *GBD* Global Burden of Disease, *HIV* human immunodeficiency virus, *MDR-TB* multidrug-resistant tuberculosis without extensive drug resistance, *SDI* Sociodemographic Index, *TB* Tuberculosis, *UI* Uncertainty interval, *XDR-TB* extensively drug-resistant tuberculosis

In 2021, the age-standardized DALY rate of TB, DS-TB, and MDR-TB was highest in regions with low SDI, followed by low-middle SDI regions. The age-standardized DALY rates of TB and DS-TB in Low SDI areas were significantly higher than in other regions. The age-standardized DALY rate of XDR-TB was highest in low-middle SDI region, followed by low SDI region. Over the past 30 years, the age-standardized DALY rate for TB and DS-TB showed a significant decline across five SDI regions (all *P* < 0.05. Additional file 1: Table S2). For MDR-TB, the age-standardized DALY rate declined in high SDI regions (EAPC = – 5.98, 95% *CI:* − 6.99, − 4.96), high-middle SDI regions (EAPC = − 3.30, 95% *CI:* − 5.15, − 1.40), and middle SDI regions (EAPC = − 2.34, 95% *CI:* − 5.15, − 1.40). However, increases were observed in low-middle SDI regions (EAPC = 4.60, 95% *CI: *2.02, 7.24) and low SDI regions (EAPC = 3.25, 95% *CI: *1.13, 5.42). XDR-TB experienced an increase in the age-standardized DALY rate across five SDI regions, regions with lower SDI values showed the greatest increases in age-standardized DALY rates. For instance, low SDI regions had the highest increase (EAPC = 15.30, 95% *CI:* 10.90, 19.67) in in age-standardized DALY rates (Additional file 1: Table S2).

In 2021, the age-standardized DALY rates for TB, DS-TB, and MDR-TB were highest in Central sub-Saharan Africa. The lowest age-standardized DALY rates for TB and DS-TB were observed in Australasia, while the lowest rates for MDR-TB were in high-income North America. The highest age-standardized DALY rates for XDR-TB were found in Southeast Asia. Compared to 1990, the age-standardized DALY rates for TB and DS-TB decreased across all 21 global regions by 2021, with the largest declines observed in the high-income Asia Pacific (all *P* < 0.05. Table 3). Trends for MDR-TB varied, with the highest increases in age-standardized DALY rates observed in Oceania (*P* < 0.05. Table 3), and most significant decreases in high-income North America (*P* < 0.05. Table 3).

In 2021, the Central African Republic had the highest age-standardized DALY rates for TB and DS-TB. Somalia recorded the highest age-standardized DALY rates for MDR-TB, while Mongolia had the highest rates for XDR-TB. Compared to 1990, the age-standardized DALY rates for TB in 2021 did not significantly increase globally (*P* > 0.05), with many countries showing a declining trend. However, the decrease in Lesotho is the smallest. The age-standardized DALY rate for DS-TB increased in Zimbabwe but decreased in other regions. The fastest increases in age-standardized DALY rates for MDR-TB were observed in Somalia (Additional file 1: Table S3).

### Age-gender characteristics

**Fig. 1:**
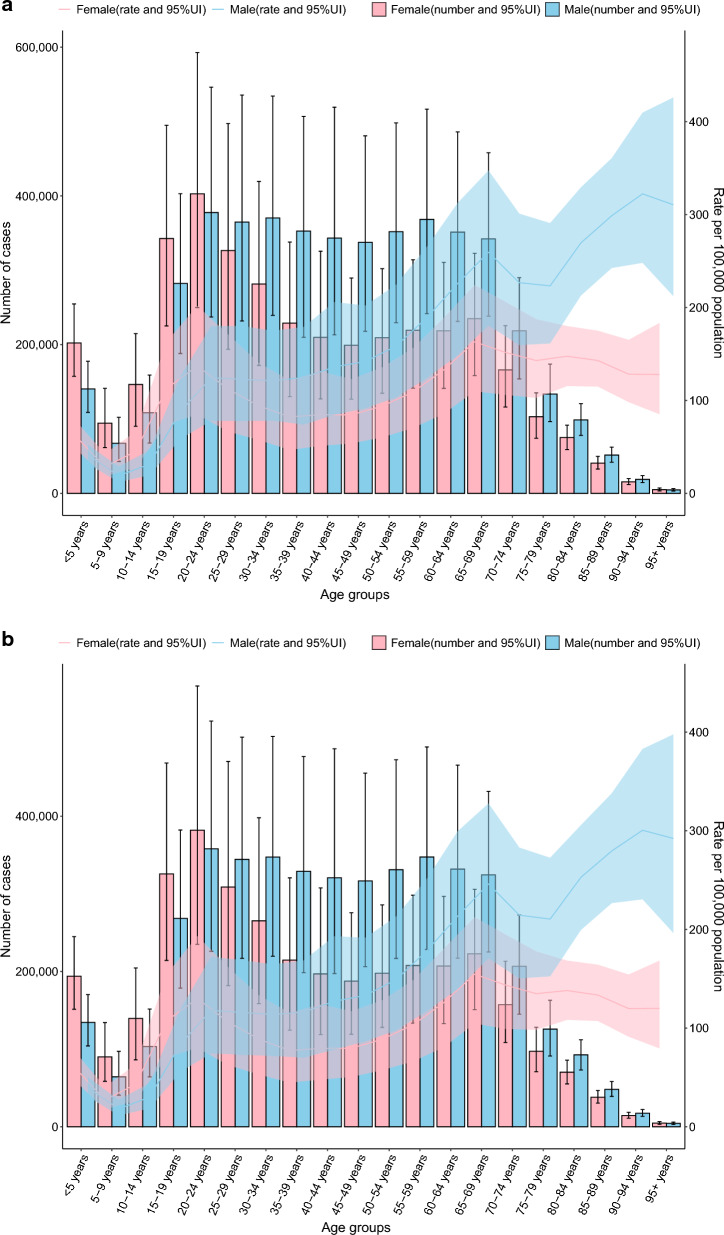

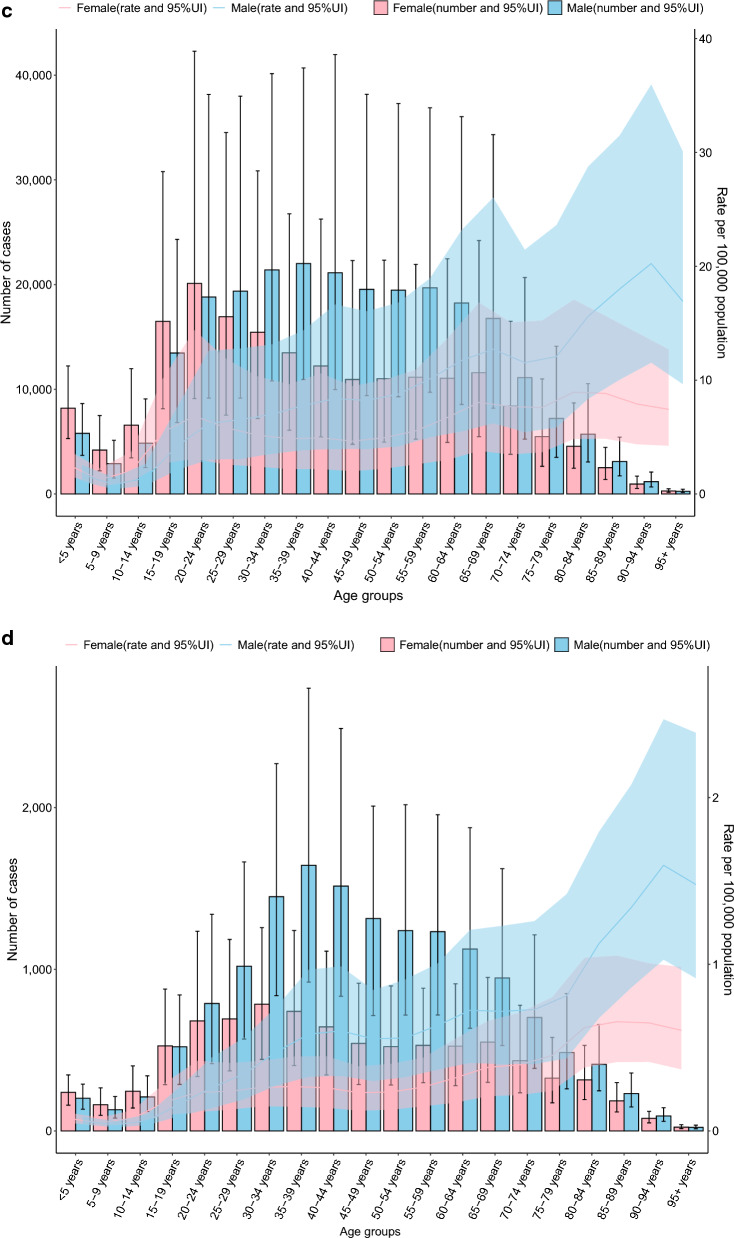
The specific incidence of TB, DS-TB, MDR-TB, and XDR-TB showed notable differences across age and gender distribution in 2021 year (**A** Incidence of TB, **B** Incidence of DS-TB. **C** Incidence of MDR-TB. **D** Incidence of XDR-TB. *DS-TB* drug-susceptible tuberculosis, *MDR-TB* multidrug-resistant tuberculosis without extensive drug resistance, *TB* Tuberculosis, *XDR-TB* extensively drug-resistant tuberculosis)

In the 80–84 age group and above, the specific incidence rates of TB and DS-TB are higher in males than in females (*P *< 0.05), with no significant differences observed in other age groups (all *P* > 0.05). The specific incidence rates for MDR-TB and XDR-TB show no differences between males and females across all age groups (all *P* > 0.05. Fig. 1 A-D).

In the 30–34 age group and above, the specific mortality rate of TB is higher in males than in females (*P *< 0.05), with no significant differences in other age groups (all *P* > 0.05). In the 25–29 age group and those aged 30 and above, the specific mortality rate of DS-TB is higher in males than in females (all *P *< 0.05). The specific mortality rates for MDR-TB and XDR-TB show no differences between males and females across all age groups (all *P* > 0.05. Additional file 1: Fig.S1 A-D).

In the 25–29 age group and above, the specific DALY rates for TB and DS-TB are higher in males than in females (all *P *< 0.05), with no significant differences in other age groups (all *P* > 0.05). The specific DALY rates for MDR-TB and XDR-TB show no differences between males and females across all age groups (all *P* > 0.05. Additional file 1: Fig.S2 A-D).

### Association between ASRs and SDI

In 2021, across 204 countries and territories, the ASIR, ASMR, and age-standardised DALY rate for TB exhibited a significant negative correlation with the SDI, demonstrating a rapid decline as SDI increased (ASIR: *r* = − 0.799, *P* < 0.001; ASMR: *r* = − 0.857, *P* < 0.001; DALYs: *r* = − 0.862, *P* < 0.001. Respectively. Additional file 1: Fig. S3 A–C). Similarly, for DS-TB, a comparable trend was observed, with substantial decreases in ASIR (*r* = − 0.804, *P* < 0.001), ASMR (*r* = − 0.859, *P* < 0.001), and age-standardized DALY rates (*r* = − 0.866, *P* < 0.001) accompanying increases in SDI (Additional file 1: Fig. S4A–C). In addition, a pronounced negative correlation was observed between the ASIR, ASMR, and age-standardised DALY rate for MDR-TB and SDI (ASIR: *r* = − 0.642, *P* < 0.001; ASMR: *r* = − 0.782, *P* < 0.001; DALYs: *r* = − 0.788, *P* < 0.001. Respectively. Additional file 1: Fig. S5 A-C). For XDR-TB, significant inverse associations were also noted with SDI, as evidenced by the trends in ASIR (*r* = − 0.485, *P* < 0.001), ASMR (*r* = − 0.611, *P* < 0.001), and age-standardized DALY rates (*r* = − 0.625, *P* < 0.001. Additional file 1: Fig. S6 A–C).

From 1990 to 2021, the ASIR (*r* = − 0.865, *P* < 0.001), ASMR (*r* = − 0.872, *P* < 0.001), and age-standardized DALY rate (*r* = − 0.865, *P* < 0.001) for TB declined rapidly with increases in the SDI globally. However, these trends varied across different GBD regions. TB incidence rates modestly increased with rising SDI in Central and Southern sub-Saharan Africa, as well as Southeast Asia. Conversely, TB mortality and age-standardized DALY rate showed rapid declines in Central sub-Saharan Africa and South Asia as SDI increased (Additional file 1: Fig.S7 A–C). The trends in ASIR (*r* = − 0.813, *P* < 0.001), ASMR (*r* = − 0.886, *P* < 0.001), and age-standardized DALY rate (*r* = − 0.879, *P* < 0.001) for DS-TB mirrored those of TB (Additional file 1: Fig. S8 A–C). For MDR-TB, the ASIR (*r* = − 0.504, *P* < 0.001), ASMR (*r* = − 0.686, *P* < 0.001), and age-standardized DALY rate (*r* = − 0.674, *P* < 0.001) decreased slowly with rising SDI. However, the ASIR of MDR-TB in South Asia showed a continuous increase with rising SDI. The ASIR, ASMR, and DALY rates for MDR-TB initially rose sharply with increasing SDI, peaked, and then declined rapidly as SDI continued to rise in East Asia, Eastern Europe, and Central sub-Saharan Africa (Additional file 1: Fig. S9 A–C). The ASIR and ASMR of XDR-TB showed a rapid upward trend with increasing SDI in Central sub-Saharan Africa and South Asia. In Eastern Europe, Central Asia, and East Asia, the ASMR and age-standardized DALY rate for XDR-TB rose swiftly with increasing SDI, reached a peak, and then declined sharply with further increases in SDI (Additional file 1: Fig. S10 A–C).

### Risk factors for ASMRs and age-standardized DALY rates

**Fig. 2:**
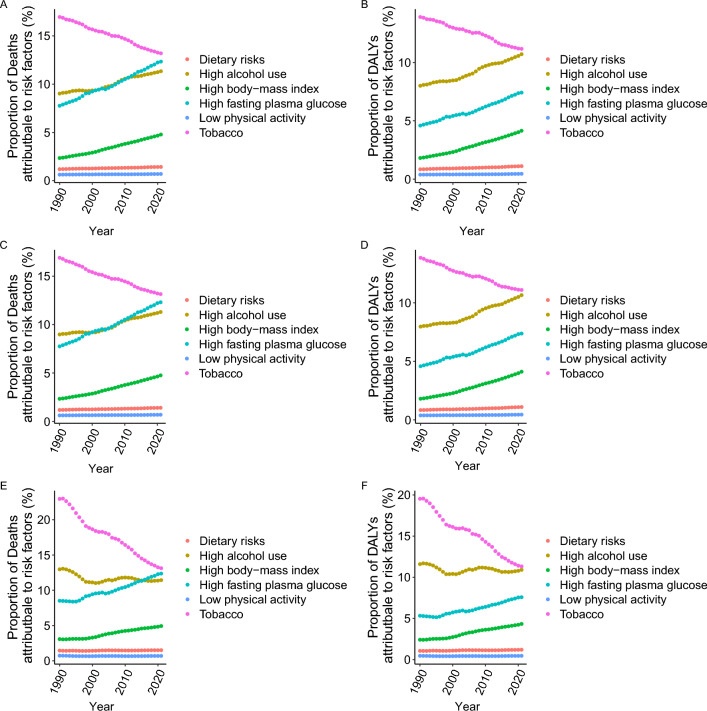
The association between risk factors and the age-standardized mortality rate and DALY rate of TB, DS-TB, and MDR-TB in 21 GBD regions from 1990 to 2021 (**A** mortality rate of TB. **B** DALY rate of TB. **C** mortality rate of DS-TB. **D** DALY rate of DS-TB. **E** mortality rate of MDR-TB. **F** DALY rate of MDR-TB. *DALYs* disability-adjusted life years, *DS-TB* drug-susceptible tuberculosis, *GBD* Global Burden of Disease, *MDR-TB* multidrug-resistant tuberculosis without extensive drug resistance, *TB* Tuberculosis)

From 1990 to 2021, the major risk factors contributing to the ASMR and age-standardized DALY rate for TB were ranked as follows: tobacco use, high fasting plasma glucose, high body mass index, dietary risks, and low physical activity. Notably, the proportion attributed to tobacco use has been steadily declining, whereas the proportions due to high fasting plasma glucose and high body mass index have been increasing (Fig. 2 A, B). The risk factors and trends for DS-TB and MDR-TB mirrored those of TB (Fig. 2 C–F). For XDR-TB over the same period, the risk factors for ASMR and age-standardized DALY rate were similarly ranked (Additional file 1: Fig. S11 A, B). However, the contribution of tobacco to the ASMR and DALY rates for XDR-TB initially rose gradually from 1990 to 2005 and then declined rapidly thereafter. Meanwhile, the proportions due to high fasting plasma glucose and high body mass index have continued to rise (Additional file 1: Fig. S11 A, B).

The contributions of dietary risks and low physical activity to the ASMR and age-standardized DALY rates for TB, DS-TB, MDR-TB, and XDR-TB have remained relatively unchanged and consistently low (Fig. 2 A–F. Additional file 1: Fig. S11 A, B).

### Projecting disease burden

By 2035, the projected ASIR for TB is 76.76 per 100,000 population (95% *CI:* 69.61, 83.99 per 100,000 population), and the ASMR is 8.70 per 100,000 population (95% *CI:* 7.69, 9.70 per 100,000 population). Between 2022 and 2035, its projections indicate declining trends for DS-TB, while MDR-TB and XDR-TB are expected to see significant increases in both ASIR and ASMR, highlighting the growing challenge of drug-resistant TB (Table 4. Additional file 1: Table S6).

**Table 4:** Predicted age-standardized rates for TB, DS-TB, MDR-TB, and XDR-TB from 2022 to 2035, based on the Bayesian age-period-cohort model

Index	HIV-negative TB		DS-TB		MDR-TB		XDR-TB	
	ASR (per 100,000 population) (95% *CI*) 2035	EAPC (95% *CI*) 2022–2035	ASR (per 100,000 population) (95% *CI*) 2035	EAPC (95% *CI*) 2022–2035	ASR (per 100,000 population) (95% *CI*) 2035	EAPC (95% *CI)* 2022–2035	ASR (per 100,000 population) (95% *CI*) 2035	EAPC (95% *CI*) 2022–2035
Incidence	76.75 (69.51, 83.99)	− 1.97 (− 1.98, 1.95)	70.76 (64.96, 76.55)	− 2.13 (− 2.14, − 2.12)	11.02 (0.00, 23.99)	5.18 (5.16, 5.20)	3.91 (0.00, 24.04)	20.34 (20.32, 20.36)
Death	8.70 (7.69, 9.70)	− 3.43 (− 3.43, 3.42)	7.46 (6.75, 8.16)	− 3.75 (− 3.75, − 3.74)	2.45 (0.00, 5.11)	4.49 (4.49, 4.50)	1.38 (0.00, 10.50)	21.03 (21.01, 21.05)

Globally, the World Health Organization began to recommend XDR-TB surveillance in 1991. Consequently, the age-standardized incidence rate of XDR-TB has been tracked and reported since 1991, and the age-standardized mortality rate has been tracked and reported since 1993. When predicting ASRs for a given year, if the lower limit of the 95% confidence interval is below 0, it is set to 0

*ASR* age-standardized rate, *CI* Confidence interval, *DS-TB* drug-susceptible tuberculosis, *EAPC* estimated annual percentage change, *HIV* human immunodefciency virus, *MDR-TB* multidrug-resistant tuberculosis without extensive drug resistance, *TB* Tuberculosis, *XDR-TB* extensively drug-resistant tuberculosis

## Discussion

The study is the first to use the GBD 2021 database to assess the burden and trends of HIV-negative TB over the past 30 years. Significant differences in ASIR, ASMR, and age-standardized DALY rates for HIV-negative TB, DS-TB, MDR-TB, and XDR-TB were observed across different countries and regions. The ASIR, ASMR, and age-standardized DALY rate for HIV-negative TB have all decreased from 1990 to 2021, indicating a global decline in the overall burden. However, TB remains a persistent threat in sub-Saharan Africa, Southeast Asia, and Eastern Europe, particularly in low SDI regions. In addition, the ASIR and ASMR of MDR-TB and XDR-TB have increased in recent years, highlighting drug-resistant TB as a severe global public health issue. These findings provide essential technical support and decision-making evidence for governments worldwide to formulate key TB control measures and to plan and allocate health resources effectively. These findings supports the development of national health plans, rational allocation of medical institutions, human resources, equipment, and funding, and the concentration of resources on priority health issues to achieve greater cost-effectiveness and social impact.

### Efficient and innovative diagnostic technologies and strategies are needed to control the spread of TB

Significant progress has been made in global TB control over the past decades, aligned with the SDGs to end the TB epidemic. However, the decline in TB incidence remains disappointing, with one in three TB patients undiagnosed and many not receiving timely diagnosis and appropriate treatment [22]. In high-burden countries, case detection and treatment success rates are still alarmingly low [1, 2]. Of greater concern is the rising incidence and mortality of MDR-TB and XDR-TB globally, driven primarily by transmission between individuals rather than by the mutation of DS-TB strains due to inadequate treatment [23]. Early detection and standardized treatment of new TB cases, particularly MDR-TB and XDR-TB, are crucial for accelerating recovery and curbing the community transmission of drug-resistant TB [24].

Effective TB control requires significant breakthroughs across various fronts. Key areas include developing highly sensitive and specific rapid diagnostic tools, creating more effective drugs for both drug-susceptible and drug-resistant TB strains, and innovating more effective vaccines [2]. Governments must provide substantial funding, technology, and healthcare services to transition from traditional Directly Observed Treatment, Short-Course (DOTS) passive case detection to proactive case identification in high-burden areas [2]. Comprehensive health education, care, and medication should be provided to the most vulnerable populations, integrated with other health services, particularly HIV/AIDS services, to deliver efficient and holistic healthcare. Ensuring adequate health surveillance at primary healthcare and community health service institutions is also crucial [25, 26].

To effectively control TB, early and accurate case detection, prompt initiation, and adherence to effective treatment are crucial for breaking the transmission chain [27]. In high-burden countries, many TB patients are asymptomatic, and treating infections only after symptoms appear is insufficient to significantly reduce community transmission and incidence rates [2]. Proactive strategies are needed to address health system barriers to TB control, including routine screening of household contacts of TB patients and shifting from empirical detection based on clinical symptoms to active case detection through sputum smear and culture [28].

Advances in diagnostic technology have made early TB diagnosis more feasible. Tools like the molecular diagnostic GeneXpert *MTB*/RIF reduce TB diagnosis time from 1–2 weeks to a few hours [29]. Second-generation sequencing technology is also time-efficient, providing diagnostic directions in cases of co-infection with rare or multiple pathogens [30]. The emergence of novel biomarkers, specifically TB-specific host biomarkers and *Mtb* biomarkers, allows the development of evaluation models that enable rapid, accurate, and effective monitoring of *Mtb* infection and TB treatment efficacy [31–33]. Future efforts must focus on developing affordable, accessible, highly sensitive, and specific screening methods and indicators. Continuously optimizing screening strategies and proactively identifying TB-infected patients are essential to effectively control the spread of the TB epidemic.

### Effective TB control requires low-toxicity anti-TB drugs, effective vaccines, and an efficient primary healthcare system

The study confirms previous findings [13], indicating that the TB burden is more severe among males than females, with TB incidence rates being 50% higher and mortality rates 100% higher in males in many countries. Across all levels of sociodemographic development, the TB mortality rate for HIV-negative males over 30 years of age consistently surpasses that for HIV-negative females. This underscores the importance of considering gender factors in TB epidemiology and calls for gender-sensitive public health interventions.

The study found that all ASRs of HIV-negative TB were inversely related to the SDI, with high-SDI regions exhibiting low ASIR, ASMR, and age-standardized DALY rates for DS-TB, MDR-TB, and XDR-TB. Conversely, the ASIR, ASMR, and age-standardized DALY rates for MDR-TB and XDR-TB remain high in low-SDI regions, particularly in Central sub-Saharan Africa and South Asia, where these rates have been persistently rising. This highlights the ongoing public health challenge in these areas and the need for targeted interventions.

The WHO-recommended strategies for TB control have evolved significantly over time. Initially, the TB control strategy was clinical and programmatic, focusing mainly on providing standardized regimens and medications [34]. The underlying assumption was that existing biomedical tools could primarily solve TB transmission, with the premise that curing patients with active disease would reduce mortality, lower disease prevalence, decrease transmission, and subsequently reduce incidence [35]. However, the actual situation in many countries is more complex, health systems are inefficiently managed, financially under-resourced, and severely understaffed in many LMICs. Additionally, there is a lack of adequate drug production capacity and low levels of health system informatization [36]. These issues significantly hinder effective TB control and have not been adequately addressed in TB control efforts.

There is an urgent need for implementation research to evaluate the behavioral factors and conditions affecting the efficacy of drug treatments for TB. Such studies are essential to modify and improve TB control strategies, enhancing the real-world effectiveness of anti-TB medications, improving patient outcomes, reducing mortality rates, shortening the duration of bacterial shedding, and decreasing the risk of community transmission and overall incidence [2, 37]. Chemotherapy remains the most crucial treatment for drug-resistant TB [37]. However, challenges such as prolonged treatment duration, poor clinical efficacy, numerous adverse effects, and high mortality rates persist. Therefore, developing new drugs and optimizing chemotherapy regimens are vital to increasing cure and survival rates [38]. These advancements are crucial for the clinical treatment and control of TB.

Currently, the only licensed vaccine for TB prevention is the bacille Calmette-Guérin (BCG) vaccine. Developed nearly a century ago, BCG is effective in preventing severe forms of TB in children, essentially mitigating severe disease and reducing the severity of clinical symptoms in pediatric TB cases. This vaccine has been widely administered to children globally for many years [2]. However, its effectiveness has shown significant geographical variability. Moreover, there is no licensed vaccine that effectively prevents TB in adults, either before or after exposure to the infection. The development and widespread administration of more effective preventive vaccines in high TB burden settings are crucial for advancing TB elimination efforts. Encouragingly, the M72/AS01E vaccine has shown promise in inducing an immune response and providing protection against the progression to pulmonary tuberculosis for at least three years [39].

### Low-SDI regions require increased focus on MDR-TB and XDR-TB

The study found that the ASIR of MDR-TB is increasing rapidly in low-SDI regions, while it is declining in high-SDI regions, consistent with previous studies [2, 40]. This disparity can be attributed to two main factors: (1) Detection Coverage. High-SDI regions have a high coverage rate for MDR-TB detection. Detected patients are promptly isolated and treated, reducing disease spread and decreasing incidence rates. In contrast, low-SDI regions have poor accessibility to drug resistance testing services and low population coverage in the early stages. As detection coverage increases in low-SDI regions, more potential patients are identified, leading to an apparent increase in incidence [41]. (2) Insufficient Community Control. Low-SDI regions lack mature community management plans and have limited services for patient care and treatment. Approximately one-third of MDR-TB patients remain smear-positive at discharge, posing a risk of community transmission. These findings highlight the importance of enhancing detection coverage and improving community-based control measures, particularly in low-SDI regions, to curb the spread of MDR-TB [2].

The detection of *Mtb* strain drug resistance has traditionally relied on bacterial culture methods. However, rapid molecular diagnostic tests and sequencing technologies are now being introduced for the diagnosis of drug-resistant TB. Despite the emergence of new drugs such as bedaquiline, pretomanid, and linezolid [42], which have significantly improved the cure rates of refractory TB, the proportion of drug-resistant TB patients receiving and completing standardized treatment remains low [43].

Currently, the global registration level for rifampicin-resistant (RR)/MDR-TB treatment corresponds to only 43% of newly diagnosed RR/MDR-TB patients annually [2]. From 2018 to 2022, only 55% of the targeted number of MDR/RR-TB patients received treatment [2]. By the end of 2022, 40 countries had adopted the new six-month BPaLM/BPaL treatment regimen for MDR-TB/RR-TB or pre-extensively drug-resistant TB (pre-XDR-TB) [2], and 92 countries had implemented the shorter nine-month oral treatment regimen for MDR/RR-TB. The treatment success rate for RR/MDR-TB patients has shown steady improvement, increasing to 63% in 2020 from 60% in 2021, though there is still significant room for progress [2].

Clinicians should adhere to the latest TB treatment guidelines and consider early patient characteristics to predict clinical outcomes. For patients with poor prognostic features, such as thick-walled cavities or persistent positive sputum cultures at 3 months, targeted individualized measures should be implemented. Enhancing the treatment success rate for drug-resistant TB patients and reducing the risk of transmission within families and communities is a crucial control strategy [44, 45].

### Preventing latent TB infection (LTBI) can curb TB transmission

Close contacts of TB patients are highly susceptible to LTBI. Early screening and preventive treatment for these individuals are crucial measures to prevent TB transmission [46]. Individuals with LTBI do not experience adverse health effects and do not transmit *Mtb*. However, they face a continuous risk of progressing to active TB through reactivation. For those with long-term infection, the annual risk of developing active TB is relatively low, with empirical estimates ranging from 10 to 20 per 100,000 population [47].

LTBI remains widespread in regions with high TB prevalence, and reactivation can account for a significant proportion of incident TB cases. This phenomenon is observed even in countries where TB transmission has been steadily declining. It is estimated that one-quarter of the world's population is infected with LTBI, highlighting its potential as a substantial reservoir for future active TB cases. Interventions to prevent the progression of LTBI to active TB disease are critical for TB control [48].

In countries with low TB incidence, close contacts of pulmonary TB cases, such as family members, are systematically screened and treated for LTBI. The recommended treatment rate for individuals with LTBI should be at least 85%, with a completion rate of 75% [49]. However, the number of household contacts diagnosed with LTBI and prescribed preventive treatment remains very low [50]. Identifying and treating individuals with LTBI is crucial for preventing the development of active TB and controlling its spread.

Some studies suggest that in countries with severe TB epidemics or in LMICs, the cost-effectiveness of detecting and treating latent TB infections is lower than treating active TB cases. This is due to the large population with latent infections, high costs, poor acceptability, and difficulties in managing treatment. Nonetheless, addressing LTBI remains essential for comprehensive TB control efforts [2, 51].

### Control strategies developed under the guidance of the One Health approach will better curb TB transmission

In response to the persistent threat of MDR-TB and XDR-TB amid a slow decline in the TB burden, a One Health-based strategy is essential for effective and comprehensive TB control. This approach integrates interdisciplinary collaboration, environmental management, animal health monitoring, community engagement, strengthened research, robust policy support, and international cooperation [52–58].

The study has several limitations. First, it cannot avoid the inherent limitations of the GBD methodology. Data from some countries and regions, particularly for MDR-TB and XDR-TB incidence, DALYs, and mortality rates between 1990 and 2021, are missing, significantly affecting the accuracy and completeness of model estimates. Even when data are available, variations in quality, accuracy, and comparability can introduce biases [1]. For instance, the GBD 2021 dataset was released internally at the end of 2023, but comprehensive adjustments to the model parameters were made in early 2024, resulting in discrepancies between the updated data (used in this study) and data from other papers concerning TB burden [1]. Second, GBD 2021 data are model based rather than real-world data, which may result in overestimation or underestimation. Third, TB and subtype indicators for 204 countries and regions were calculated using global population standardization to ensure comparability. However, these indicators may not accurately reflect the true epidemiological situation of TB in each country. Fourth, the 95% *CI* of the EAPC may be underestimated because it estimates the average trend over the past thirty years without considering the uncertainty of these rates. Moreover, EAPC is accurate under linear trends, but when the rates show non-linear trends such as U-shaped, V-shaped, or L-shaped patterns, EAPC results can be erroneous. Fifth, a comprehensive assessment of disease burden requires broader consideration of economic, familial, and social factors. Sixth, the study did not include LTBI, HIV-positive TB cases, or TB cases resistant solely to rifampicin. Future studies should adopt multidimensional analyses to improve the accuracy and robustness of results.

## Conclusions

The study highlights that the incidence of MDR-TB and XDR-TB remained steady from 2015 but began to rise slowly between 2019 and 2021. In addition, TB incidence is notably high in women, while men exhibit higher mortality rates. To address these issues, gender-specific TB screening and treatment programs should be implemented to improve early detection and treatment outcomes. Enhancing health infrastructure and increasing funding in low SDI regions are crucial for accelerating TB elimination. Developing rapid, accurate diagnostic tools and shorter, more effective, and less toxic treatment regimens are essential for combating MDR-TB and XDR-TB. Moreover, improving public health education and community engagement can raise awareness and ensure better prevention and treatment adherence. These measures are vital for reducing the global TB burden, particularly in protecting vulnerable populations.

### Supplementary Information

Additional file 1: Table S1 The number of incidence cases of TB, DS-TB, MDR-TB, and XDR-TB in HIV-negative individuals in 2021, and percentage change of the number of incidence cases were analyzed across GBD regions. Table S2 The EAPC of ASRs for TB, DS-TB, MDR-TB, and XDR-TB in HIV-negative individuals were analyzed across five SDI regions. Table S3 ASRs of TB, DS-TB, MDR-TB, and XDR-TB in HIV-negative individuals in 2021, and percentage change of ASRs for 204 countries and territories. Table S4 The number of death cases of TB, DS-TB, MDR-TB, and XDR-TB in HIV-negative individuals in 2021, and percentage of change rates of death number for GBD regions. Table S5 The number of DALY cases of TB, DS-TB, MDR-TB, and XDR-TB in HIV-negative individuals in 2021, and percentage change of number of DALY cases were analyzed across GBD regions. Table S6 Predicted ASRs for HIV-DS-TB, HIV-MDR-TB, and HIV-XDR-TB from spanning 2022–2035, based on the Bayesian Age-Period-Cohort Model. Fig. S1 The specific mortality of TB, DS-TB, MDR-TB, and XDR-TB showed notable differences across age and gender distributions in 2021 year. Fig. S2 The specific DALY of TB, DS-TB, MDR-TB, and XDR-TB showed notable differences across age and gender distributions in 2021 year. Fig. S3 The association between the SDI and the ASIR, ASMR, and age-standardized DALY rate of TB across 204 countries and territories in 2021 year. Fig. S4 The association between the SDI and the ASIR, ASMR, and age-standardized DALY rate of DS-TB across 204 countries and territories in 2021 year. Fig. S5 The association between the SDI and the ASIR, ASMR, and age-standardized DALY rate of MDR-TB across 204 countries and territories in 2021 year. Fig. S6 The association between the SDI and the ASIR, ASMR, and age-standardized DALY rate of XDR-TB across 204 countries and territories in 2021. Fig. S7 The association between the SDI and the ASIR, ASMR, and age-standardized DALY rate from 1990 to 2021 year. Fig. S8 The association between the SDI and the ASIR, ASMR, and age-standardized DALY rate of DS-TB from 1990 to 2021 year. Fig. S9 The association between the SDI and the ASIR, ASMR, and age-standardized DALY rate of MDR-TB from 1990 to 2021 year. Fig. S10 The association between the SDI and the ASIR, ASMR, and age-standardized DALY rate of XDR-TB from 1990 to 2021 year. Fig. S11 The association between risk factors and the ASMR and age-standardized DALY rate of XDR-TB in 21 GBD regions from 1990 to 2021.

## Data Availability

The datasets analysed during the current study are available at http://ghdx.healthdata.org/gbd-results-tool.

## Abbreviations

AIDS: Acquired immune deficiency syndrome
ASIR: Age-standardized incidence rate
ASMR: Age-standardized mortality rate
ASR: Age-standardized rate
BAPC: Bayesian age-period-cohort
BCG: Bacille Calmette-Guérin
*CI*: Confidence interval
COVID-19: Corona Virus Disease 2019
DALYs: Disability-adjusted life years
DisMoD-MR: Disease-model-Bayesian meta-regression
DOTS: Directly observed treatment, short-course
DS-TB: Drug-susceptible tuberculosis
EAPC: Estimated annual percentage change
GBD: Global Burden of Disease
HIV: Human immunodeficiency virus
INLA: Integrated nested Laplace approximation
LMICs: Low- and middle-income countries
LTBI: Latent tuberculosis infection
MDR-TB: Multidrug-resistant tuberculosis without extensive drug resistance
MR-BRT: Bayesian priors, regularisation, and trimming
Mtb: Mycobacterium tuberculosis
Pre-XDR-TB: Pre-extensively drug-resistant tuberculosis
RR-TB: Rifampicin resistance tuberculosis
SDI: Sociodemographic Index
SDGs: Sustainable Development Goals
TB: Tuberculosis
UI: Uncertainty interval
WHO: World Health Organization
XDR-TB: Extensively drug-resistant tuberculosis
