# Supplementary material for: Global, regional, and national burden of HIV-negative tuberculosis, 1990–2021: findings from the Global Burden of Disease Study 2021
Source: Infect Dis Poverty. 2024 Aug 19;13:60. doi: 10.1186/s40249-024-01227-y (PMC11331668; doi:10.1186/s40249-024-01227-y)
Supplement: Supplementary file 1 — Additional file 1: Table S1 The number of incidence cases of TB, DS-TB, MDR-TB, and XDR-TB in HIV-negative individuals in 2021, and percentage change of the number of incidence cases were analyzed across GBD regions. Table S2 The EAPC of ASRs for TB, DS-TB, MDR-TB, and XDR-TB in HIV-negative individuals were analyzed across five SDI regions. Table S3 ASRs of TB, DS-TB, MDR-TB, and XDR-TB in HIV-negative individuals in 2021, and percentage change of ASRs for 204 countries and territories. Table S4 The number of death cases of TB, DS-TB, MDR-TB, and XDR-TB in HIV-negative individuals in 2021, and percentage of change rates of death number for GBD regions. Table S5 The number of DALY cases of TB, DS-TB, MDR-TB, and XDR-TB in HIV-negative individuals in 2021, and percentage change of number of DALY cases were analyzed across GBD regions. Table S6 Predicted ASRs for HIV-DS-TB, HIV-MDR-TB, and HIV-XDR-TB from spanning 2022–2035, based on the Bayesian Age-Period-Cohort Model. Fig. S1 The specific mortality of TB, DS-TB, MDR-TB, and XDR-TB showed notable differences across age and gender distributions in 2021 year. Fig. S2 The specific DALY of TB, DS-TB, MDR-TB, and XDR-TB showed notable differences across age and gender distributions in 2021 year. Fig. S3 The association between the SDI and the ASIR, ASMR, and age-standardized DALY rate of TB across 204 countries and territories in 2021 year. Fig. S4 The association between the SDI and the ASIR, ASMR, and age-standardized DALY rate of DS-TB across 204 countries and territories in 2021 year. Fig. S5 The association between the SDI and the ASIR, ASMR, and age-standardized DALY rate of MDR-TB across 204 countries and territories in 2021 year. Fig. S6 The association between the SDI and the ASIR, ASMR, and age-standardized DALY rate of XDR-TB across 204 countries and territories in 2021. Fig. S7 The association between the SDI and the ASIR, ASMR, and age-standardized DALY rate from 1990 to 2021 year. Fig. S8 The association be [file 40249_2024_1227_MOESM1_ESM.docx]

**Additional file1**

**Global, regional, and national burden of HIV-negative tuberculosis, 1990–2021: findings from the Global Burden of Disease Study 2021**

Shun-Xian Zhang^1,2†^, Feng-Yu Miao^3†^, Jian Yang^4^, Wen-Ting Zhou^5^, Shan Lv^2,6^, Fan-Na Wei^2,6^, Yu Wang^1^, Xiao-Jie Hu^1^, Ping Yin^1^, Pei-Yong Zheng^1^, Ming Yang^1^, Mei-Ti Wang^7^, Xin-Yu Feng^2,6^, Lei Duan^2,6^, Guo-Bing Yang^8^, Ji-Chun Wang^4*^, Zhen-Hui Lu^1*^

^1^ Longhua Hospital, Shanghai University of Traditional Chinese Medicine, Shanghai 200032, China.

^2^ National Institute of Parasitic Diseases at Chinese Center for Disease Control and Prevention (Chinese Center for Tropical Diseases Research); NHC Key Laboratory of Parasite and Vector Biology; WHO Collaborating Centre for Tropical Diseases; National Center for International Research on Tropical Diseases; National Key Laboratory of Intelligent Tracking and Forecasting for Infectious Diseases, Shanghai 200025, China.

^3^ Beijing Municipal Health Big Data and Policy Research Center, Beijing Institute of Hospital Management, Beijing 101100, China.

^4^ Department of Science and Technology, Chinese Center for Disease Control and Prevention; National Key Laboratory of Intelligent Tracking and Forecasting for Infectious Diseases, Beijing 102206, China.

^5^ National Health Commission (NHC) Key Laboratory of Biosafety; National Institute for Viral Disease Control and Prevention, Chinese Center for Disease Control and Prevention; National Key Laboratory of Intelligent Tracking and Forecasting for Infectious Diseases, Beijing 102206, China.

^6^ School of Global Health, Chinese Center for Tropical Diseases Research-Shanghai Jiao Tong University School of Medicine, Shanghai 200025, China.

^7^ Shanghai Mental Health Center, Shanghai Jiao Tong University School of Medicine, Shanghai 200032, China.

^8^ Gansu Provincial Center for Disease Control and Prevention, Lanzhou 730000, China.

^†^ Shun-Xian Zhang and Feng-Yu Miao contributed equally to this work.

* Correspondence: Ji-Chun Wang, wangjc@chinacdc.cn;

Zhen-Hui Lu, [Dr_luzh@shutcm.edu.cn.](mailto:Dr_luzh@shutcm.edu.cnl)

1 Search method for the data of the study.

2 Table S1. The number of incidence cases of TB, DS-TB, MDR-TB, and XDR-TB in HIV-negative individuals in 2021, and percentage change of the number of incidence cases were analyzed across GBD regions.

3 Table S2. The EAPC of ASRs for TB, DS-TB, MDR-TB, and XDR-TB in HIV-negative individuals were analyzed across five SDI regions.

4 Table S3. ASRs of TB, DS-TB, MDR-TB, and XDR-TB in HIV-negative individuals in 2021, and percentage change of ASRs for 204 countries and territories.

5 Table S4. The number of death cases of TB, DS-TB, MDR-TB, and XDR-TB in HIV-negative individuals in 2021, and percentage of change rates of death number for GBD regions.

6 Table S5. The number of DALY cases of TB, DS-TB, MDR-TB, and XDR-TB in HIV-negative individuals in 2021, and percentage change of number of DALY cases were analyzed across GBD regions.

7 Table S6. Predicted ASRs for HIV-DS-TB, HIV-MDR-TB, and HIV-XDR-TB from spanning 2022－2035, based on the Bayesian Age-Period-Cohort Model

8 Fig. S1. The specific mortality of TB, DS-TB, MDR-TB, and XDR-TB showed notable differences across age and gender distributions in 2021 year.

9 Fig.S2. The specific DALY of TB, DS-TB, MDR-TB, and XDR-TB showed notable differences across age and gender distributions in 2021 year.

10 Fig.S3. The association between the SDI and the ASIR, ASMR, and age-standardized DALY rate of TB across 204 countries and territories in 2021 year.

11 Fig.S4. The association between the SDI and the ASIR, ASMR, and age-standardized DALY rate of DS-TB across 204 countries and territories in 2021 year.

12 Fig.S5. The association between the SDI and the ASIR, ASMR, and age-standardized DALY rate of MDR-TB across 204 countries and territories in 2021 year.

13 Fig. S6. The association between the SDI and the ASIR, ASMR, and age-standardized DALY rate of XDR-TB across 204 countries and territories in 2021.

14 Fig.S7. The association between the SDI and the ASIR, ASMR, and age-standardized DALY rate from 1990 to 2021 year.

15 Fig. S8. The association between the SDI and the ASIR, ASMR, and age-standardized DALY rate of DS-TB from 1990 to 2021 year.

16 Fig.S9. The association between the SDI and the ASIR, ASMR, and age-standardized DALY rate of MDR-TB from 1990 to 2021 year.

17 Fig. S10. The association between the SDI and the ASIR, ASMR, and age-standardized DALY rate of XDR-TB from 1990 to 2021 year.

18 Fig. S11. The association between risk factors and the ASMR and age-standardized DALY rate of XDR-TB in 21 GBD regions from 1990 to 2021.

**Search method for the data of the study**

The specific search criteria in the "Search" interface were as follows: GBD Estimate (Cases of mortality or injury, risk factor), Measure (Incidence, Deaths, DALYs), Metric(Number, Percent, Rate), Cause(Tuberculosis, Drug-susceptible Tuberculosis, Multidrug-resistant Tuberculosis without extensive drug resistance, Extensively drug-resistant Tuberculosis), Location (Global, All countries and regions, Different SDI regions, 204 countries and territories), Age (All ages, Age-standardized, <5 years, 5－9 years, 10－14 years, 15－19 years, 20－24 years, 25－29 years, 30－34 years, 35－39 years, 40－44 years, 45－49 years, 50－54 years, 55－59 years, 60－64 years, 65－69 years, 70－74 years, 75－79 years, 80－84 years, 85－89 years, 90－94 years, 95+ years), Sex(Both, male, female), Year(1990－2021, 2010－2021, and each year from 1990 to 2021). As XDR-TB diagnosis and confirmation were only recommended by WHO post-1991, XDR-TB data became available in 1991, including age-standardized incidence rate (ASIR), incidence numbers, andage-standardized DALY rate, DALY counts. From 1993 onwards, data on the age-standardized death rates(ASDR) and death cases also became available. Hence, for data pertaining to XDR-TB, the data from 2010 to 2021 was used to analyze the annual changes in ASIR, ASDR, and age-standardized DALY rate for XDR-TB. For TB, DS-TB, and MDR-TB, the date from 1990 to 2021 was used to assess the annual average rate changes.

Table S1.The number of incidence cases of TB, DS-TB, MDR-TB, and XDR-TB in HIV-negative individuals in 2021, and percentage change of the number of incidence cases were analyzed across GBD regions.

| Region | HIV-negative TB | | DS-TB | | MDR-TB | | XDR-TB | |
| --- | --- | --- | --- | --- | --- | --- | --- | --- |
| Region | Incidence cases  (individuals)  (95% UI),  2021. | Percentage  change of incidence case  (95% UI).  1990－2021. | Incidence cases  (individuals)  (95% UI),  2021. | Percentage  change of incidence case  (95% UI).  1990－2021. | Incidence cases (individuals)  (95% UI),  2021. | Percentage  change of incidence case  (95% UI).  1990－2021. | Incidence cases (individuals)  (95% UI),  2021. | Percentage  change of incidence case  (95% UI).  2010－2021. |
| Global | 8407133(9393767, 7519793) | -0.02(-0.07, 0.03) | 7939417(7009965, 9018495) | -0.07(-0.12, -0.02) | 443680(259196, 766545) | 7.54(2.28, 21.55) | 24036(17144, 34587) | 0.23(-0.1, 0.75) |
| Male | 4684699(4183366, 5243434) | 0.05(0, 0.1) | 4417348(3902662, 5034961) | 7.46(2.3, 21.28) | 252065(149701, 429851) | 0(-0.06, 0.05) | 15286(10953, 21940) | 0.21(-0.13, 0.69) |
| Female | 3722434(3313147, 4158319) | -0.1(-0.15, -0.05) | 3522069(3081670, 4004716) | 7.65(2.29, 21.94) | 191614(108257, 339501) | -0.14(-0.2, -0.09) | 8751(6100, 12830) | 0.27(-0.09, 0.85) |
| East Asia | 707421(786489, 630578) | -0.43(-0.48, -0.38) | 675453(580945, 762720) | -0.44(-0.51, -0.38) | 29345(7477, 82540) | -0.2(-0.85, 2.58) | 2624(674, 7312) | -0.01(-0.71, 1.51) |
| Southeast Asia | 1264295(1395423, 1148247) | 0.08(0.03, 0.13) | 1237421(1117900, 1362517) | 0.06(0.01, 0.11) | 24442(12728, 42288) | 8.18(1.98, 24.01) | 2432(1281, 4185) | 0.3(-0.35, 1.37) |
| Oceania | 13738(15185, 12451) | 0.78(0.69, 0.88) | 13210(11871, 14675) | 0.71(0.59, 0.82) | 469(140, 1073) | 113.96(19.11, 599.78) | 59(17, 133) | 3.77(0.09, 14.52) |
| Central Asia | 50921(58501, 44323) | -0.18(-0.26, -0.09) | 38128(31898, 45461) | -0.38(-0.48, -0.29) | 10555(7086, 15013) | 99.53(28.82, 300.91) | 2238(1513, 3177) | 0.02(-0.34, 0.43) |
| Central Europe | 19962(23026, 17074) | -0.58(-0.61, -0.54) | 19518(16635, 22467) | -0.59(-0.62, -0.55) | 366(169, 692) | 0.41(-0.51, 2.95) | 79(37, 148) | -0.19(-0.7, 1.09) |
| Eastern Europe | 136684(165443, 113801) | -0.43(-0.48, -0.36) | 24136(20970, 27812) | -0.63(-0.7, -0.55) | 39543(24759, 58985) | 13.78(4.24, 37.88) | 8315(5207, 12402) | 0.03(-0.38, 0.66) |
| High-income Asia Pacific | 47258(54406, 40218) | -0.46(-0.51, -0.4) | 46643(39801, 53689) | -0.46(-0.51, -0.4) | 535(146, 1675) | 0.08(-0.77, 3.87) | 80(21, 246) | 0.21(-0.62, 2.52) |
| Australasia | 1693(1981, 1447) | -0.08(-0.15, -0.01) | 1638(1398, 1919) | -0.11(-0.18, -0.03) | 49(19, 111) | 4.41(0.41, 24.41) | 6(2, 14) | 2.05(-0.18, 9.51) |
| Western Europe | 24818(28634, 21587) | -0.55(-0.57, -0.53) | 24136(20970, 27812) | -0.56(-0.58, -0.54) | 592(382, 923) | 0.34(-0.27, 1.62) | 89(58, 138) | 0.24(-0.19, 1.01) |
| Southern Latin America | 9825(11485, 8548) | -0.27(-0.32, -0.21) | 9683(8350, 11378) | -0.27(-0.33, -0.22) | 123(35, 384) | 1.78(-0.48, 11.77) | 18(5, 55) | 0.29(-0.61, 2.29) |
| High-income North America | 10051(11737, 8606) | -0.23(-0.26, -0.2) | 9886(8433, 11535) | -0.22(-0.26, -0.18) | 146(60, 351) | -0.62(-0.86, 0.14) | 19(8, 46) | 0.89(-0.23, 3.63) |
| Caribbean | 16552(18617, 14615) | -0.04(-0.12, 0.04) | 16465(14520, 18538) | -0.04(-0.12, 0.04) | 80(28, 187) | 0.13(-0.71, 2.86) | 7(2, 17) | 1.1(-0.31, 4.72) |
| Andean Latin America | 40744(47864, 35282) | -0.39(-0.45, -0.33) | 37736(32547, 44811) | -0.43(-0.5, -0.37) | 2777(1369, 5504) | 4.16(0.71, 15.72) | 231(115, 455) | 0.67(-0.15, 2.08) |
| Central Latin America | 50152(57508, 44042) | -0.06(-0.13, 0.02) | 48557(42529, 55891) | -0.09(-0.16, -0.01) | 1471(623, 2950) | 18.28(5.36, 56.07) | 125(53, 244) | 0.88(-0.08, 2.53) |
| Tropical Latin America | 72891(85106, 62717) | 0(-0.08, 0.09) | 70443(59989, 82947) | -0.03(-0.14, 0.05) | 2271(516, 6514) | 64.85(7.67, 529.9) | 177(40, 507) | 1.25(-0.44, 5.23) |
| North Africa and Middle East | 163762(188973, 143316) | -0.18(-0.25, -0.1) | 158856(139051, 182982) | -0.2(-0.28, -0.13) | 4709(2590, 8876) | 8.28(2.56, 23.99) | 198(103, 405) | 0.11(-0.48, 1.28) |
| South Asia | 3569825(4088868, 3155740) | 0.02(-0.06, 0.11) | 3311451(2813452, 3884321) | -0.05(-0.17, 0.05) | 251660(78448, 566023) | 78.45(11.78, 391.64) | 6714(2182, 15024) | 0.85(-0.34, 3.17) |
| Central Sub-Saharan Africa | 400096(447996, 356013) | 0.72(0.62, 0.83) | 390858(345839, 439819) | 0.69(0.57, 0.79) | 9162(2772, 24002) | 11.93(1.67, 68.98) | 76(24, 199) | 0.65(-0.47, 4.1) |
| Eastern Sub-Saharan Africa | 860550(974844, 755465) | 0.18(0.11, 0.25) | 827721(723576, 940628) | 0.13(0.06, 0.21) | 32545(19409, 53305) | 47.3(15.44, 123.54) | 283(172, 451) | 0.94(0.16, 2.13) |
| Southern Sub-Saharan Africa | 344138(393275, 300917) | 0.28(0.19, 0.38) | 331566(289443, 381154) | 0.24(0.15, 0.34) | 18795(8263, 40318) | 10.61(2.2, 47.7) | 98(46, 206) | 0.41(-0.43, 2.34) |
| Western Sub-Saharan Africa | 601757(676842, 533192) | 0.15(0.06, 0.26) | 581222(513586, 656586) | 0.11(0.02, 0.21) | 20368(9571, 42196) | 12.23(4.19, 33.3) | 167(79, 360) | 0.26(-0.34, 1.56) |
| High-middle SDI | 543004(479141, 613318) | -0.37(-0.41, -0.33) | 475841(414572, 545696) | -0.44(-0.49, -0.39) | 57213(35366, 90233) | 2.64(0.36, 9.62) | 9949(6272, 14413) | -0.01(-0.37, 0.47) |
| High SDI | 128646(112436, 145758) | -0.39(-0.42, -0.35) | 125481(108696, 142184) | -0.39(-0.43, -0.36) | 2829(1648, 5155) | 0.13(-0.45, 1.37) | 337(213, 583) | 0.16(-0.29, 1) |
| Low-middle SDI | 3231462(2883990, 3665299) | -0.04(-0.1, 0.02) | 3046238(2646928, 3520100) | -0.1(-0.18, -0.03) | 179355(72252, 382932) | 50.06(13.95, 154.86) | 5869(2895, 11523) | 0.63(-0.15, 2.09) |
| Low SDI | 1974063(1753395, 2214868) | 0.21(0.15, 0.27) | 1888126(1654993, 2125208) | 0.16(0.09, 0.22) | 84409(49698, 144492) | 24.76(10.73, 56.27) | 1528(780, 2989) | 0.72(-0.2, 2.81) |
| Middle SDI | 2525796(2270799, 2798413) | 0.01(-0.05, 0.07) | 2399674(2144745, 2697854) | -0.03(-0.09, 0.03) | 119777(57287, 219650) | 3.44(0.4, 14.4) | 6345(3751, 10443) | 0.35(-0.18, 1.27) |

Notes: Globally, the World Health Organization began to recommend the XDR-TB surveillance in 1991. Consequently, the number of incidence cases of XDR-TB has been tracked and reported since 1991. However, the GBD 2021 database provides total percentage change data for the periods 1990－2000, 2000－2021, 1990－2021, 2010－2021, and 2019－2021. Therefore, percentage change of number of incidence cases for XDR-TB of 2010－2021 were used in the study (Abbreviations: DS-TB: drug-susceptible tuberculosis. GBD: Global Burden of Disease. HIV: human immunodeficiency virus. MDR-TB: multidrug-resistant tuberculosis without extensive drug resistance. SDI: Sociodemographic Index. TB: Tuberculosis. UI: Uncertainty interval. XDR-TB: extensively drug-resistant tuberculosis).

Table S2. The EAPC of ASR for TB, DS-TB, MDR-TB, and XDR-TB in HIV-negative individuals were analyzed across five SDI regions.

|  | HIV-negative TB | | | DS-TB | | |
| --- | --- | --- | --- | --- | --- | --- |
| SDI  regions | ASIR . EAPC (95% CI). 1990－2021 | ASMR . EAPC (95% CI). 1990－2021 | Age-standardized  DALY rate (95% *CI*). 1990－2021 | ASIR EAPC (95% CI). 1990－2021 | ASMR . EAPC (95% CI). 1990－2021 | Age-standardized  DALY rate (95% *CI*). 1990－2021 |
| High SDI | -2.71(-2.76, -2.65) | -5.55(-5.75, -5.36) | -5.69 (-5.88, -5.51 | -2.69(-2.76, -2.61) | -5.55(-5.75, -5.34) | -5.70 (-5.91, -5.48) |
| High-middle SDI | -3.02( -3.30, 2.73) | -5.35(CI: -5.75, -5.36) | -5.16 (-5.68, -4.64) | -3.30(-3.49, -3.12) | -5.75(-6.06--5.43) | -5.56 (-5.90, -5.22) |
| Middle SDI | -1.99(-2.07, -1.92) | -4.41(-4.58, -4.23) | -4.26 (-4.43, -4.09) | -2.05(-2.13, -1.98) | -4.54(-4.66--4.41) | -4.39 (-4.51, -4.26) |
| Low-middle SDI | -2.50( -2.60, -2.40) | -3.71( -3.88, -3.55) | -3.84 (4.01, -3.68) | -2.68(-2.77, -2.60) | -4.07(-4.19--3.94) | -4.18 (-4.31, -4.06) |
| Low SDI | -2.18( -2.34, -2.02) | -3.28(-3.46, -3.10) | -3.54 (-3.72, -3.35) | -2.32(-2.46, -2.17) | -3.54(-3.69--3.39) | -3.78 (-3.94, -3.63) |
| SDI  regions | MDR-TB | | | XDR-TB | | |
| High SDI | ASIR . EAPC (95% CI). 1990－2021 | ASMR . EAPC (95% CI). 1990－2021 | Age-standardized  DALY rate (95% *CI*). 1990－2021 | ASIR . EAPC (95% CI). 1991－2021 | ASMR . EAPC (95% CI). 1991－2021 | Age-standardized  DALY rate (95% *CI*). 1993－2021 |
| High-middle SDI | -3.33(-4.30, -2.26) | -6.13(6.71, -5.08) | -5.98(-6.99, -4.96) | 6.15(3.65, 8.71) | 1.52(-0.59, 3.68) | 7.89( 2.19, 13.90) |
| Middle SDI | -0.25( -1.92, 1.45) | -3.67(-5.42, -1.88) | -3.30(-5.15, -1.40) | 7.36(5.02, 9.75) | 4.53(1.36, 7.60) | 12.34(5.44, 19.69) |
| Low-middle SDI | -0.26(-1.35, 0.85) | -2.51( -3.70, -1.31) | -2.34(-5.15, -1.40) | 7.36(5.02, 9.75) | 3.50(1.37, 5.68) | 10.43(4.63, 16.55) |
| Low SDI | 6.02(3.61, 6.49) | 4.80(2.19, 7.48) | 4.60(2.02, 7.24) | 14.10(10.06, 18.29) | 9.78(6.57, 13.09) | 18.42(10.63, 26.75) |
|  | 4.39( 2.42, 6.40) | 3.61(1.49, 5.77) | 3.25(1.13, 5.42) | 15.30(10.90, 19.67) | 10.88(7.65, 14.19) | 19.55(11.51, 28.17) |

Notes: Globally, the World Health Organization began to recommend the XDR-TB surveillance in 1991. Consequently, the ASIR and age-standardized DALY rate has been tracked and reported since 1991, and the ASMRs of XDR-TB has been tracked and reported since 1993 (Abbreviations: ASIR: age-standardized incidence rate. ASMR: Age-standardized mortality rates. ASR: age-standardized rate. CI: Confidence interval. DS-TB: drug-susceptible tuberculosis. EAPC: annual percentage changes. HIV: human immunodeficiency virus. MDR-TB: multidrug-resistant tuberculosis without extensive drug resistance. SDI: Sociodemographic Index. TB: Tuberculosis. UI: Uncertainty interval. XDR-TB: extensively drug-resistant tuberculosis).

Table S3. ASRs of TB, DS-TB, MDR-TB, and XDR-TB in HIV-negative individuals in 2021, and percentage change of ASRs for 204 countries and territories .

| ID | Sequence | Feature | Year | Index | Disease | Countries and territories | Value (percentage) (95%: UI) |
| --- | --- | --- | --- | --- | --- | --- | --- |
| 1 | 1 | low | 2021 | incidence rate | TB | San Marino | 2(1.71, 2.35) |
| 2 | 2 | low | 2021 | incidence rate | TB | United States of America | 2.08(1.78, 2.46) |
| 3 | 3 | low | 2021 | incidence rate | TB | Puerto Rico | 2.33(2.01, 2.74) |
| 4 | 4 | low | 2021 | incidence rate | TB | Israel | 2.68(2.3, 3.14) |
| 5 | 5 | low | 2021 | incidence rate | TB | Jamaica | 3.24(2.82, 3.74) |
| 6 | 1 | high | 2021 | incidence rate | DS-TB | Somalia | 600.76(505.93, 703.81) |
| 7 | 2 | high | 2021 | incidence rate | DS-TB | Central African Republic | 583.74(528.65, 634.31) |
| 8 | 3 | high | 2021 | incidence rate | DS-TB | Eritrea | 514(439.86, 594.4) |
| 9 | 4 | high | 2021 | incidence rate | DS-TB | Lesotho | 477.43(413.06, 544.95) |
| 10 | 5 | high | 2021 | incidence rate | DS-TB | South Africa | 444.05(389.73, 503.79) |
| 11 | 1 | low | 2021 | incidence rate | DS-TB | San Marino | 1.98(1.69, 2.31) |
| 12 | 2 | low | 2021 | incidence rate | DS-TB | United States of America | 2.04(1.74, 2.41) |
| 13 | 3 | low | 2021 | incidence rate | DS-TB | Puerto Rico | 2.3(1.99, 2.69) |
| 14 | 4 | low | 2021 | incidence rate | DS-TB | Israel | 2.52(2.09, 2.97) |
| 15 | 5 | low | 2021 | incidence rate | DS-TB | Greece | 3.13(2.61, 3.68) |
| 16 | 1 | high | 2021 | incidence rate | MDR-TB | Somalia | 57.25(14.12, 169.56) |
| 17 | 2 | high | 2021 | incidence rate | MDR-TB | Eswatini | 45.56(10.83, 118.38) |
| 18 | 3 | high | 2021 | incidence rate | MDR-TB | Lesotho | 36.03(10.08, 93.29) |
| 19 | 4 | high | 2021 | incidence rate | MDR-TB | Mozambique | 25.91(6.93, 60.75) |
| 20 | 5 | high | 2021 | incidence rate | MDR-TB | Comoros | 25.5(3.4, 79.33) |
| 21 | 1 | low | 2021 | incidence rate | MDR-TB | Slovenia | 0(0, 0.01) |
| 22 | 2 | low | 2021 | incidence rate | MDR-TB | Andorra | 0.01(0, 0.04) |
| 23 | 3 | low | 2021 | incidence rate | MDR-TB | Barbados | 0.01(0, 0.06) |
| 24 | 4 | low | 2021 | incidence rate | MDR-TB | United States Virgin Islands | 0.02(0, 0.08) |
| 25 | 5 | low | 2021 | incidence rate | MDR-TB | Croatia | 0.02(0.01, 0.07) |
| 26 | 1 | high | 2021 | incidence rate | XDR-TB | Republic of Moldova | 4.43(2.62, 6.55) |
| 27 | 2 | high | 2021 | incidence rate | XDR-TB | Kyrgyzstan | 4.25(1.41, 8.44) |
| 28 | 3 | high | 2021 | incidence rate | XDR-TB | Ukraine | 3.83(1.7, 6.58) |
| 29 | 4 | high | 2021 | incidence rate | XDR-TB | Russian Federation | 3.62(1.84, 5.62) |
| 30 | 5 | high | 2021 | incidence rate | XDR-TB | Uzbekistan | 2.58(0.84, 5.13) |
| 31 | 1 | low | 2021 | incidence rate | XDR-TB | Slovenia | 0.00(0.00, 0.00) |
| 32 | 2 | low | 2021 | incidence rate | XDR-TB | Barbados | 0.00(0.00, 0.00) |
| 33 | 3 | low | 2021 | incidence rate | XDR-TB | Andorra | 0.00(0.00, 0.00) |
| 34 | 4 | low | 2021 | incidence rate | XDR-TB | United States Virgin Islands | 0(0, 0.01) |
| 35 | 5 | low | 2021 | incidence rate | XDR-TB | Puerto Rico | 0(0, 0.01) |
| 36 | 1 | high | 2021 | Death rate | TB | Central African Republic | 264.97(173.66, 356.79) |
| 37 | 2 | high | 2021 | Death rate | TB | Somalia | 264.82(158.28, 456.76) |
| 38 | 3 | high | 2021 | Death rate | TB | Lesotho | 213.42(143.05, 274.31) |
| 39 | 4 | high | 2021 | Death rate | TB | Eritrea | 143.29(95.25, 220) |
| 40 | 5 | high | 2021 | Death rate | TB | Mozambique | 138.1(96.69, 180.22) |
| 41 | 1 | low | 2021 | Death rate | TB | Bermuda | 0.03(0.02, 0.03) |
| 42 | 2 | low | 2021 | Death rate | TB | New Zealand | 0.07(0.06, 0.07) |
| 43 | 3 | low | 2021 | Death rate | TB | Andorra | 0.07(0.05, 0.09) |
| 44 | 4 | low | 2021 | Death rate | TB | Malta | 0.08(0.07, 0.09) |
| 45 | 5 | low | 2021 | Death rate | TB | Luxembourg | 0.13(0.11, 0.14) |
| 46 | 1 | high | 2021 | Death rate | DS-TB | Central African Republic | 256.59(168.59, 347.82) |
| 47 | 2 | high | 2021 | Death rate | DS-TB | Somalia | 226.71(126.3, 382.94) |
| 48 | 3 | high | 2021 | Death rate | DS-TB | Lesotho | 192.43(129.58, 252.95) |
| 49 | 4 | high | 2021 | Death rate | DS-TB | Eritrea | 131.05(82.39, 208.69) |
| 50 | 5 | high | 2021 | Death rate | DS-TB | Mozambique | 122.27(81.44, 166.56) |
| 51 | 1 | low | 2021 | Death rate | DS-TB | Bermuda | 0.03(0.03, 0.03) |
| 52 | 2 | low | 2021 | Death rate | DS-TB | New Zealand | 0.06(0.05, 0.07) |
| 53 | 3 | low | 2021 | Death rate | DS-TB | Andorra | 0.07(0.05, 0.09) |
| 54 | 4 | low | 2021 | Death rate | DS-TB | Malta | 0.08(0.07, 0.09) |
| 55 | 5 | low | 2021 | Death rate | DS-TB | Luxembourg | 0.12(0.1, 0.14) |
| 56 | 1 | high | 2021 | Death rate | MDR-TB | Somalia | 37.6(7.63, 109.59) |
| 57 | 2 | high | 2021 | Death rate | MDR-TB | Lesotho | 20.7(4.74, 58.94) |
| 58 | 3 | high | 2021 | Death rate | MDR-TB | Eswatini | 17.19(3.55, 44.64) |
| 59 | 4 | high | 2021 | Death rate | MDR-TB | Mozambique | 15.61(3.55, 41.42) |
| 60 | 5 | high | 2021 | Death rate | MDR-TB | Eritrea | 12.06(1.64, 38.94) |
| 61 | 1 | low | 2021 | Death rate | MDR-TB | Bermuda | 0.00(0.00, 0.00) |
| 62 | 2 | low | 2021 | Death rate | MDR-TB | Andorra | 0.00(0.00, 0.00) |
| 63 | 3 | low | 2021 | Death rate | MDR-TB | Slovenia | 0.00(0.00, 0.00) |
| 64 | 4 | low | 2021 | Death rate | MDR-TB | Malta | 0.00(0.00, 0.00) |
| 65 | 5 | low | 2021 | Death rate | MDR-TB | New Zealand | 0.00(0.00, 0.00) |
| 66 | 1 | high | 2021 | Death rate | XDR-TB | Mongolia | 1.01(0.22, 2.62) |
| 67 | 2 | high | 2021 | Death rate | XDR-TB | Tajikistan | 0.88(0.3, 1.79) |
| 68 | 3 | high | 2021 | Death rate | XDR-TB | Kyrgyzstan | 0.8(0.28, 1.44) |
| 69 | 4 | high | 2021 | Death rate | XDR-TB | Turkmenistan | 0.79(0.25, 1.64) |
| 70 | 5 | high | 2021 | Death rate | XDR-TB | Myanmar | 0.68(0.15, 1.85) |
| 71 | 1 | low | 2021 | Death rate | XDR-TB | Bermuda | 0.00(0.00, 0.00) |
| 72 | 2 | low | 2021 | Death rate | XDR-TB | Andorra | 0.00(0.00, 0.00) |
| 73 | 3 | low | 2021 | Death rate | XDR-TB | Slovenia | 0.00(0.00, 0.00) |
| 74 | 4 | low | 2021 | Death rate | XDR-TB | Malta | 0.00(0.00, 0.00) |
| 75 | 5 | low | 2021 | Death rate | XDR-TB | Barbados | 0.00(0.00, 0.00) |
| 76 | 1 | high | 2021 | DALY rate | TB | Central African Republic | 9243.75(6246.04, 12335.08) |
| 77 | 2 | high | 2021 | DALY rate | TB | Lesotho | 7772.72(5196.89, 10011.22) |
| 78 | 3 | high | 2021 | DALY rate | TB | Somalia | 7691.18(4664.62, 13131.82) |
| 79 | 4 | high | 2021 | DALY rate | TB | Eritrea | 4645.73(3154.95, 7158.24) |
| 80 | 5 | high | 2021 | DALY rate | TB | Zimbabwe | 4427.65(2879.56, 5875.1) |
| 81 | 1 | low | 2021 | DALY rate | TB | Andorra | 1.78(1.27, 2.4) |
| 82 | 2 | low | 2021 | DALY rate | TB | Malta | 3.44(2.86, 4.26) |
| 83 | 3 | low | 2021 | DALY rate | TB | New Zealand | 3.49(2.79, 4.42) |
| 84 | 4 | low | 2021 | DALY rate | TB | Luxembourg | 3.73(3.24, 4.37) |
| 85 | 5 | low | 2021 | DALY rate | TB | San Marino | 3.97(2.8, 5.33) |
| 86 | 1 | high | 2021 | DALY rate | DS-TB | Central African Republic | 8957.36(6012.69, 12141.22) |
| 87 | 2 | high | 2021 | DALY rate | DS-TB | Lesotho | 7016.62(4703.23, 9224.74) |
| 88 | 3 | high | 2021 | DALY rate | DS-TB | Somalia | 6609.67(3723.69, 11318.37) |
| 89 | 4 | high | 2021 | DALY rate | DS-TB | Eritrea | 4269.24(2693.43, 6778.97) |
| 90 | 5 | high | 2021 | DALY rate | DS-TB | Zimbabwe | 4030.88(2468.7, 5492.73) |
| 91 | 1 | low | 2021 | DALY rate | DS-TB | Andorra | 1.77(1.27, 2.38) |
| 92 | 2 | low | 2021 | DALY rate | DS-TB | New Zealand | 3.39(2.68, 4.3) |
| 93 | 3 | low | 2021 | DALY rate | DS-TB | Malta | 3.39(2.83, 4.15) |
| 94 | 4 | low | 2021 | DALY rate | DS-TB | Luxembourg | 3.57(2.98, 4.23) |
| 95 | 5 | low | 2021 | DALY rate | DS-TB | San Marino | 3.86(2.67, 5.18) |
| 96 | 1 | high | 2021 | DALY rate | MDR-TB | Somalia | 1066.78(219.93, 3124.42) |
| 97 | 2 | high | 2021 | DALY rate | MDR-TB | Lesotho | 745.74(173.08, 2097.96) |
| 98 | 3 | high | 2021 | DALY rate | MDR-TB | Eswatini | 619.2(127.73, 1575.29) |
| 99 | 4 | high | 2021 | DALY rate | MDR-TB | Mozambique | 463.41(106.92, 1241.77) |
| 100 | 5 | high | 2021 | DALY rate | MDR-TB | Zimbabwe | 391.37(55.07, 1266.68) |
| 101 | 1 | low | 2021 | DALY rate | MDR-TB | Andorra | 0.01(0, 0.03) |
| 102 | 2 | low | 2021 | DALY rate | MDR-TB | Slovenia | 0.01(0, 0.04) |
| 103 | 3 | low | 2021 | DALY rate | MDR-TB | Malta | 0.04(0, 0.15) |
| 104 | 4 | low | 2021 | DALY rate | MDR-TB | Bermuda | 0.04(0, 0.21) |
| 105 | 5 | low | 2021 | DALY rate | MDR-TB | Iceland | 0.08(0.01, 0.28) |
| 106 | 1 | high | 2021 | DALY rate | XDR-TB | Mongolia | 43.31(9.55, 109.82) |
| 107 | 2 | high | 2021 | DALY rate | XDR-TB | Tajikistan | 38.76(13.65, 77.66) |
| 108 | 3 | high | 2021 | DALY rate | XDR-TB | Turkmenistan | 36.73(11.87, 75.54) |
| 109 | 4 | high | 2021 | DALY rate | XDR-TB | Kyrgyzstan | 33.48(11.73, 60.26) |
| 110 | 5 | high | 2021 | DALY rate | XDR-TB | Ukraine | 26.54(11.65, 45.57) |
| 111 | 1 | low | 2021 | DALY rate | XDR-TB | Andorra | 0.00(0.00, 0.01) |
| 112 | 2 | low | 2021 | DALY rate | XDR-TB | Bermuda | 0.00(0.00, 0.02) |
| 113 | 3 | low | 2021 | DALY rate | XDR-TB | Slovenia | 0.00(0.00, 0.02) |
| 114 | 4 | low | 2021 | DALY rate | XDR-TB | Malta | 0.01(0.00, 0.03) |
| 115 | 5 | low | 2021 | DALY rate | XDR-TB | Barbados | 0.01(0.00, 0.06) |
| 116 | 1 | high | 1990-2021 | incidence rate | Tuberculosis | Philippines | 0.29(0.24, 0.34) |
| 117 | 2 | high | 1990-2021 | incidence rate | Tuberculosis | Lesotho | -0.06(-0.17, 0.07) |
| 118 | 3 | high | 1990-2021 | incidence rate | Tuberculosis | Sweden | -0.08(-0.18, 0.02) |
| 119 | 4 | high | 1990-2021 | incidence rate | Tuberculosis | Georgia | -0.1(-0.18, -0.01) |
| 120 | 5 | high | 1990-2021 | incidence rate | Tuberculosis | Ukraine | -0.15(-0.24, -0.04) |
| 121 | 1 | low | 1990-2021 | incidence rate | Tuberculosis | Croatia | -0.77(-0.79, -0.75) |
| 122 | 2 | low | 1990-2021 | incidence rate | Tuberculosis | Hungary | -0.76(-0.79, -0.74) |
| 123 | 3 | low | 1990-2021 | incidence rate | Tuberculosis | Slovenia | -0.76(-0.78, -0.74) |
| 124 | 4 | low | 1990-2021 | incidence rate | Tuberculosis | Turkey | -0.76(-0.78, -0.73) |
| 125 | 5 | low | 1990-2021 | incidence rate | Tuberculosis | Estonia | -0.75(-0.77, -0.72) |
| 126 | 1 | high | 1990-2021 | incidence rate | DS-TB | Philippines | 0.25(0.18, 0.32) |
| 127 | 2 | high | 1990-2021 | incidence rate | DS-TB | Sweden | -0.11(-0.21, 0) |
| 128 | 3 | high | 1990-2021 | incidence rate | DS-TB | Lesotho | -0.12(-0.24, 0) |
| 129 | 4 | high | 1990-2021 | incidence rate | DS-TB | Greenland | -0.19(-0.27, -0.11) |
| 130 | 5 | high | 1990-2021 | incidence rate | DS-TB | Botswana | -0.19(-0.29, -0.07) |
| 131 | 1 | low | 1990-2021 | incidence rate | DS-TB | Estonia | -0.79(-0.84, -0.75) |
| 132 | 2 | low | 1990-2021 | incidence rate | DS-TB | Croatia | -0.77(-0.79, -0.75) |
| 133 | 3 | low | 1990-2021 | incidence rate | DS-TB | Hungary | -0.77(-0.79, -0.74) |
| 134 | 4 | low | 1990-2021 | incidence rate | DS-TB | Turkey | -0.77(-0.79, -0.73) |
| 135 | 5 | low | 1990-2021 | incidence rate | DS-TB | Belarus | -0.77(-0.85, -0.69) |
| 136 | 1 | high | 1990-2021 | incidence rate | MDR-TB | Kyrgyzstan | 260.68(36.58, 2418.08) |
| 137 | 2 | high | 1990-2021 | incidence rate | MDR-TB | Azerbaijan | 208.56(36.58, 1890.98) |
| 138 | 3 | high | 1990-2021 | incidence rate | MDR-TB | Uzbekistan | 201.41(25.56, 2634.13) |
| 139 | 4 | high | 1990-2021 | incidence rate | MDR-TB | Turkmenistan | 125.99(20.47, 1453.24) |
| 140 | 5 | high | 1990-2021 | incidence rate | MDR-TB | Tajikistan | 107.7(21.09, 1059.87) |
| 141 | 1 | low | 1990-2021 | incidence rate | MDR-TB | Slovenia | -0.94(-1, -0.55) |
| 142 | 2 | low | 1990-2021 | incidence rate | MDR-TB | Saint Lucia | -0.84(-0.98, 1.21) |
| 143 | 3 | low | 1990-2021 | incidence rate | MDR-TB | Barbados | -0.84(-0.99, 1.85) |
| 144 | 4 | low | 1990-2021 | incidence rate | MDR-TB | Saint Vincent and the Grenadines | -0.83(-0.98, 1.67) |
| 145 | 5 | low | 1990-2021 | incidence rate | MDR-TB | Croatia | -0.77(-0.96, 0.99) |
| 146 | 1 | high | 2010-2021 | incidence rate | XDR-TB | Papua New Guinea | 2.42(-0.27, 11.04) |
| 147 | 2 | high | 2010-2021 | incidence rate | XDR-TB | Bermuda | 1.99(-0.62, 29.55) |
| 148 | 3 | high | 2010-2021 | incidence rate | XDR-TB | Australia | 1.8(-0.37, 10.88) |
| 149 | 4 | high | 2010-2021 | incidence rate | XDR-TB | Germany | 1.72(-0.11, 5.44) |
| 150 | 5 | high | 2010-2021 | incidence rate | XDR-TB | Grenada | 1.58(-0.46, 15.78) |
| 151 | 1 | low | 2010-2021 | incidence rate | XDR-TB | Iceland | -0.79(-0.96, -0.2) |
| 152 | 2 | low | 2010-2021 | incidence rate | XDR-TB | Slovenia | -0.75(-0.95, -0.12) |
| 153 | 3 | low | 2010-2021 | incidence rate | XDR-TB | Cyprus | -0.73(-0.94, -0.12) |
| 154 | 4 | low | 2010-2021 | incidence rate | XDR-TB | Hungary | -0.71(-0.94, -0.1) |
| 155 | 5 | low | 2010-2021 | incidence rate | XDR-TB | Guam | -0.68(-0.95, 0.31) |
| 156 | 1 | high | 1990-2021 | death rate | Tuberculosis | Lesotho | 0.39(-0.14, 1.09) |
| 157 | 2 | high | 1990-2021 | death rate | Tuberculosis | Zimbabwe | 0.35(-0.2, 0.91) |
| 158 | 3 | high | 1990-2021 | death rate | Tuberculosis | Somalia | -0.11(-0.32, 0.19) |
| 159 | 4 | high | 1990-2021 | death rate | Tuberculosis | Ukraine | -0.18(-0.44, 0.14) |
| 160 | 5 | high | 1990-2021 | death rate | Tuberculosis | Eswatini | -0.21(-0.52, 0.22) |
| 161 | 1 | low | 1990-2021 | death rate | Tuberculosis | New Zealand | -0.93(-0.93, -0.92) |
| 162 | 2 | low | 1990-2021 | death rate | Tuberculosis | Hungary | -0.92(-0.93, -0.91) |
| 163 | 3 | low | 1990-2021 | death rate | Tuberculosis | Maldives | -0.91(-0.94, -0.85) |
| 164 | 4 | low | 1990-2021 | death rate | Tuberculosis | Turkey | -0.91(-0.94, -0.85) |
| 165 | 5 | low | 1990-2021 | death rate | Tuberculosis | Ecuador | -0.91(-0.93, -0.89) |
| 166 | 1 | high | 1990-2021 | death number | DS-TB | Zimbabwe | 1.08(0.17, 2.04) |
| 167 | 2 | high | 1990-2021 | death rate | DS-TB | Lesotho | 0.26(-0.24, 0.93) |
| 168 | 3 | high | 1990-2021 | death rate | DS-TB | Zimbabwe | 0.24(-0.3, 0.79) |
| 169 | 4 | high | 1990-2021 | death rate | DS-TB | Somalia | -0.23(-0.51, 0.04) |
| 170 | 5 | high | 1990-2021 | death rate | DS-TB | Central African Republic | -0.26(-0.46, 0.02) |
| 171 | 1 | low | 1990-2021 | death rate | DS-TB | New Zealand | -0.93(-0.94, -0.92) |
| 172 | 2 | low | 1990-2021 | death rate | DS-TB | Hungary | -0.92(-0.93, -0.91) |
| 173 | 3 | low | 1990-2021 | death rate | DS-TB | Ecuador | -0.92(-0.94, -0.9) |
| 174 | 4 | low | 1990-2021 | death rate | DS-TB | Turkey | -0.92(-0.95, -0.86) |
| 175 | 5 | low | 1990-2021 | death rate | DS-TB | Maldives | -0.91(-0.94, -0.86) |
| 176 | 1 | high | 1990-2021 | death rate | MDR-TB | Somalia | 111.77(14.93, 1511.71) |
| 177 | 2 | high | 1990-2021 | death rate | MDR-TB | Kyrgyzstan | 89.45(13.95, 858.79) |
| 178 | 3 | high | 1990-2021 | death rate | MDR-TB | Turkmenistan | 84.75(16.35, 1030.45) |
| 179 | 4 | high | 1990-2021 | death rate | MDR-TB | Lesotho | 82.74(9.42, 1037.16) |
| 180 | 5 | high | 1990-2021 | death rate | MDR-TB | Djibouti | 80.04(12.17, 847.52) |
| 181 | 1 | low | 1990-2021 | death rate | MDR-TB | Slovenia | -0.98(-1, -0.85) |
| 182 | 2 | low | 1990-2021 | death rate | MDR-TB | Saint Vincent and the Grenadines | -0.92(-0.99, 0.12) |
| 183 | 3 | low | 1990-2021 | death rate | MDR-TB | Saint Lucia | -0.91(-0.99, 0.31) |
| 184 | 4 | low | 1990-2021 | death rate | MDR-TB | United States of America | -0.91(-0.97, -0.7) |
| 185 | 5 | low | 1990-2021 | death rate | MDR-TB | Barbados | -0.91(-1, 0.59) |
| 186 | 1 | high | 2010-2021 | death rate | XDR-TB | Papua New Guinea | 2.27(-0.29, 10.08) |
| 187 | 2 | high | 2010-2021 | death rate | XDR-TB | Comoros | 1.36(-0.53, 9.73) |
| 188 | 3 | high | 2010-2021 | death rate | XDR-TB | Madagascar | 1.25(-0.52, 6.09) |
| 189 | 4 | high | 2010-2021 | death rate | XDR-TB | South Sudan | 1.09(-0.47, 7.26) |
| 190 | 5 | high | 2010-2021 | death rate | XDR-TB | Australia | 1.07(-0.48, 7.09) |
| 191 | 1 | low | 2010-2021 | death rate | XDR-TB | Slovenia | -0.88(-0.98, -0.58) |
| 192 | 2 | low | 2010-2021 | death rate | XDR-TB | Iceland | -0.82(-0.97, -0.32) |
| 193 | 3 | low | 2010-2021 | death rate | XDR-TB | Malta | -0.81(-0.97, -0.21) |
| 194 | 4 | low | 2010-2021 | death rate | XDR-TB | Hungary | -0.8(-0.96, -0.39) |
| 195 | 5 | low | 2010-2021 | death rate | XDR-TB | Cyprus | -0.74(-0.94, -0.18) |
| 196 | 1 | high | 1990-2021 | DALY rate | Tuberculosis | Lesotho | 0.37(-0.14, 1.05) |
| 197 | 2 | high | 1990-2021 | DALY rate | Tuberculosis | Zimbabwe | 0.34(-0.19, 0.89) |
| 198 | 3 | high | 1990-2021 | DALY rate | Tuberculosis | Ukraine | -0.1(-0.35, 0.2) |
| 199 | 4 | high | 1990-2021 | DALY rate | Tuberculosis | Somalia | -0.21(-0.41, 0.05) |
| 200 | 5 | high | 1990-2021 | DALY rate | Tuberculosis | Eswatini | -0.25(-0.54, 0.16) |
| 201 | 1 | low | 1990-2021 | DALY rate | Tuberculosis | Republic of Korea | -0.93(-0.94, -0.91) |
| 202 | 2 | low | 1990-2021 | DALY rate | Tuberculosis | Maldives | -0.92(-0.94, -0.88) |
| 203 | 3 | low | 1990-2021 | DALY rate | Tuberculosis | Turkey | -0.92(-0.95, -0.88) |
| 204 | 4 | low | 1990-2021 | DALY rate | Tuberculosis | Hungary | -0.91(-0.92, -0.89) |
| 205 | 5 | low | 1990-2021 | DALY rate | Tuberculosis | Ecuador | -0.91(-0.92, -0.89) |
| 206 | 1 | high | 1990-2021 | DALY rate | DS-TB | Lesotho | 0.24(-0.24, 0.91) |
| 207 | 2 | high | 1990-2021 | DALY rate | DS-TB | Zimbabwe | 0.23(-0.29, 0.78) |
| 208 | 3 | high | 1990-2021 | DALY rate | DS-TB | Central African Republic | -0.31(-0.5, -0.03) |
| 209 | 4 | high | 1990-2021 | DALY rate | DS-TB | Somalia | -0.32(-0.56, -0.06) |
| 210 | 5 | high | 1990-2021 | DALY rate | DS-TB | Kenya | -0.37(-0.55, -0.16) |
| 211 | 1 | low | 1990-2021 | DALY rate | DS-TB | Turkey | -0.93(-0.95, -0.89) |
| 212 | 2 | low | 1990-2021 | DALY rate | DS-TB | Republic of Korea | -0.93(-0.94, -0.91) |
| 213 | 3 | low | 1990-2021 | DALY rate | DS-TB | Maldives | -0.92(-0.94, -0.89) |
| 214 | 4 | low | 1990-2021 | DALY rate | DS-TB | Ecuador | -0.92(-0.94, -0.9) |
| 215 | 5 | low | 1990-2021 | DALY rate | DS-TB | Hungary | -0.91(-0.92, -0.9) |
| 216 | 1 | high | 1990-2021 | DALY rate | MDR-TB | Somalia | 98.83(13.27, 1315.3) |
| 217 | 2 | high | 1990-2021 | DALY rate | MDR-TB | Turkmenistan | 84.79(16.28, 1010.49) |
| 218 | 3 | high | 1990-2021 | DALY rate | MDR-TB | Lesotho | 84.02(9.89, 1063.53) |
| 219 | 4 | high | 1990-2021 | DALY rate | MDR-TB | Kyrgyzstan | 83.48(12.64, 804.2) |
| 220 | 5 | high | 1990-2021 | DALY rate | MDR-TB | Uzbekistan | 71.37(10.53, 1029.08) |
| 221 | 1 | low | 1990-2021 | DALY rate | MDR-TB | Slovenia | -0.98(-1, -0.85) |
| 222 | 2 | low | 1990-2021 | DALY rate | MDR-TB | Saint Vincent and the Grenadines | -0.92(-0.99, 0.05) |
| 223 | 3 | low | 1990-2021 | DALY rate | MDR-TB | Barbados | -0.91(-1, 0.57) |
| 224 | 4 | low | 1990-2021 | DALY rate | MDR-TB | Saint Lucia | -0.9(-0.99, 0.42) |
| 225 | 5 | low | 1990-2021 | DALY rate | MDR-TB | United States of America | -0.9(-0.97, -0.67) |
| 226 | 1 | high | 2010-2021 | DALY rate | XDR-TB | Papua New Guinea | 2.17(-0.3, 9.79) |
| 227 | 2 | high | 2010-2021 | DALY rate | XDR-TB | Comoros | 1.45(-0.53, 10.17) |
| 228 | 3 | high | 2010-2021 | DALY rate | XDR-TB | Bermuda | 1.15(-0.73, 20.94) |
| 229 | 4 | high | 2010-2021 | DALY rate | XDR-TB | Madagascar | 1.13(-0.56, 5.68) |
| 230 | 5 | high | 2010-2021 | DALY rate | XDR-TB | South Sudan | 1.12(-0.47, 7.72) |
| 231 | 1 | low | 2010-2021 | DALY rate | XDR-TB | Slovenia | -0.89(-0.98, -0.59) |
| 232 | 2 | low | 2010-2021 | DALY rate | XDR-TB | Iceland | -0.82(-0.97, -0.32) |
| 233 | 3 | low | 2010-2021 | DALY rate | XDR-TB | Hungary | -0.8(-0.96, -0.37) |
| 234 | 4 | low | 2010-2021 | DALY rate | XDR-TB | Malta | -0.79(-0.96, -0.13) |
| 235 | 5 | low | 2010-2021 | DALY rate | XDR-TB | Cyprus | -0.76(-0.95, -0.24) |

Notes: Globally, the World Health Organization began to recommend the XDR-TB surveillance in 1991. Consequently, the age-standardized incidence rate of XDR-TB has been tracked and reported since 1993, age-standardized mortality rate and age-standardized DALY rate has been tracked and reported since 1991. However, the GBD 2021 database provides total percentage change data for the periods 1990－2000, 2000－2021, 1990－2021, 2010－2021, and 2019－2021. Therefore, percentage change for XDR-TB of 2010－2021 was used in the study (Abbreviations: DS-TB: drug-susceptible tuberculosis. GBD: Global Burden of Disease. HIV: human immunodeficiency virus. MDR-TB: multidrug-resistant tuberculosis without extensive drug resistance. SDI: Sociodemographic Index. TB: Tuberculosis. UI: Uncertainty interval. XDR-TB: extensively drug-resistant tuberculosis).

Table S4. The number of death cases of TB, DS-TB, MDR-TB, and XDR-TB in HIV-negative individuals in 2021, and percentage of change rates of death number for GBD regions.

| Regions | HIV-negative TB | | DS-TB | | MDR-TB | | XDR-TB | |
| --- | --- | --- | --- | --- | --- | --- | --- | --- |
|  | Death number  (95%: UI).  2021 | Percentage  Change of death number  (95%: UI).  1990－2021. | Death number  (95%: UI).  2021 | Percentage  Change of death number  (95%: UI).  1990－2021. | Death number  (95%: UI).  2021 | Percentage  Change of death number  (95%: UI).  1990－2021. | Death number  (95%: UI).  2021 | Percentage  Change of death number  (95%: UI).  1990－2021. |
| Global | 1162796(1050008, 1313985) | -0.35(-0.43, -0.16) | 1048031(911267, 1199872) | -0.41(-0.5, -0.24) | 106818(41612, 211854) | 5.65(1.64, 15.26) | 7946(3326, 14859) | -0.02(-0.32, 0.38) |
| Male | 725385(646282, 868622) | -0.31(-0.44, 0.03) | 654023(554911, 786204) | -0.37(-0.5, -0.07) | 66092(26372, 130949) | 5.79(1.69, 16.97) | 5269(2307, 10047) | -0.07(-0.35, 0.33) |
| Female | 437411(396771, 482605) | -0.4(-0.47, -0.32) | 394008(342365, 445209) | -0.45(-0.53, -0.37) | 40726(15495, 81244) | 5.44(1.59, 15.1) | 2677(1024, 5133) | 0.1(-0.27, 0.63) |
| East Asia | 49033(40011, 61650) | -0.73(-0.8, -0.61) | 44366(33903, 56131) | -0.75(-0.82, -0.63) | 3904(1023, 9736) | -0.61(-0.91, 0.52) | 764(189, 2010) | -0.13(-0.68, 1.03) |
| Southeast Asia | 170555(147940, 199977) | -0.32(-0.43, -0.04) | 163588(140799, 190980) | -0.35(-0.45, -0.08) | 5829(2128, 12522) | 3.7(0.62, 12.81) | 1138(386, 2639) | -0.02(-0.47, 0.76) |
| Oceania | 3043(2467, 3764) | 0.35(-0.04, 0.91) | 2809(2206, 3487) | 0.25(-0.11, 0.78) | 196(43, 516) | 88.72(14.11, 522.36) | 38(8, 104) | 3.45(0.03, 13.63) |
| Central Asia | 4422(3885, 5006) | -0.39(-0.47, -0.31) | 2750(1841, 3585) | -0.62(-0.74, -0.5) | 1140(630, 1760) | 44.46(16.15, 132.35) | 532(296, 852) | -0.29(-0.5, -0.05) |
| Central Europe | 1861(1723, 2004) | -0.72(-0.74, -0.69) | 1769(1616, 1922) | -0.73(-0.75, -0.7) | 63(20, 134) | -0.16(-0.69, 1.35) | 29(9, 67) | -0.35(-0.76, 0.65) |
| Eastern Europe | 8454(7596, 9488) | -0.43(-0.49, -0.36) | 4388(2601, 6285) | -0.7(-0.82, -0.57) | 2770(1583, 3971) | 6.12(1.81, 18.54) | 1296(713, 2012) | -0.47(-0.64, -0.22) |
| High-income Asia Pacific | 6654(5427, 7548) | -0.51(-0.6, -0.44) | 6492(5312, 7343) | -0.52(-0.59, -0.45) | 128(26, 407) | -0.24(-0.85, 2.18) | 34(7, 110) | 0.25(-0.63, 2.61) |
| Australasia | 1246(1161, 1337) | -0.41(-0.46, -0.35) | 1207(1109, 1309) | -0.45(-0.52, -0.38) | 5(1, 13) | 2.1(-0.21, 12.13) | 1(0, 3) | 1.37(-0.35, 6.87) |
| Western Europe | 81(71, 89) | -0.68(-0.71, -0.66) | 75(63, 84) | -0.69(-0.72, -0.67) | 125(52, 244) | -0.1(-0.53, 0.76) | 34(14, 71) | 0.07(-0.29, 0.67) |
| Southern Latin America | 2851(2484, 3059) | -0.55(-0.59, -0.51) | 2693(2293, 2935) | -0.56(-0.6, -0.52) | 31(6, 91) | 0.6(-0.68, 5.82) | 8(2, 27) | 0.17(-0.65, 1.87) |
| High-income North America | 917(830, 969) | -0.63(-0.65, -0.61) | 883(781, 941) | -0.62(-0.65, -0.59) | 27(8, 76) | -0.83(-0.94, -0.46) | 7(2, 19) | 0.79(-0.26, 3.45) |
| Caribbean | 4334(3483, 5473) | -0.37(-0.54, -0.1) | 3693(2700, 4792) | -0.37(-0.54, -0.11) | 24(5, 75) | -0.16(-0.83, 2.18) | 4(1, 14) | 0.98(-0.37, 4.41) |
| Andean Latin America | 6094(5385, 6963) | -0.69(-0.76, -0.59) | 5688(4933, 6583) | -0.73(-0.81, -0.64) | 548(198, 1210) | 1.45(-0.13, 7.15) | 93(32, 216) | 0.34(-0.31, 1.54) |
| Central Latin America | 2532(1690, 6197) | -0.55(-0.6, -0.48) | 2505(1674, 6114) | -0.58(-0.64, -0.52) | 347(115, 755) | 8.99(2.55, 25.3) | 59(20, 135) | 0.59(-0.2, 1.91) |
| Tropical Latin America | 5825(5555, 6071) | -0.34(-0.37, -0.31) | 5426(4542, 5869) | -0.38(-0.48, -0.33) | 342(64, 1007) | 35.17(4.25, 274.14) | 58(10, 179) | 0.83(-0.48, 3.81) |
| North Africa and Middle East | 20885(16350, 29848) | -0.43(-0.54, -0.19) | 19417(14763, 28012) | -0.46(-0.58, -0.24) | 1364(432, 3417) | 7.26(1.97, 22.29) | 104(31, 299) | -0.03(-0.56, 1.28) |
| South Asia | 159769(129447, 195009) | -0.35(-0.45, -0.15) | 147187(119478, 181540) | -0.44(-0.59, -0.25) | 64155(15726, 147523) | 39.56(6.71, 197.64) | 3388(809, 8075) | 0.53(-0.42, 2.22) |
| Central Sub-Saharan Africa | 70259(49298, 101229) | 0.1(-0.17, 0.47) | 66744(46907, 96231) | 0.05(-0.23, 0.41) | 3467(865, 11863) | 7.77(0.79, 44.96) | 49(11, 168) | 0.5(-0.48, 3.46) |
| Eastern Sub-Saharan Africa | 500932(442259, 588285) | -0.28(-0.4, -0.09) | 433389(335335, 529091) | -0.33(-0.46, -0.15) | 12409(4808, 25856) | 30.47(9.67, 76.63) | 173(66, 358) | 0.75(0.07, 1.71) |
| Southern Sub-Saharan Africa | 39363(34352, 45831) | 0.33(-0.01, 0.61) | 36355(30307, 43281) | 0.24(-0.08, 0.53) | 2967(1071, 6630) | 10.44(2.31, 47.67) | 41(14, 100) | 0.14(-0.47, 1.56) |
| Western Sub-Saharan Africa | 103684(79411, 130735) | -0.13(-0.35, 0.18) | 96609(74605, 121712) | -0.19(-0.39, 0.11) | 6978(2365, 15585) | 9.25(3.31, 27.11) | 97(30, 220) | 0.22(-0.35, 1.32) |
| High-middle SDI | 37187(33136, 42712) | -0.62(-0.69, -0.5) | 30692(25615, 36972) | -0.68(-0.75, -0.56) | 4832(2645, 8293) | 0.38(-0.45, 3.35) | 1663(894, 2774) | -0.45(-0.6, -0.25) |
| High SDI | 13209(11411, 14688) | -0.6(-0.64, -0.55) | 12625(10888, 14000) | -0.61(-0.65, -0.56) | 470(184, 1027) | -0.35(-0.66, 0.26) | 114(45, 241) | 0.02(-0.33, 0.75) |
| Low-middle SDI | 505642(445263, 580689) | -0.33(-0.44, -0.12) | 450579(369912, 519134) | -0.4(-0.53, -0.22) | 51910(16134, 115997) | 29.36(8.29, 90.24) | 3153(1011, 6915) | 0.41(-0.26, 1.56) |
| Low SDI | 352026(302732, 415758) | -0.24(-0.34, -0.04) | 321839(272802, 382597) | -0.3(-0.41, -0.11) | 29313(11965, 59517) | 16.18(7.19, 34.13) | 874(293, 1936) | 0.47(-0.23, 1.96) |
| Middle SDI | 254083(227480, 300421) | -0.41(-0.5, -0.2) | 231682(200860, 277227) | -0.45(-0.54, -0.27) | 20263(7338, 41059) | 1.41(-0.18, 6.93) | 2138(835, 3992) | 0.02(-0.38, 0.52) |

Notes: Globally, the World Health Organization began to recommend the XDR-TB surveillance in 1991. Consequently, the number of death cases of XDR-TB has been tracked and reported since 1993. However, the GBD 2021 database provides total percentage change data for the periods 1990－2000, 2000－2021, 1990－2021, 2010－2021, and 2019－2021. Therefore, percentage change of number of death cases for XDR-TB of of 2010－2021 was used in the study (Abbreviations: DS-TB: drug-susceptible tuberculosis. GBD: Global Burden of Disease. HIV: human immunodeficiency virus. MDR-TB: multidrug-resistant tuberculosis without extensive drug resistance. SDI: Sociodemographic Index. TB: Tuberculosis. UI: Uncertainty interval. XDR-TB: extensively drug-resistant tuberculosis).

Table S5. The number of DALY cases of TB, DS-TB, MDR-TB, and XDR-TB in HIV-negative individuals in 2021, and percentage change of number of DALY cases were analyzed across GBD regions.

| Regions | HIV-negative TB | | DS-TB | | MDR-TB | | XDR-TB | |
| --- | --- | --- | --- | --- | --- | --- | --- | --- |
|  | DALY number  (95%: UI).  2021 | Percentage  change of DALY number  (95%: UI).  1990－2021. | DALY number  (95%: UI).  2021. | Percentage  change of DALY number  (95%: UI).  1990－2021. | DALY number  (95%: UI).  2021 | Percentage  change of DALY number  (95%: UI).  1990－2021. | DALY numbe  r (95%: UI).  2021 | Percentage  change of DALY number  (95%: UI).  2010－2021. |
| Global | 46977463(42482994, 52463556) | -0.43(-0.3, -0.49) | 42564265(36845854, 48240481) | -0.48(-0.36, -0.55) | 4125461(1707701, 8064272) | 5.09(1.52, 13.46) | 287736(124816, 531852) | -0.1(-0.37, 0.26) |
| Gender male | 28536132(25244156, 33765448) | -0.37(-0.48, -0.11) | 25841792(21957262, 30490301) | -0.43(-0.53, -0.19) | 2504513(1044212, 4884136) | 5.37(1.57, 15.23) | 189827(86537, 351058) | -0.14(-0.39, 0.21) |
| Gender female | 18441330(16705778, 20317945) | -0.5(-0.57, -0.44) | 16722473(14585069, 18907833) | -0.55(-0.61, -0.48) | 1620948(663397, 3135883) | 4.71(1.4, 12.69) | 97909(39631, 185046) | -0.02(-0.34, 0.45) |
| East Asia | 1754392(1467984, 2106441) | -0.78(-0.69, -0.82) | 1606348(1265560, 1985610) | -0.78(-0.7, -0.84) | 125299(33866, 314108) | -0.7(-0.93, 0.25) | 22745(5559, 59582) | -0.2(-0.7, 0.85) |
| Southeast Asia | 6169600(5438974, 7066376) | -0.46(-0.28, -0.53) | 5938619(5169453, 6843615) | -0.47(-0.3, -0.55) | 194366(74998, 412319) | 2.9(0.38, 10.38) | 36615(13151, 84944) | -0.09(-0.52, 0.64) |
| Oceania | 139129(115403, 170079) | 0.32(0.78, -0.02) | 128969(101796, 155201) | 0.23(0.66, -0.11) | 8542(1939, 21633) | 86.36(13.66, 516.77) | 1617(364, 4421) | 3.23(-0.01, 13.06) |
| Central Asia | 218221(192834, 248017) | -0.47(-0.39, -0.53) | 139313(99259, 176836) | -0.66(-0.56, -0.75) | 54432(31359, 83628) | 41.31(14.93, 120.78) | 24476(13826, 38958) | -0.33(-0.54, -0.11) |
| Central Europe | 61137(56195, 66306) | -0.74(-0.72, -0.76) | 58173(52764, 63974) | -0.75(-0.73, -0.77) | 2046(706, 4438) | -0.25(-0.75, 1.21) | 918(302, 2147) | -0.39(-0.79, 0.63) |
| Eastern Europe | 348174(315010, 386352) | -0.45(-0.39, -0.5) | 187322(119475, 264107) | -0.69(-0.58, -0.8) | 111078(64951, 156426) | 6.13(1.83, 18.15) | 49774(27646, 76823) | -0.52(-0.67, -0.29) |
| High-income Asia Pacific | 103931(89925, 115555) | -0.74(-0.7, -0.77) | 101448(87037, 113189) | -0.74(-0.71, -0.78) | 1972(414, 6430) | -0.59(-0.92, 0.82) | 511(111, 1593) | -0.03(-0.7, 1.85) |
| Australasia | 1906(1707, 2107) | -0.48(-0.44, -0.52) | 1776(1535, 2003) | -0.51(-0.45, -0.56) | 104(30, 262) | 1.53(-0.34, 9.81) | 26(7, 64) | 0.14(-0.66, 1.81) |
| Western Europe | 56252(51409, 60658) | -0.74(-0.73, -0.76) | 53368(48133, 57870) | -0.75(-0.73, -0.77) | 2296(1068, 4404) | -0.29(-0.61, 0.4) | 589(255, 1211) | 1.23(-0.36, 6.27) |
| Southern Latin America | 40839(38178, 43779) | -0.62(-0.59, -0.64) | 39638(36229, 42940) | -0.63(-0.59, -0.66) | 952(199, 2902) | 0.34(-0.73, 4.85) | 249(50, 826) | 0.01(-0.33, 0.59) |
| High-income North America | 25433(23557, 27222) | -0.64(-0.63, -0.66) | 24578(22195, 26363) | -0.63(-0.6, -0.66) | 681(197, 1903) | -0.84(-0.94, -0.48) | 174(50, 475) | 0.78(-0.28, 3.52) |
| Caribbean | 117750(80166, 260867) | -0.47(-0.23, -0.61) | 116545(79715, 257968) | -0.47(-0.24, -0.61) | 1033(197, 3154) | -0.3(-0.87, 1.88) | 172(33, 591) | 0.24(-0.35, 1.36) |
| Andean Latin America | 170617(139407, 211393) | -0.76(-0.69, -0.81) | 146707(111590, 185156) | -0.79(-0.72, -0.85) | 20531(7755, 44178) | 0.83(-0.34, 5.04) | 3379(1193, 7724) | 0.52(-0.21, 1.88) |
| Central Latin America | 229591(204643, 259912) | -0.61(-0.56, -0.65) | 215290(187977, 246378) | -0.63(-0.58, -0.68) | 12290(4252, 26888) | 7.19(1.95, 21.69) | 2011(713, 4677) | 0.93(-0.43, 4.56) |
| Tropical Latin America | 230954(219207, 243430) | -0.47(-0.44, -0.49) | 216026(183849, 235138) | -0.5(-0.46, -0.58) | 12826(2562, 37757) | 27.93(3.21, 222.75) | 2102(357, 6509) | 0.76(-0.5, 3.61) |
| North Africa and Middle East | 876482(707940, 1203028) | -0.5(-0.32, -0.6) | 815052(626369, 1119590) | -0.53(-0.37, -0.63) | 57145(17508, 146399) | 6.3(1.47, 21.86) | 4284(1171, 12508) | -0.12(-0.64, 1.26) |
| South Asia | 19297061(17265321, 22142379) | -0.45(-0.3, -0.53) | 16799489(12982490, 19897251) | -0.52(-0.37, -0.63) | 2375114(602437, 5353839) | 33.46(5.8, 164.64) | 122457(30633, 289902) | 0.61(-0.01, 1.48) |
| Central Sub-Saharan Africa | 3374106(2490284, 4750750) | -0.08(0.19, -0.31) | 3212971(2368397, 4622318) | -0.12(0.15, -0.34) | 158952(41334, 539484) | 6.21(0.49, 37.05) | 2183(509, 7665) | 0.31(-0.55, 2.83) |
| Eastern Sub-Saharan Africa | 7096061(5775925, 8662344) | -0.38(-0.23, -0.47) | 6546849(5355243, 8073644) | -0.42(-0.29, -0.53) | 541805(213875, 1115146) | 25.71(8.34, 65.83) | 7407(2792, 15206) | 0.41(-0.45, 1.96) |
| Southern Sub-Saharan Africa | 1767521(1539616, 2072433) | 0.12(0.33, -0.15) | 1637051(1377570, 1944372) | 0.04(0.26, -0.22) | 128716(47514, 287591) | 8.59(1.71, 40.33) | 1754(594, 4156) | 0.10(-0.48, 1.48) |
| Western Sub-Saharan Africa | 4898305(3664376, 6300381) | -0.2(0.05, -0.38) | 4578733(3413713, 5846721) | -0.25(0.00, -0.42) | 315280(110106, 722712) | 8.35(2.7, 24.9) | 4292(1327, 9882) | 0.14(-0.42, 1.28) |
| High-middle SDI | 1419502(1278629, 1615389) | -0.65(-0.7, -0.57) | 1175537(990807, 1374178) | -0.7(-0.76, -0.62) | 182141(104830, 298997) | 0.38(-0.46, 3.34) | 61824(34256, 99131) | -0.51(-0.65, -0.33) |
| High SDI | 297248(268286, 333331) | -0.7(-0.72, -0.65) | 283025(253454, 316346) | -0.7(-0.73, -0.66) | 11677(4897, 23862) | -0.45(-0.72, 0.07) | 2547(1105, 4963) | -0.17(-0.43, 0.29) |
| Low-middle SDI | 19708872(17413627, 22238435) | -0.43(-0.52, -0.29) | 17643651(14611370, 20277479) | -0.49(-0.59, -0.36) | 1950069(640541, 4235607) | 24.05(6.85, 72.46) | 115152(38942, 248890) | 0.31(-0.30, 1.37) |
| Low SDI | 15934129(13382354, 18734417) | -0.32(-0.42, -0.17) | 14650537(12324758, 17376738) | -0.38(-0.48, -0.22) | 1249036(524064, 2482887) | 13.62(6.13, 28.01) | 34555(12527, 73686) | 0.33(-0.28, 1.50) |
| Middle SDI | 9590880(8677173, 11164611) | -0.51(-0.56, -0.37) | 8786088(7660041, 10245125) | -0.54(-0.61, -0.41) | 731287(278634, 1475427) | 1.03(-0.29, 5.77) | 73505(30870, 134892) | -0.05(-0.41, 0.41) |

Notes: Globally, the World Health Organization began to recommend the XDR-TB surveillance in 1991. Consequently, the DALY number cases of XDR-TB has been tracked and reported since 1991. However, the GBD 2021 database provides total percentage change data for the periods 1990－2000, 2000－2021, 1990－2021, 2010－2021, and 2019－2021. Therefore, percentage change of number of DALY cases for XDR-TB of 2010－2021 was used in the study (Abbreviations: DALYs: disability-adjusted life years. DS-TB: drug-susceptible tuberculosis. GBD: Global Burden of Disease. HIV: human immunodeficiency virus. MDR-TB: multidrug-resistant tuberculosis without extensive drug resistance. SDI: Sociodemographic Index. TB: Tuberculosis. UI: Uncertainty interval. XDR-TB: extensively drug-resistant tuberculosis).

Table S6. Predicted ASRs for HIV-DS-TB, HIV-MDR-TB, and HIV-XDR-TB from spanning 2022－2035, based on the Bayesian Age-Period-Cohort Model

| Year | HIV-negative TB | | DS-TB | | MDR-TB | | XDR-TB | |
| --- | --- | --- | --- | --- | --- | --- | --- | --- |
|  | ASIR (per 100,000 population)  (95% *CI*). | ASMR (per 100,000 population)  (95% *CI*). | ASIR (per 100,000 population)  (95% *CI*). | ASMR (per 100,000 population)  (95% *CI*). | ASIR (per 100,000 population)  (95% CI). | ASMR (per 100,000 population)  (95% *CI*). | ASIR (per 100,000 population)  (95% *CI*). | ASMR (per 100,000 population)  (95% *CI*). |
| 2022 | 99.37(96.64, 102.09) | 13.68(13.24, 14.13) | 93.61(91.29, 95.93) | 12.26(11.91, 12.6) | 5.72(4.18, 7.25) | 1.38(1.04, 1.73) | 0.35(0.00, 0.78) | 0.12(0.00, 0.30) |
| 2023 | 97.47(94.11, 100.82) | 13.21(12.66, 13.76) | 91.66(88.87, 94.46) | 11.80(11.38, 12.21) | 6.02(3.71, 8.32) | 1.44(0.93, 1.96) | 0.42(0.00, 1.15) | 0.14(0.00, 0.45) |
| 2024 | 95.61(91.73, 99.50) | 12.76(12.12, 13.39) | 89.77(86.57, 92.97) | 11.35(10.88, 11.82) | 6.33(3.32, 9.35) | 1.51(0.84, 2.17) | 0.51(0.00, 1.60) | 0.17(0.00, 0.64) |
| 2025 | 93.80(89.46, 98.14) | 12.32(11.62, 13.02) | 87.93(84.38, 91.47) | 10.92(10.41, 11.44) | 6.66(2.95, 10.38) | 1.58(0.76, 2.39) | 0.62(0.00, 2.14) | 0.20(0.00, 0.87) |
| 2026 | 92.00(87.26, 96.75) | 11.9(11.14, 12.65) | 86.09(82.24, 89.95) | 10.52(9.96, 11.07) | 7.01(2.59, 11.44) | 1.65(0.68, 2.61) | 0.74(0.00, 2.82) | 0.25(0.00, 1.15) |
| 2027 | 90.20(85.08, 95.31) | 11.49(10.69, 12.3) | 84.26(80.12, 88.40) | 10.12(9.54, 10.71) | 7.38(2.21, 12.55) | 1.72(0.61, 2.84) | 0.89(0.00, 3.67) | 0.30(0.00, 1.51) |
| 2028 | 88.41(82.97, 93.86) | 11.1(10.25, 11.94) | 82.46(78.06, 86.85) | 9.74(9.13, 10.35) | 7.76(1.81, 13.71) | 1.80(0.52, 3.07) | 1.07(0.00, 4.72) | 0.36(0.00, 1.96) |
| 2029 | 86.68(80.92, 92.43) | 10.72(9.84, 11.60) | 80.70(76.06, 85.33) | 9.38(8.75, 10.01) | 8.16(1.39, 14.94) | 1.88(0.44, 3.32) | 1.29(0.00, 6.03) | 0.44(0.00, 2.53) |
| 2030 | 84.98(78.94, 91.02) | 10.35(9.44, 11.26) | 78.99(74.13, 83.84) | 9.03(8.37, 9.68) | 8.59(0.93, 16.24) | 1.96(0.34, 3.58) | 1.55(0.00, 7.67) | 0.53(0.00, 3.24) |
| 2031 | 83.29(76.98, 89.6) | 10.00(9.06, 10.93) | 77.29(72.23, 82.36) | 8.69(8.02, 9.36) | 9.03(0.44, 17.61) | 2.05(0.25, 3.86) | 1.87(0.00, 9.70) | 0.64(0.00, 4.12) |
| 2032 | 81.61(75.05, 88.17) | 9.66(8.70, 10.61) | 75.60(70.34, 80.86) | 8.36(7.69, 9.04) | 9.49(0.00, 19.07) | 2.14(0.14, 4.15) | 2.25(0.00, 12.22) | 0.78(0.00, 5.23) |
| 2033 | 79.95(73.15, 86.75) | 9.32(8.35, 10.30) | 73.95(68.5, 79.39) | 8.05(7.36, 8.74) | 9.98(0.00, 20.61) | 2.24(0.03, 4.45) | 2.71(0.00, 15.36) | 0.94(0.00, 6.62) |
| 2034 | 78.33(71.31, 85.36) | 9.00(8.01, 9.99) | 72.33(66.71, 77.95) | 7.75(7.05, 8.45) | 10.48(0.00, 22.25) | 2.34(0.00, 4.77) | 3.25(0.00, 19.24) | 1.14(0.00, 8.35) |
| 2035 | 76.75(69.51, 83.99) | 8.70(7.69, 9.70) | 70.76(64.96, 76.55) | 7.46(6.75, 8.16) | 11.02(0.00, 23.99) | 2.45(0.00, 5.11) | 3.91(0.00, 24.04) | 1.38(0.00, 10.50) |

Notes: Globally, the World Health Organization began to recommend XDR-TB surveillance in 1991. Consequently, the age-standardized incidence rate of XDR-TB have been tracked and reported since 1991, and the age-standardized mortality rate has been tracked and reported since 1993.When the ASRs is predicted for a given year, if the lower limits of the 95% confidence intervals is below 0, 0 is set (Abbreviations: ASIR: age-standardized incidence rate. ASMR: age-standardized mortality rate. ASR: age-standardized rate. CI: Confidence interval. DS-TB: drug-susceptible tuberculosis. EAPC: annual percentage changes. HIV: human immunodefciency virus. MDR-TB: multidrug-resistant tuberculosis without extensive drug resistance. TB: Tuberculosis. XDR-TB: extensively drug-resistant tuberculosis).


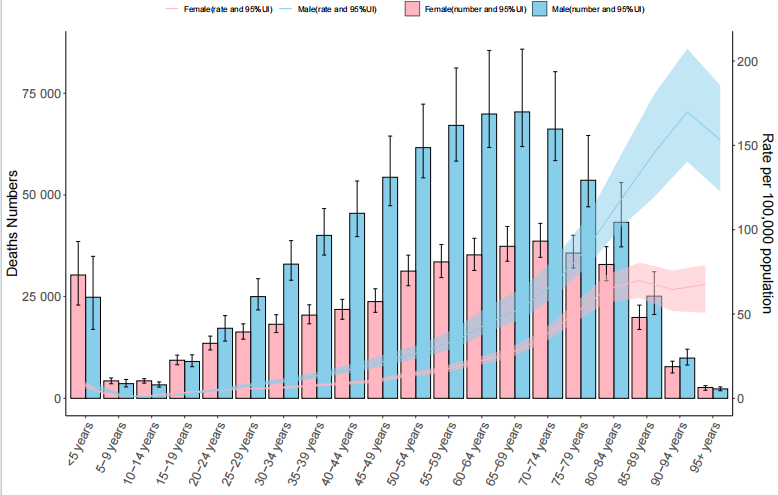


A


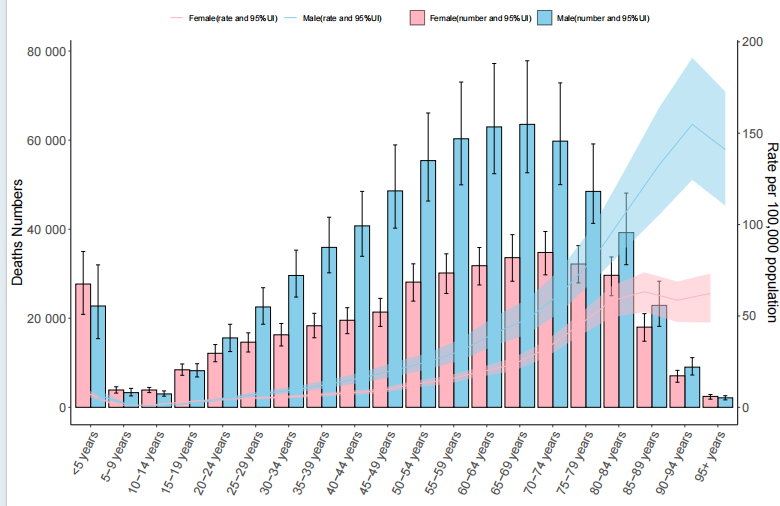


B


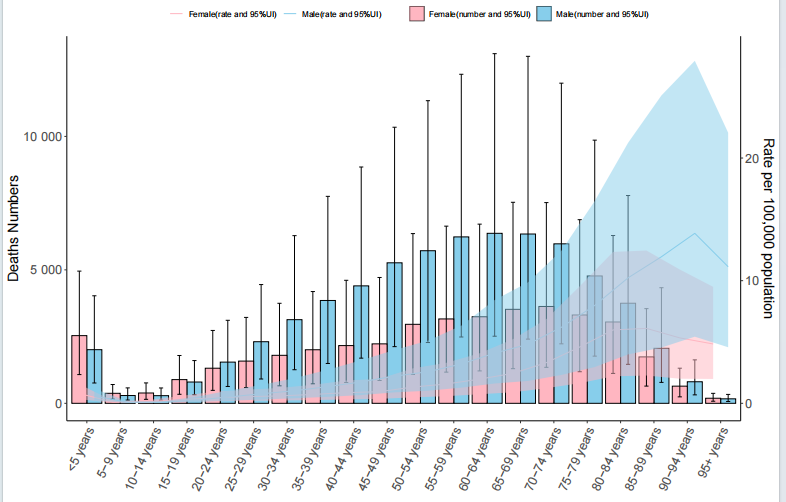


C


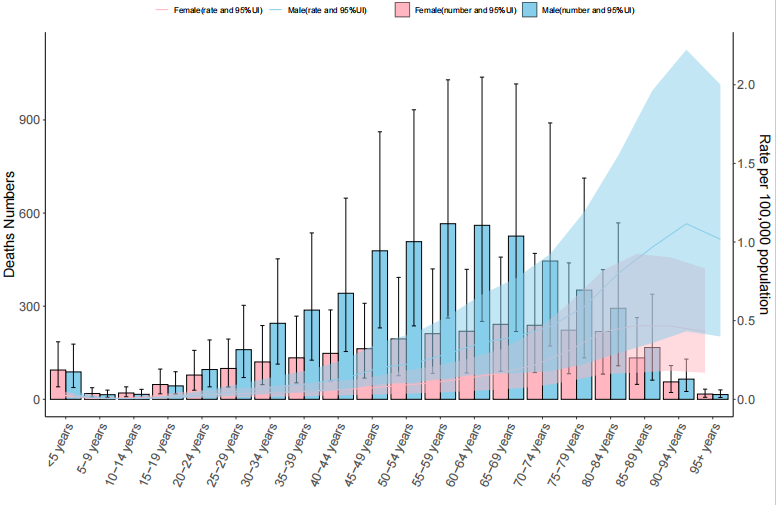


D

Fig. S1. The specific mortality of TB, DS-TB, MDR-TB, and XDR-TB showed notable differences across age and gender distributions in 2021 year ( A: Mortality of TB, B: Mortality of DS-TB. C: Mortality of MDR-TB. D: Mortality of XDR-TB. Abbreviations: DS-TB: drug-susceptible tuberculosis. MDR-TB: multidrug-resistant tuberculosis without extensive drug resistance. TB: Tuberculosis. XDR-TB: extensively drug-resistant tuberculosis).


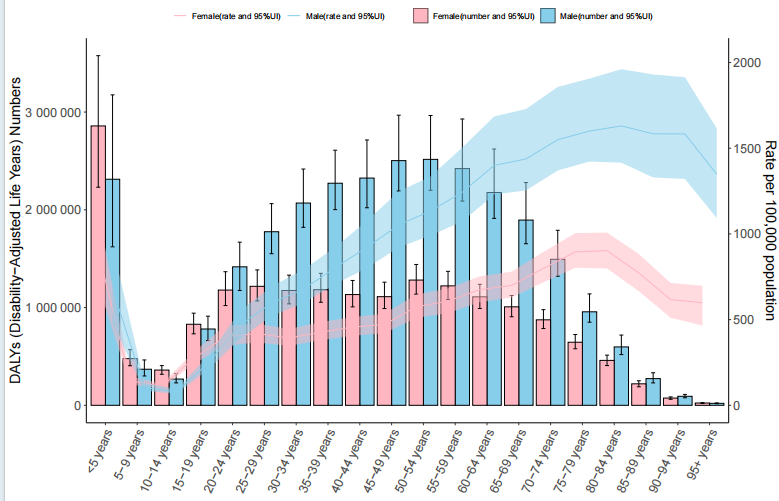


A

B

A


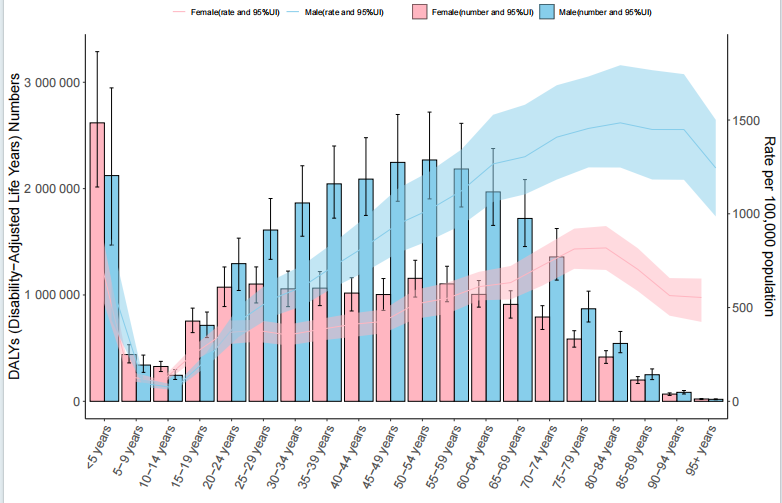

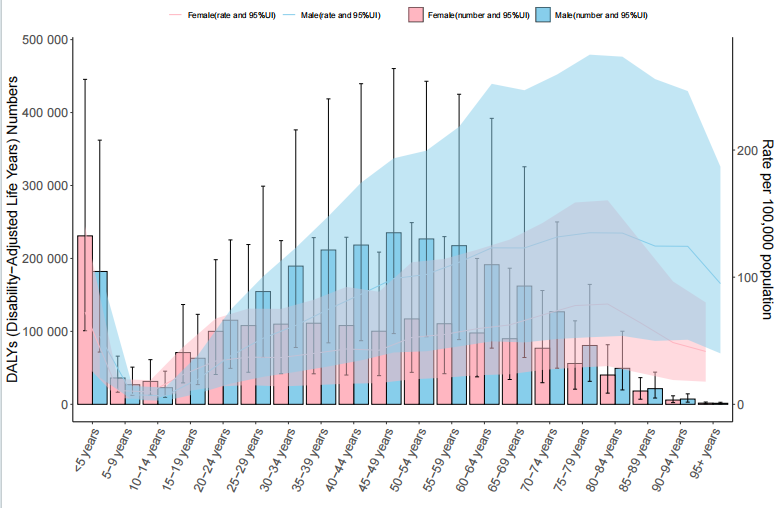


C


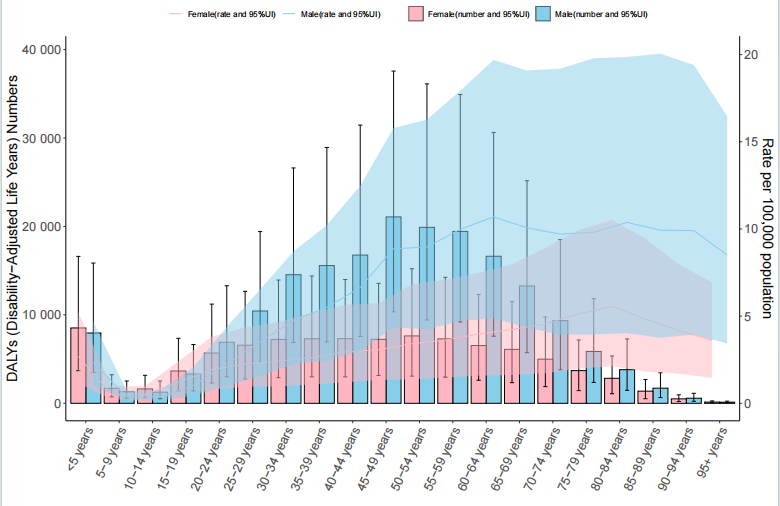


D

Fig.S2. The specific DALY of TB, DS-TB, MDR-TB, and XDR-TB showed notable differences across age and gender distributions in 2021 year ( A: Mortality of TB, B: Mortality of DS-TB. C: Mortality of MDR-TB. D: Mortality of XDR-TB. Abbreviations: DALYs: disability-adjusted life years. DS-TB: drug-susceptible tuberculosis. MDR-TB: multidrug-resistant tuberculosis without extensive drug resistance. TB: Tuberculosis. XDR-TB: extensively drug-resistant tuberculosis)


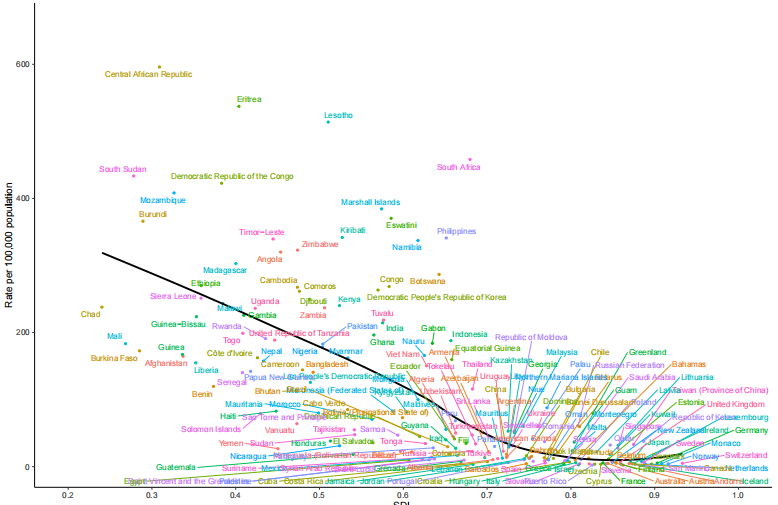

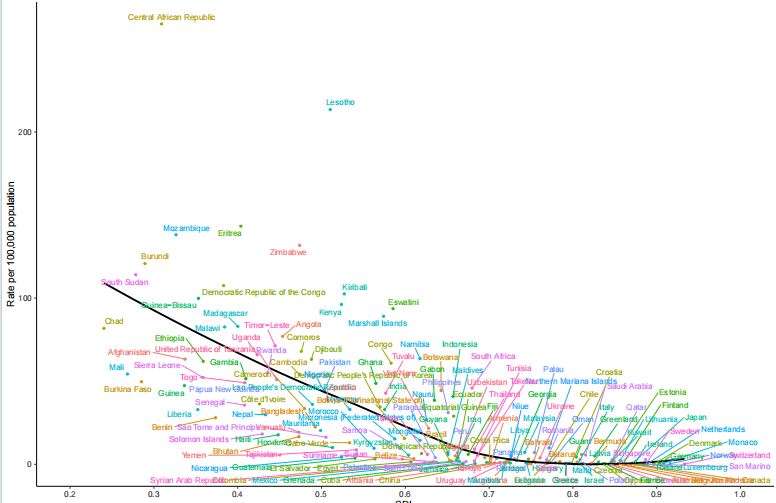

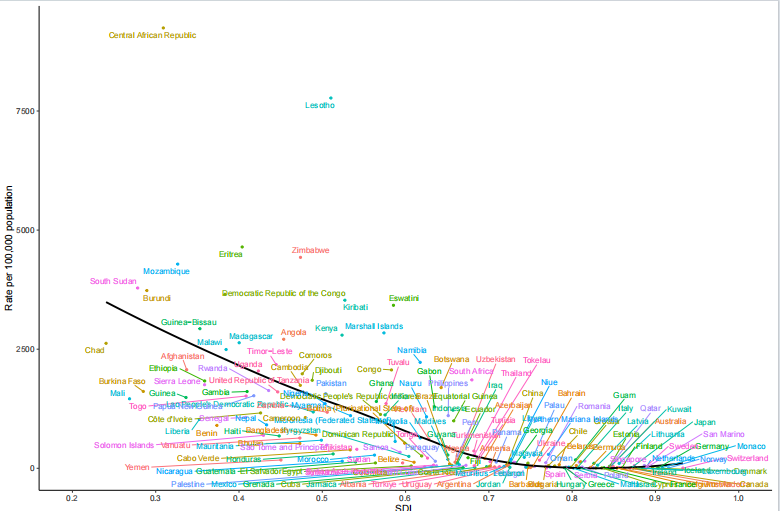


C

B

A

Fig.S3. The association between the SDI and the ASIR, ASMR, and age-standardized DALY rate of TB across 204 countries and territories in 2021 year (A: age-standardized incidence rate of TB. B: age-standardized mortality rate of TB. C: age-standardized DALY rate of TB). (Abbreviations: DALYs: disability-adjusted life years. TB: tuberculosis).


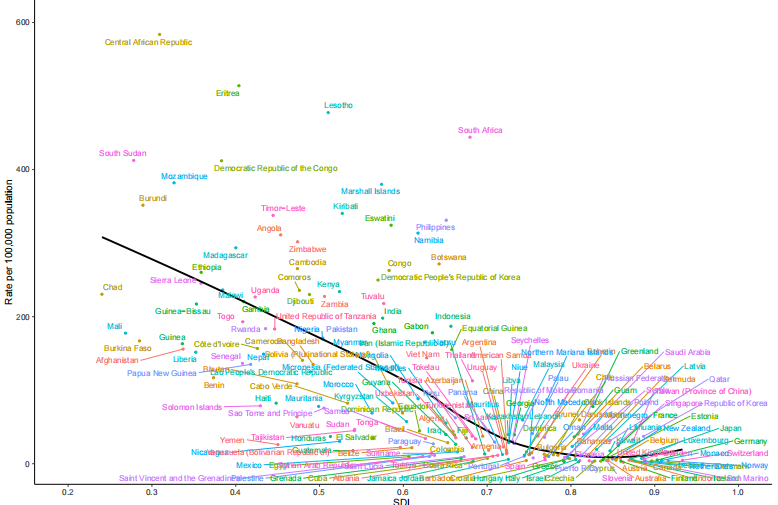

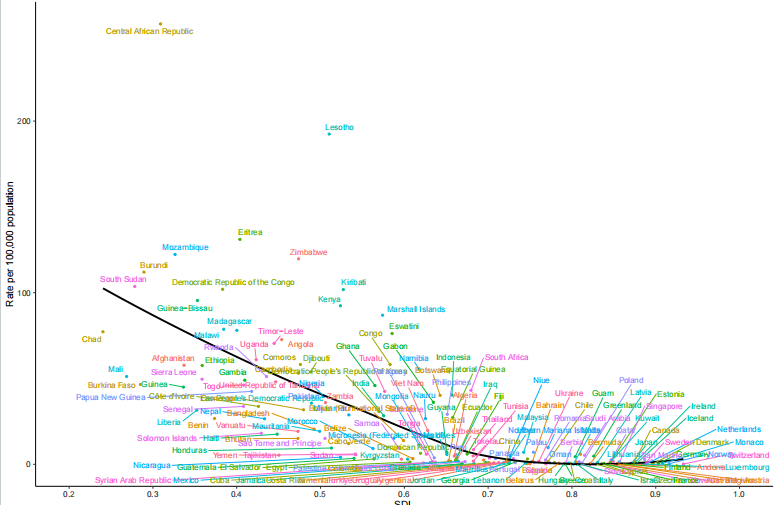

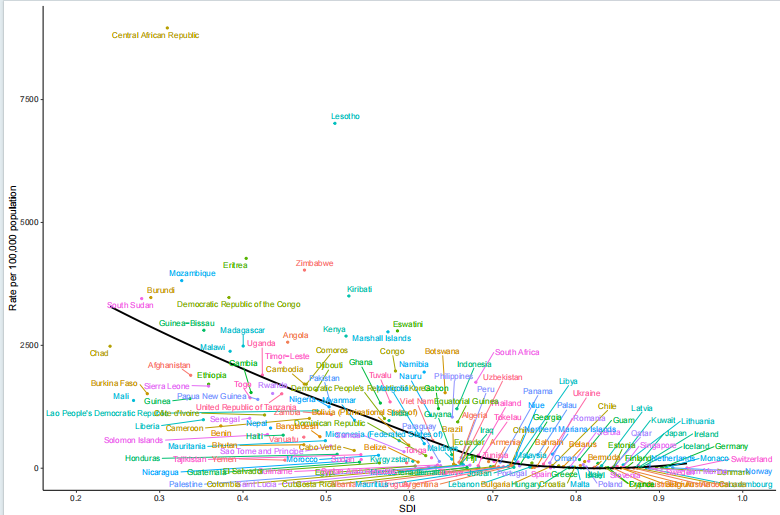


C

B

A

Fig.S4. The association between the SDI and the ASIR, ASMR, and age-standardized DALY rate of DS-TB across 204 countries and territories in 2021 year (A: age-standardized incidence rate of DS-TB. B: age-standardized mortality rate of DS-TB. C: age-standardized DALY rate of DS-TB). (Abbreviations: DALYs: disability-adjusted life years. DS-TB: drug-susceptible tuberculosis).


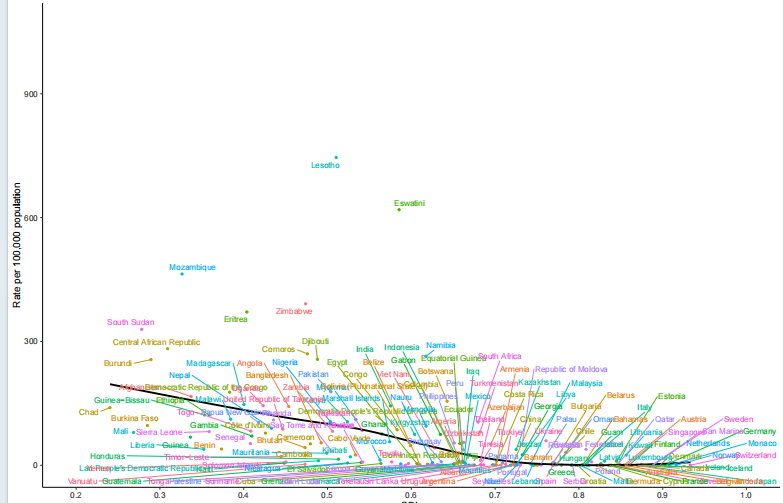

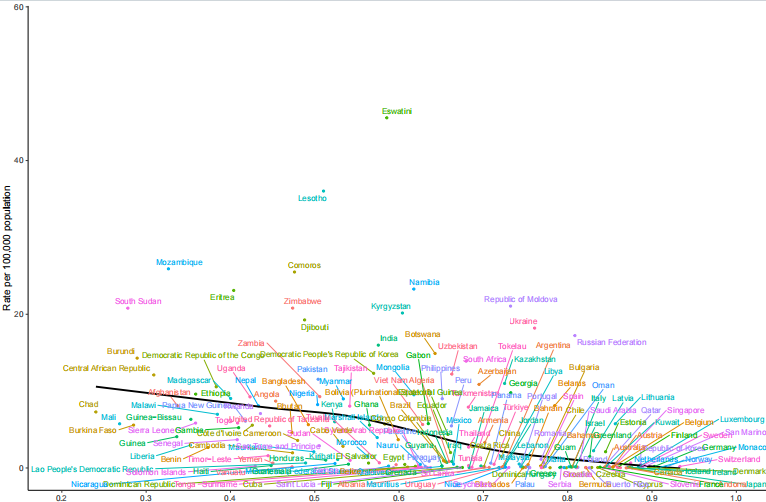

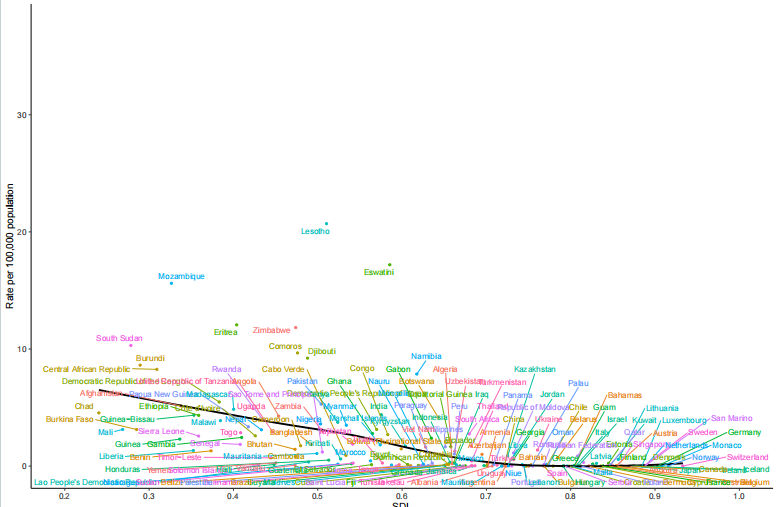


C

B

A

Fig.S5. The association between the SDI and the ASIR, ASMR, and age-standardized DALY rate of MDR-TB across 204 countries and territories in 2021 year (A: age-standardized incidence rate of MDR-TB. B: age-standardized mortality rate of MDR-TB. C: age-standardized DALY rate of MDR-TB). (Abbreviations: DALYs: disability-adjusted life years. MDR-TB: multidrug-resistant tuberculosis without extensive drug resistance).


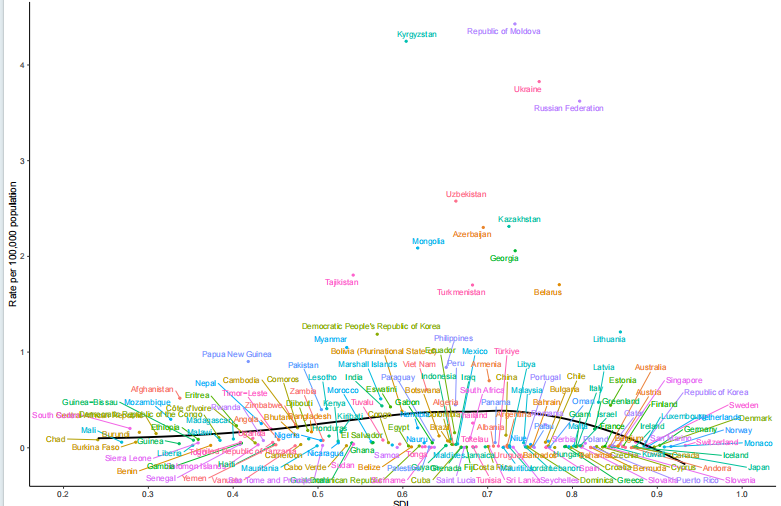

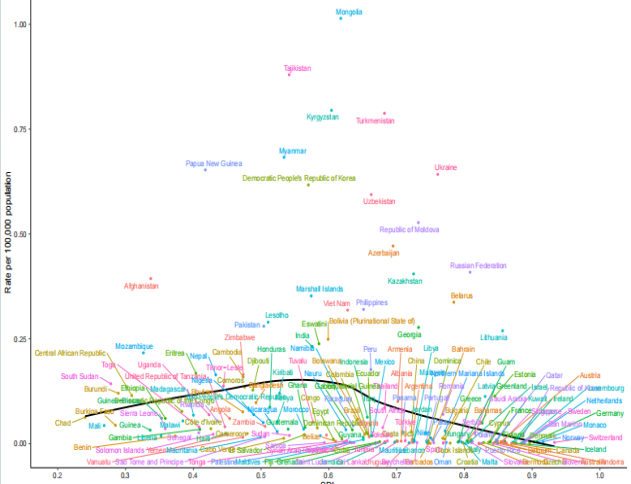

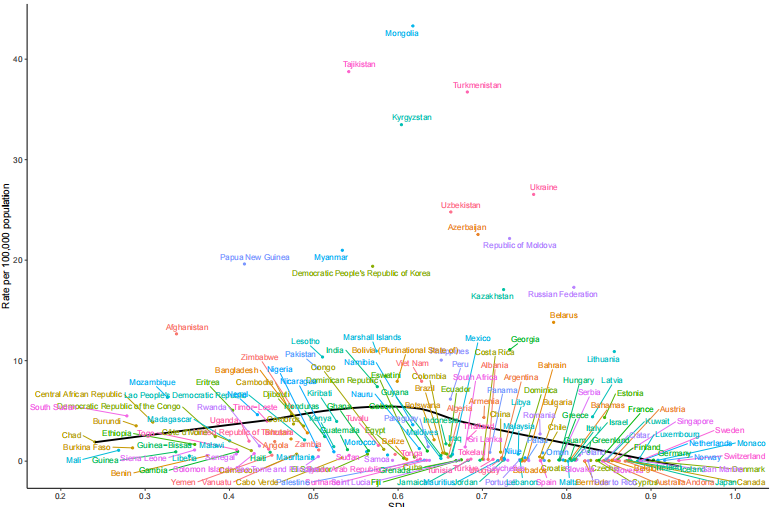


C

B

A

Fig. S6. The association between the SDI and the ASIR, ASMR, and age-standardized DALY rate of XDR-TB across 204 countries and territories in 2021(A: age-standardized incidence rate of XDR-TB. B: age-standardized mortality rate of XDR-TB. C: age-standardized DALY rate of XDR-TB). (Abbreviations: DALYs: disability-adjusted life years. XDR-TB: extensively drug-resistant tuberculosis).


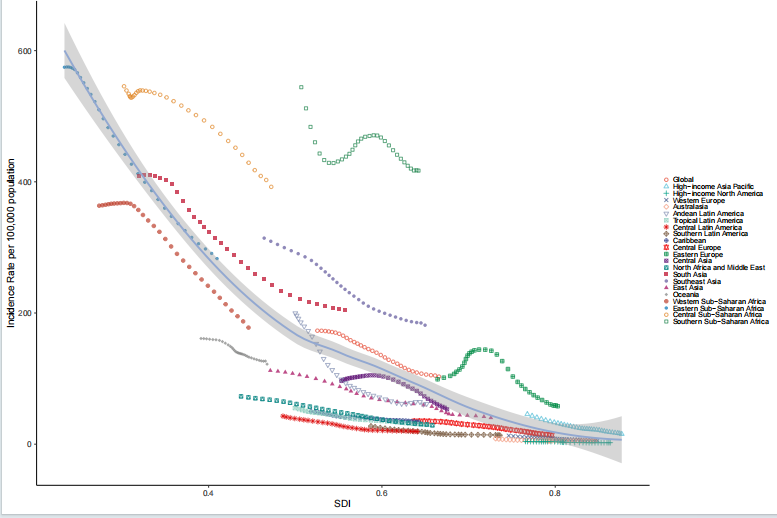

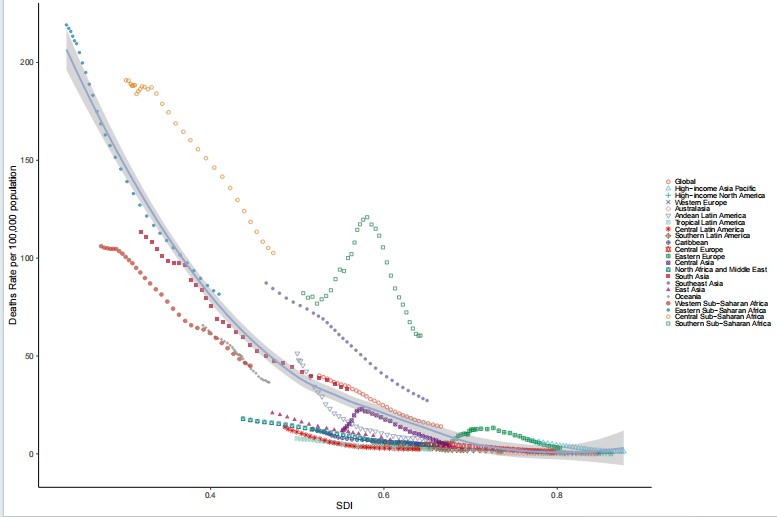

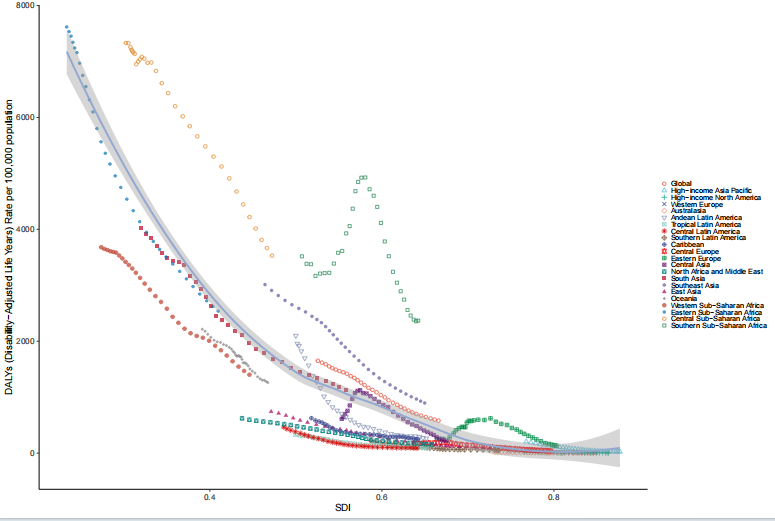


C

B

A

Fig.S7. The association between the SDI and the ASIR, ASMR, and age-standardized DALY rate from 1990 to 2021 year (A: Incidence rate of TB, B: mortality rate of TB. C: DALY rate of TB. Abbreviations: DALYs: disability-adjusted

life years. TB: Tuberculosis.).


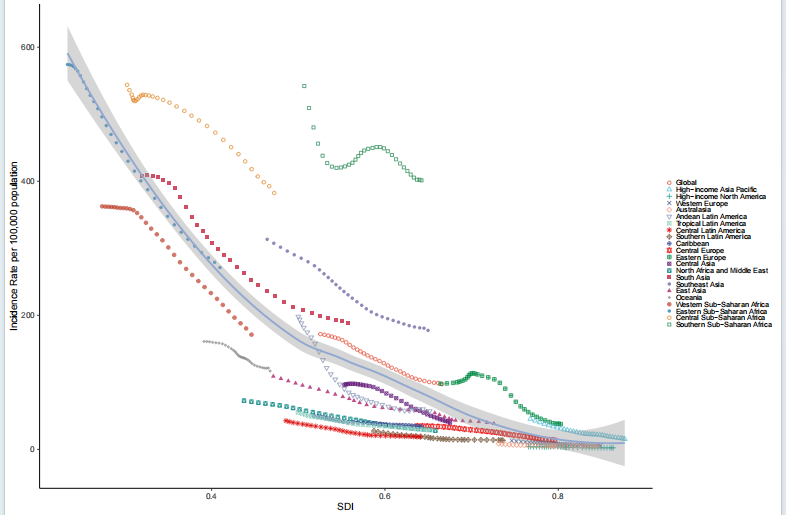

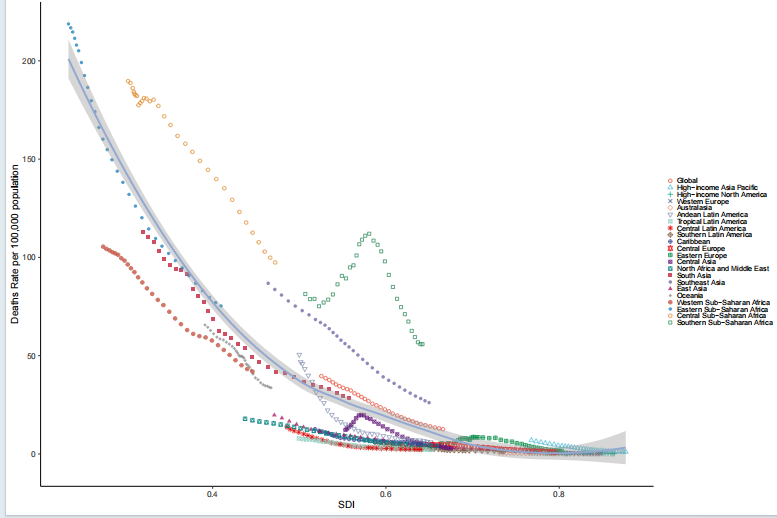

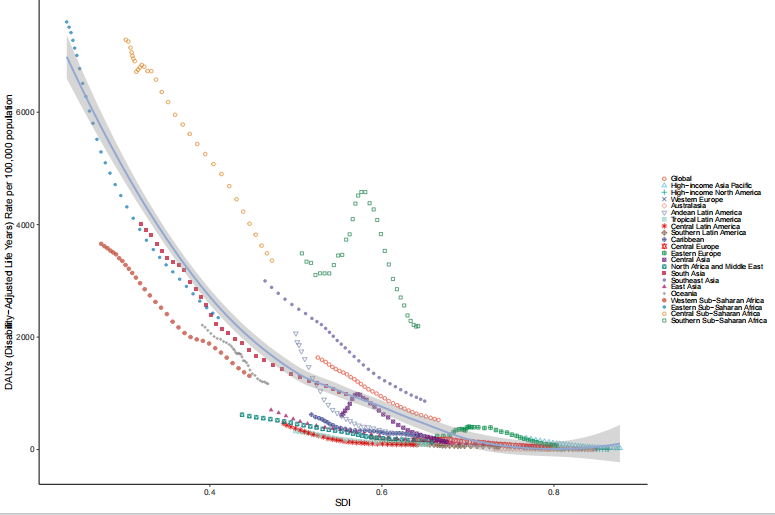


C

B

A

Fig. S8. The association between the SDI and the ASIR, ASMR, and age-standardized DALY rate of DS-TB from 1990 to 2021 year (A: Incidence rate of DS-TB, B: mortality rate of DS-TB. C: DALY rate of DS-TB. Abbreviations: DALYs: disability-adjusted life years. DS-TB: drug-susceptible tuberculosis).


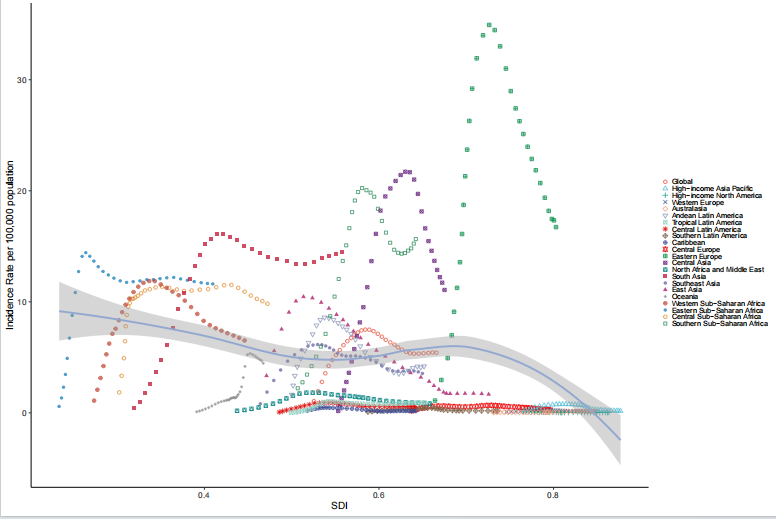

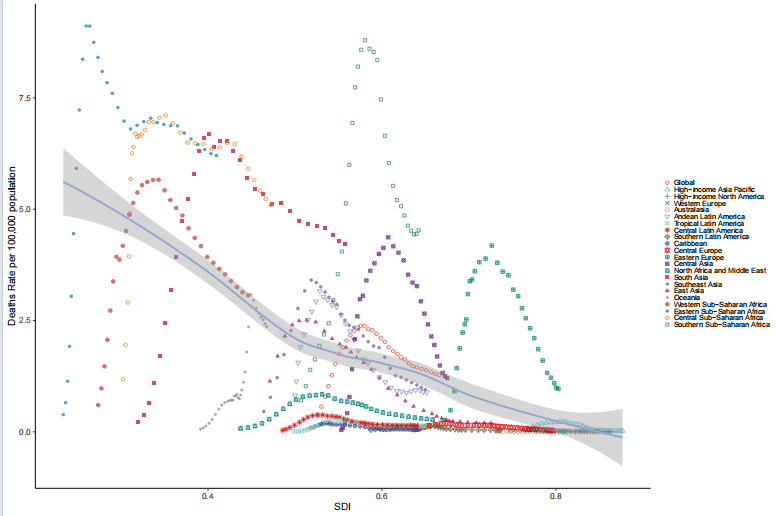

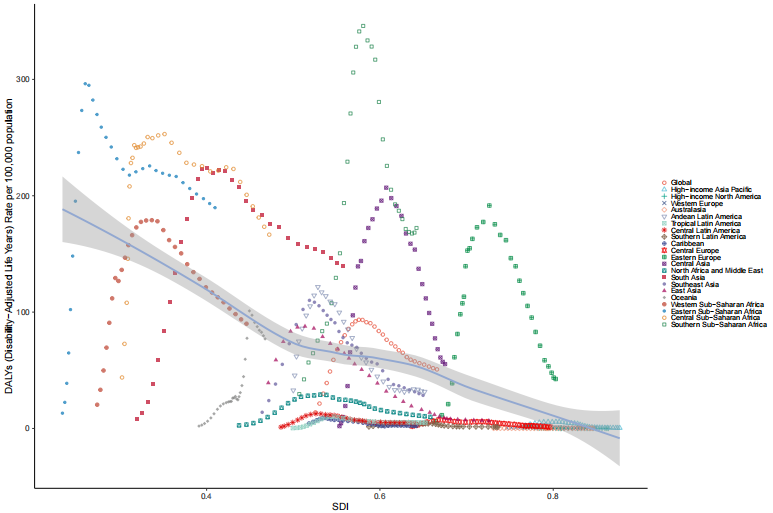


C

B

A

Fig.S9. The association between the SDI and the ASIR, ASMR, and age-standardized DALY rate of MDR-TB from 1990 to 2021 year (A: Incidence rate of MDR-TB, B: mortality rate of MDR-TB. C: DALY rate of MDR-TB. Abbreviations: DALYs: disability-adjusted life years. MDR-TB: multidrug-resistant tuberculosis without extensive drug resistance).


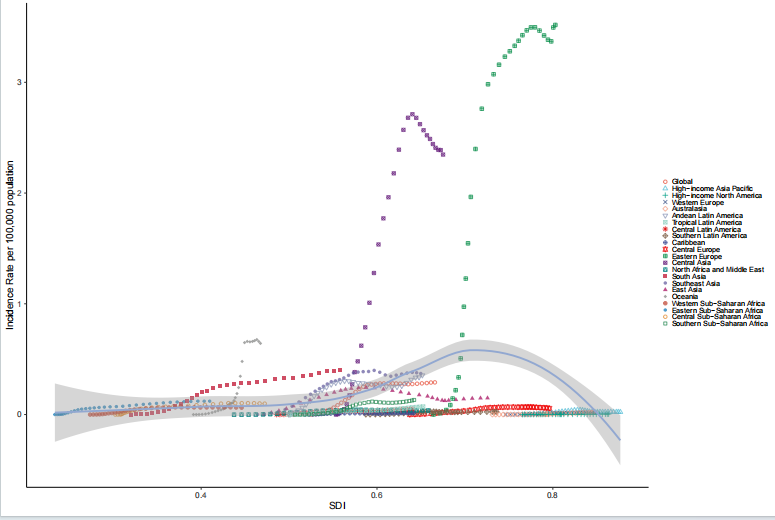

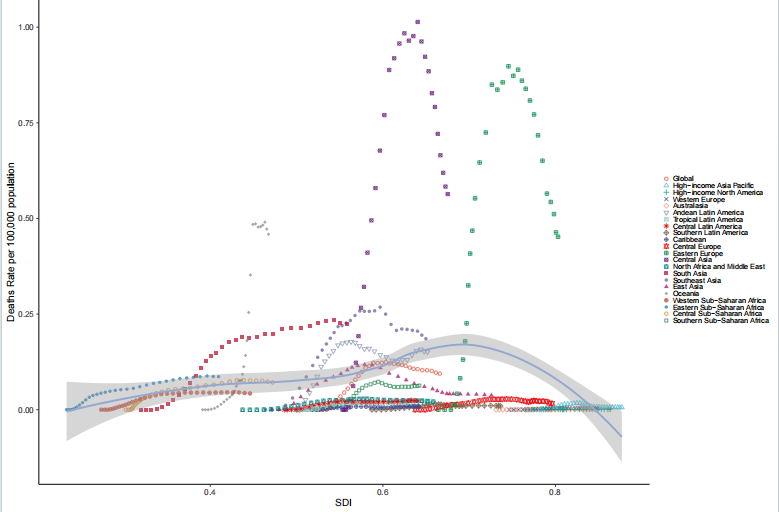

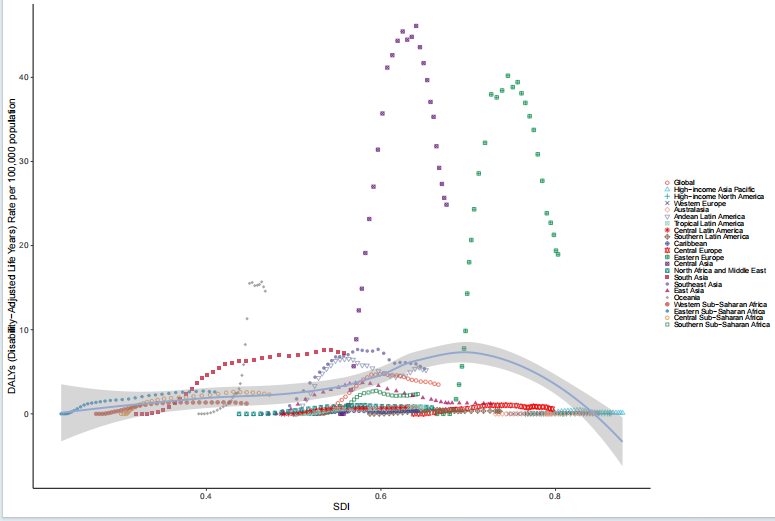


C

B

A

Fig. S10. The association between the SDI and the ASIR, ASMR, and age-standardized DALY rate of XDR-TB from 1990 to 2021 year (A: Incidence rate of XDR-TB, B: mortality rate of XDR-TB. C: DALY rate of XDR-TB. Abbreviations: DALYs: disability-adjusted life years. XDR-TB: extensively drug-resistant tuberculosis).


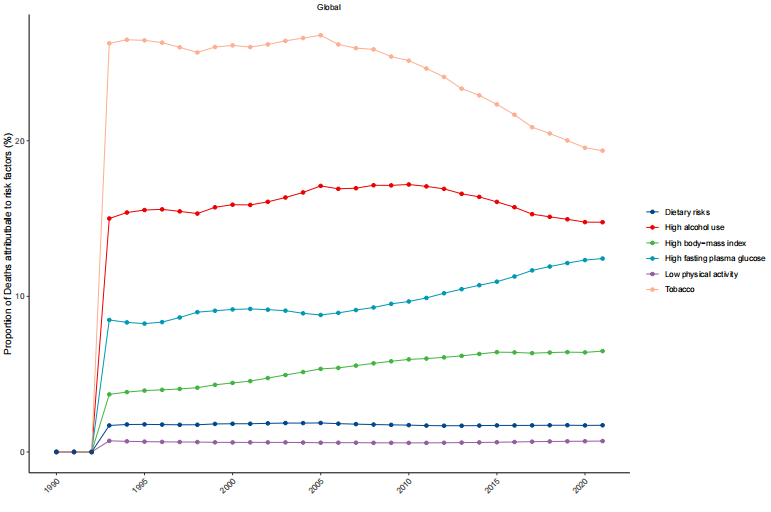


A


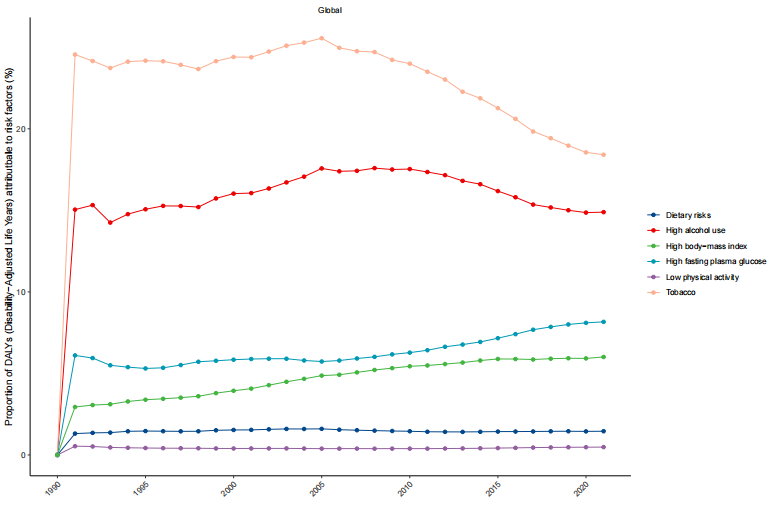


B

Fig. S11. The association between risk factors and the ASMR and age-standardized DALY rate of XDR-TB in 21 GBD regions from 1990 to 2021 (A: mortality rate of XDR-TB. B: DALY rate of XDR-TB. Abbreviations: DALYs: disability-adjusted life years. GBD: Global Burden of Disease. XDR-TB: extensively drug-resistant tuberculosis).
